# Hidden variation in polyploid wheat drives local adaptation

**DOI:** 10.1101/gr.233551.117

**Published:** 2018-09

**Authors:** Laura-Jayne Gardiner, Ryan Joynson, Jimmy Omony, Rachel Rusholme-Pilcher, Lisa Olohan, Daniel Lang, Caihong Bai, Malcolm Hawkesford, David Salt, Manuel Spannagl, Klaus F.X. Mayer, John Kenny, Michael Bevan, Neil Hall, Anthony Hall

**Affiliations:** 1 Earlham Institute, Norwich, NR4 7UZ, United Kingdom;; 2 HelmholtzZentrum München, German Research Center for Environmental Health, Munich, 85764, Germany;; 3 Institute of Integrative Biology, University of Liverpool, Liverpool, L69 7ZB, United Kingdom;; 4 Rothamsted Research, Harpenden, AL5 2JQ, United Kingdom;; 5 University of Nottingham, Sutton Bonington Campus, Sutton Bonington, LE12 5RD, United Kingdom;; 6 Wissenschaftszentrum Weihenstephan (WZW), Technical University Munich, Freising, 85354, Germany;; 7 John Innes Centre, Norwich, NR4 7UH, United Kingdom;; 8 School of Biological Sciences, University of East Anglia, Norwich, NR4 7TJ, United Kingdom

## Abstract

Wheat has been domesticated into a large number of agricultural environments and has the ability to adapt to diverse environments. To understand this process, we survey genotype, repeat content, and DNA methylation across a bread wheat landrace collection representing global genetic diversity. We identify independent variation in methylation, genotype, and transposon copy number. We show that these, so far unexploited, sources of variation have had a significant impact on the wheat genome and that ancestral methylation states become preferentially “hard coded” as single nucleotide polymorphisms (SNPs) via 5-methylcytosine deamination. These mechanisms also drive local adaption, impacting important traits such as heading date and salt tolerance. Methylation and transposon diversity could therefore be used alongside SNP-based markers for breeding.

One of the most important questions in plant breeding is the nature of the genomic variation that has been selected for improving phenotypes. Although it is likely that all forms of genomic change contribute to performance variation and to hybrid vigor, the role of epigenetic variation in crop improvement is not well understood, despite being widespread and highly variable (Springer and Schmitz 2017). It is now clear that epigenetic variation can be stably inherited and that spontaneous epialleles are rare (Johannes et al. 2009; Hofmeister et al. 2017). Therefore, epigenetic variants could potentially be used in breeding programs and their contributions to trait variation assessed alongside classical genetic variation. To identify new sources of variation for crop improvement and to understand the contributions of variation to traits, it is important to assess both genomic and epigenetic variation in crop species.

Epigenetic states of genes in crop plants have been shown to have a major influence on traits. Gene body methylation (gbM) can influence splice-site efficiency by differential methylation of splice acceptor sites, indicating that epiallelic variation can contribute to differential mRNA accumulation (Regulski et al. 2013). In domesticated polyploid cotton and wild relatives, there is extensive epigenetic variation, with methylation differences between homoeologous genes. One example is *COL2D* that is repressed by methylation in wild relatives but is activated by loss of methylation in allotetraploid cotton, influencing flowering time in domesticated lines (Song et al. 2017). The causal gene of a major QTL enhancing resistance to maize stalk rot*, ZmCCT*, is in two epigenetic states. One has a CACTA-like transposable element (TE) upstream of the *ZmCCT* promoter and one without that has enriched methylated CG that suppressed expression and increased disease susceptibility (Wang et al. 2017). Similar mechanisms of epigenetic change in gene expression mediated by retrotransposons adjacent to promoters have also been noted in wheat (Kashkush et al. 2002). Tissue-culture induced reduction in methylation of a retrotransposon in the intron of an oil palm *DEFICIENS* gene alters splicing and causes premature termination (Ong-Abdullah et al. 2015). This epigenetic mechanism contributes to the mantled phenotype that limits clonal propagation of this key global crop.

Analyses of DNA methylation patterns in numerous plant accessions and species are starting to reveal the extent of epigenetic variation and the mechanisms involved in generating and maintaining it. In plants, cytosine methylation of DNA occurs typically at CpG residues but can also occur at CHG and CHH sites (where H represents adenine, cytosine, or thymine). Two general patterns of DNA methylation have been identified in plants—transposable element methylation patterns (teM) and gene body methylation patterns. In *Arabidopsis thaliana* accessions, it was shown that increased gbM is related to constitutive gene expression patterns and that teM epialleles of genes tend to be expressed at lower levels. Geographic origin was a major predictor of DNA methylation levels and of altered gene expression caused by epialleles (Dubin et al. 2015; Kawakatsu et al. 2016). It is clear that natural epigenetic variation provides a source of phenotypic diversity alongside genetic variation; however, currently, little is known about this epigenetic variation and its interaction with genetic diversity in hexaploid wheat populations.

The genomes of crop plants such as maize and wheat are mainly composed of massive tracts of diverse retroelements and DNA repeats that comprise up to 80% of the genome. These repeats are highly methylated to suppress expression and transposition to maintain genome stability (Kim and Zilberman 2014). Wheat is an allopolyploid, comprised of three independently maintained A, B, and D subgenomes that are functionally diploid (Marcussen et al. 2014). Epigenetic mechanisms have been invoked to explain the emergence of key agronomic traits upon formation of hexaploid bread wheat and to explain alterations in gene expression of homoeologous genes upon polyploidization (Song and Chen 2015). Previously, we showed that methylation patterns differ across the A, B, and D subgenomes and in broad terms reflect patterns of methylation of progenitor species (Gardiner et al. 2015). Here, we extend our analyses to a core collection of diverse bread wheat landraces in the Watkins collection (Wingen et al. 2014). Landraces are locally adapted wheat varieties that have not been subject to selective breeding and represent a pool of diversity reflecting their wide adaptation to different growing environments. Such diversity is beginning to be used in breeding programs; therefore, it is timely to assess and understand both the genomic and epigenomic diversity in this population. We hypothesize that the Watkins collection will be not only genetically but epigenetically diverse. As such, we assess this epigenetic diversity and determine if, similar to *Arabidopsis*, it is linked to geographical origin or environmental factors.

## Results

### Methylation and genotype analysis across a wheat landrace diversity panel

To study epigenetic variation across gene-rich regions of the 17-Gb allohexaploid wheat genome, we used genomic enrichment (Agilent SureSelect) followed by bisulfite treatment and Illumina HiSeq paired-end sequencing. Capture probes were designed (12-Mb capture targeting 36 Mb) as described in our previous work (Supplemental Fig. S1 from Olohan et al. 2018).

To accurately apply MethylSeq to a diversity panel, we require bisulfite-treated and untreated sequence data for each wheat accession to identify C-T SNPs, which would otherwise be incorrectly classified as unmethylated cytosines. This was achieved using a modified sequence capture protocol (Darst et al. 2010; Olohan et al. 2018) that generates two libraries for sequencing from one capture; a bisulfite-treated and an untreated library for each accession. Post-sequencing, the untreated data sets were aligned to the TGAC v1 Chinese Spring reference sequence, and SNP calling was performed (Methods; Clavijo et al. 2017). We identified 716,018 SNPs, on average, per accession at ≥5×, of which 316,767 were homozygous. The homozygous SNPs from each Watkins accession individually were used to correct the Chinese Spring reference genome to generate an accession-specific reference sequence for each of the 104 lines; this corrected reference was then implemented for mapping the corresponding bisulfite-treated data set (Methods).

Bisulfite-treated DNA from single seedlings was examined for 104 core lines from the Watkins landrace collection plus the reference variety Chinese Spring (Supplemental Table S1; Supplemental Note S1A). We scored methylation at an average of 10.9 M cytosines per accession (Supplemental Table S2; Supplemental Note S1B), and across all accessions, on average 98.7% of cytosine bases were successfully bisulfite-converted (Supplemental Table S3; Supplemental Note S1C).

### Genetic variation across the Watkins collection clusters across large geographical regions

**Figure 1.**
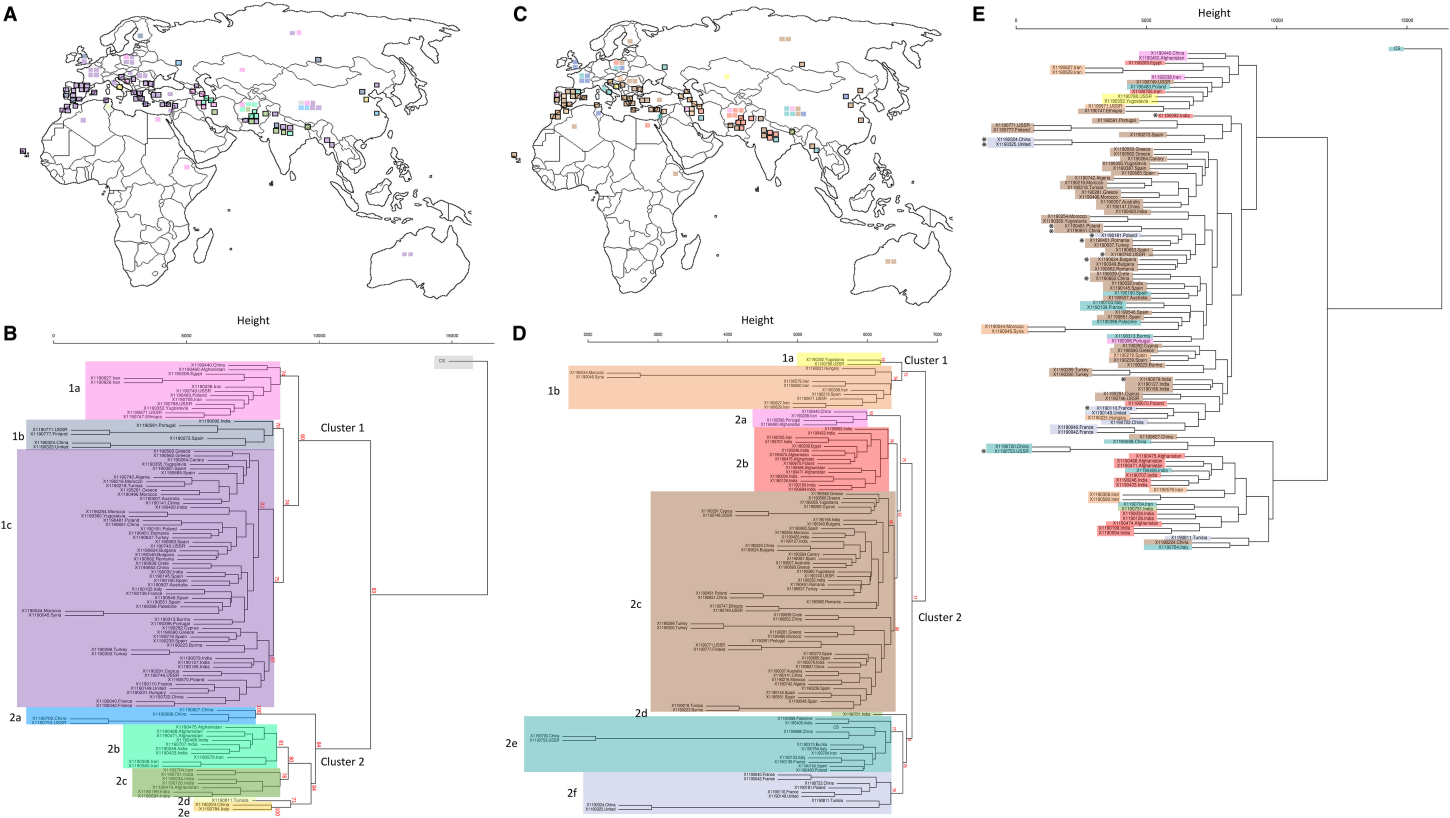
Geographical origins combined with hierarchical cluster analysis on 104 accessions from the Watkins core collection plus Chinese Spring wheat. (*A*) Geographical positions of the accessions color-coded by their allocated cluster from *B* after SNP hierarchical clustering. (*B*) Dendrogram constructed using the complete linkage method within the R package *hclust* to cluster accessions based on SNP allele frequency across 53,341 SNP sites. The tree was cut into eight groups (excluding the reference Chinese Spring) using the R package cutree, and these clusters are color-coded (Methods). (*C*) Geographical positions of the accessions color-coded by their allocated cluster from *D* after CpG SMP hierarchical clustering. (*D*) Dendrogram constructed using the complete linkage method within the R package *hclust* to cluster accessions based on methylation levels across 18,965 CpG SMP sites (taken from the 359,500 SMPs that were identified). The tree was cut into eight groups using the R package cutree, and these clusters are color-coded (Methods). (*E*) SNP-based dendrogram from *B* with individual accessions color-coded as per their cluster from the SMP-based dendrogram from *D*. For geographical accession positions in *A* and *C*, squares outlined in black represent accessions with detailed positional information that is used for plotting; squares with no outline represent accessions with only a country of origin. AU *P*-values were computed for the main clusters in *B* and *D* using the R package *pvclust* and are shown in red (Methods).

From the 716,018 SNPs that were identified on average per accession, 53,341 SNP sites were identified across the 105 accessions, where all showed mapping coverage at ≥5×, and ≥1 accession had a SNP. For each SNP, the alternate allele frequency per accession was used for hierarchical clustering of the accessions (Methods). Using genotype information for clustering, accessions originating from Europe and the Mediterranean tend to cluster together, while accessions from larger geographic regions in Asia and Russia show higher diversity (Fig. 1A,B).

The Watkins collection clusters into two main ancestral groups; cluster 1 with 80 accessions (73.8% derived from Europe, Middle Eastern, and South Mediterranean/African regions), while cluster 2 has 24 accessions (87.5% mainly Asian) (Supplemental Table S4). Additional clustering models supported this genotype-based population structure and it also resembles that from previous analyses of the Watkins collection using array SNP data (see Supplemental Note S2; Supplemental Fig. S1; Wingen et al. 2014; Winfield et al. 2017).

### SMPs are variable and methylation profiles of accessions from smaller countries are more likely to cluster together

Global methylation patterns in Chinese Spring align closely to those of other plant species and previous analyses of Chinese Spring (Supplemental Note S3; Supplemental Fig. S2; Supplemental Table S5; Li et al. 2012; Gardiner et al. 2015). To assess epigenetic variation across the Watkins collection, we identified 853,932 cytosines that were mapped to ≥10× in all 104 accessions plus Chinese Spring. Of these cytosines, 359,500 (42.1%) were classified as single methylation polymorphism sites (SMPs) between the accessions (Supplemental Table S6; Methods). Although methylation variability is high, the SMPs do not preferentially target any of the methylation contexts (CpG, CHG, or CHH) (Supplemental Table S6).

Interrogation of the 359,500 SMP sites showed that 0.5% had high methylation conservation between accessions (methylated in ≥90%); these were mainly at CpG sites (86.2%) with a bias for transcribed regions (80.2%). Focusing on CpG sites, 13.9% of SMPs were methylated in ≥90 accessions, highlighting the increased stability of CpG sites compared to non-CpG sites. However, most SMPs (91.5%) are rare variants in <10% of the accessions. Unlike highly conserved SMPs, these low-frequency SMPs show less bias for transcribed regions (74.2%) and increased bias for non-CpG sites potentially due to the more dynamic tissue specificity of this methylation (82.5% at CHH sites and 16.4% at CHG sites). Accession-specific SMPs were identified from the 359,500 SMPs (Methods); on average, each accession showed methylation at 26,980 SMP positions, with a range of 11,279–64,659 SMPs per accession (Supplemental Table S7).

**Figure 2.**
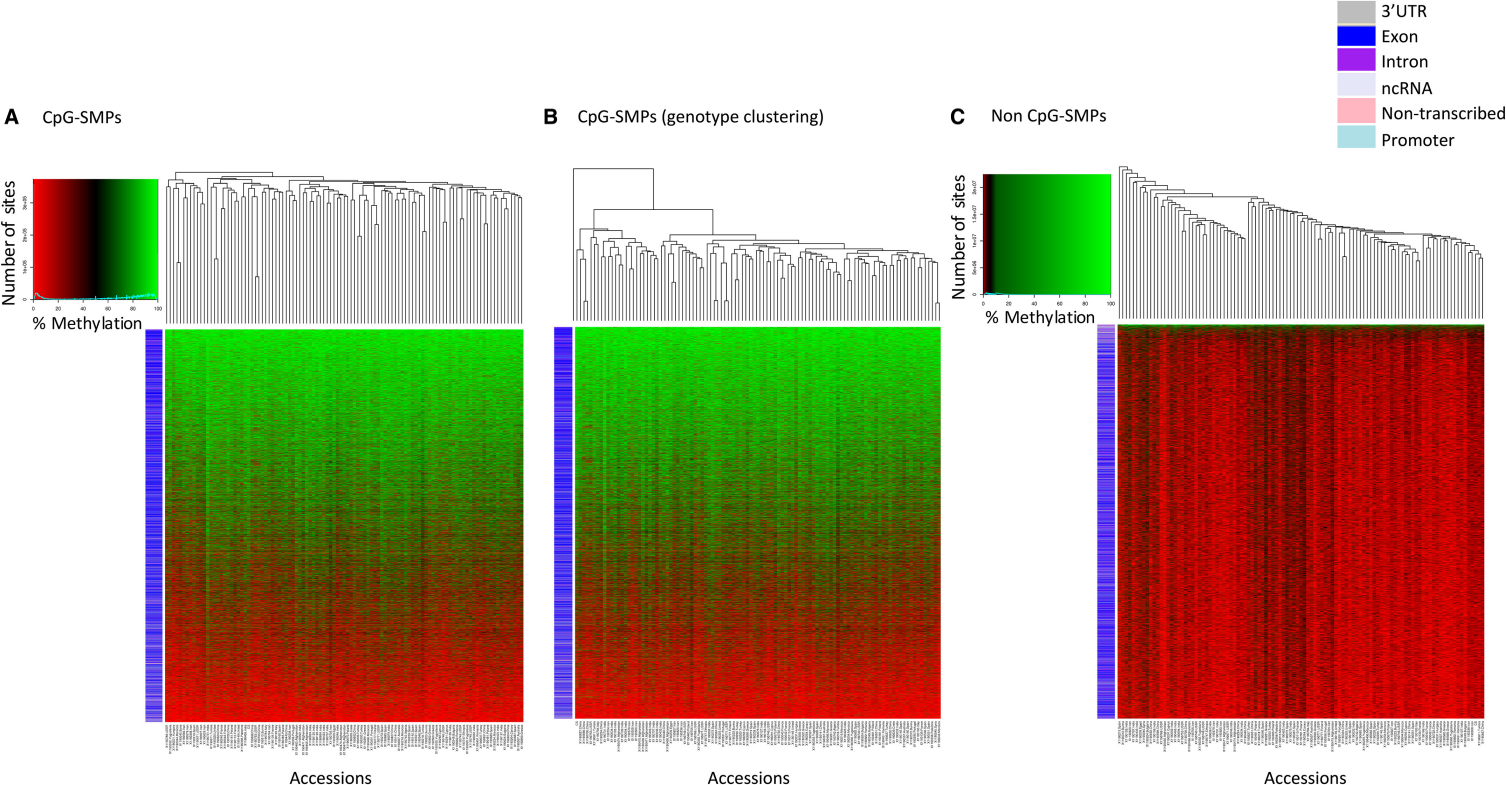
Visualizing methylation levels for the 105 wheat accessions across 359,500 SMP sites. Using sites with coverage in all 104 Watkins collection accessions plus Chinese Spring, we generated heat maps for methylation levels across (*A*) CpG-SMPs, (*B*) CpG-SMPs with accessions ordered by genotype using the heat map from *A* with accessions reordered based on Figure 1B's SNP clustering dendrogram (shown on *top* horizontal axis), and (*C*) non-CpG SMPs. Rows correspond to individual SMP sites and columns indicate accessions. The colored row labels (barcodes) on the *left* of the heat map indicate which genomic location a SMP falls into (see legend). SMP sites are ordered by their total methylation across the accessions on the vertical axes, and accessions are clustered by SMP profiles on the horizontal axes (Methods).

To analyze inter-accession variation in SMPs, for all 359,500 SMP sites, epiallele frequency per accession was used for hierarchical clustering for CpG and non-CpG sites individually (Fig. 2; Supplemental Note S4; Supplemental Fig. S3). When we order SMP sites by their total methylation across the accessions (Fig. 2, vertical axes), for CpG sites, there is a tendency for sites to show extremes of either high- or low-level methylation, with typically more methylation in transcribed regions and less methylation in nontranscribed regions. Conversely, non-CpG SMP sites tend to show higher methylation in nontranscribed regions. Clustering the data sets by accession (Fig. 2, horizontal axes), inter-accession variation is less obvious for non-CpG sites, where most of the methylation is low-level or potentially tissue-specific (Fig. 2C). However, more inter-accession methylation variation can be observed at CpG sites with both high- and low-level methylation; therefore, accessions can be informatively compared (Fig. 2A).

For CpG methylation, hierarchical clustering of accessions correlates with their geographical proximity. Accessions from within the same country of origin tend to show higher linkage and cluster together closely, with 90.3% of the 31 regions analyzed containing a majority of accessions (≥50%) from one linkage cluster (Fig. 1C,D). From the top hierarchical level epigenetic population structure of the Watkins collection, accessions cluster into two groups composed largely of accessions from mixed geographic locations; cluster 1 containing 12 accessions derived from 50% Asian and 50% European/Middle Eastern locations, and cluster 2 containing 93 accessions derived from 41% Asian and 59% European/Middle Eastern locations (Fig. 1; Supplemental Table S8). This population structure differs from the genotype-based population structure (Fig. 1E); although both split into two subpopulations at the top hierarchical level, for genotype, these subpopulations showed one population from mixed geographic locations, while the other was of Asian origin. We statistically compared the two cluster configurations (Fig. 1B,D); the cluster configuration in the combined SMP and SNP trees (Fig. 1E) was nonrandom (one-sample runs test with 39 runs: *Z* = −2.53, *P* = 0.011). This supports the existence of an association between the clustering patterns of SMPs and SNPs.

To determine the similarity of the epigenetic/genotypic profiles, frequency estimates were calculated for SNPs and SMPs across the genome. No correlation between genotype and epigenotype was detected at this resolution (Supplemental Fig. S4). We constructed distance matrices for the 18,965 CpG SMP sites and a comparably sized subset of the 53,341 variable SNP sites. Comparisons were then made using the nonparametric Mantel test to compute Pearson product-moment correlation between the matrices (Methods). A weak positive correlation of 0.394 was observed between the matrices (α = 0.05, *P* < 0.001) (Supplemental Fig. S5). Since this correlation is low, genotype and methylation are likely to be linked, but methylation can also develop independently of genetic variation. To corroborate this, we noted a broad-range tendency for accessions clustering closely by SMP profile to show similar levels of methylation overall (Fig. 2A; Supplemental Fig. S6A). However, by ordering accessions based on genotypic information and comparing their methylation profiles, only closely related accessions share similar methylation levels (Fig. 2B; Supplemental Fig. S6B).

In summary, the methylation profiles of native accessions for mid/smaller-sized countries, e.g., the UK, Greece, Afghanistan, Cyprus, and Italy, are more likely to cluster together. These lines most likely evolved in similar environmental conditions and have similarly adapted methylation profiles. Conversely, we see accessions from geographically distant locations with comparable methylation; this may represent conserved environmental conditions that have resulted in a similar adaptive change in methylation profiles. For accessions where we have more accurate positional information for geographical origin, this association between methylation and more local adaptation (within a country) is clearer (see Supplemental Table S9; Supplemental Note S5; full passport data for the Watkins lines is available at https://www.jic.ac.uk/GERMPLASM/Cereal%20Collections%20Public%20GRU.html).

### Distinctive patterns of methylation are associated with different classes of gene function

Our analysis of the landraces clustered accessions with similar patterns of methylation into eight distinct groups (Fig. 1D). To assess if these clusters represented any functional consequences of gene methylation, genes that were methylated within each cluster were analyzed by Gene Ontology (GO) enrichment for molecular functions (topGO, *P* < 0.05) (Alexa et al. 2006). At this level of analysis, all eight clusters had distinctive profiles of enriched GO terms across multiple functional categories of genes (Supplemental Tables S10, S11). To ascertain if there were any functional consequences of gene methylation patterns within these clusters, information on differential gene expression was included in these analyses and is shown later in Supplemental Tables S24–S26.

### Tri-genome is the most stable form of methylation

We classified methylation as tri-genome (in three subgenomes), bi-genome, and uni-genome (in two or one subgenome, respectively) (Methods; Supplemental Table S12). Supplemental Table S13 details differentially and tri-genome methylated CpG, CHH, or CHG sites averaged across the accessions. The observed methylation landscape largely reflects that seen in our previous analysis (Supplemental Note S6; Gardiner et al. 2015).

**Figure 3.**
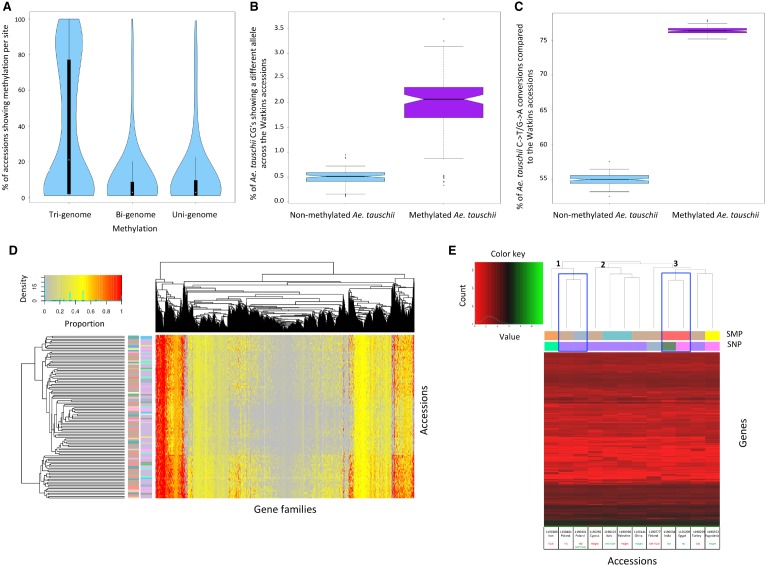
Analyzing methylation profiles across the Watkins collection. (*A*) Violin plots show the percentage of accessions showing methylation per site. Analyzed sites include Tri-genome, Bi-genome, and Uni-genome methylated sites. A comparative subset of 11,769 sites was used for each category. (*B*) Ancestral methylation associates with an increased SNP rate. The percentage of methylated versus nonmethylated *Aegilops tauschii* cytosines that show a different allele in Watkins. (*C*) Ancestral methylation demonstrates that 5-methylcytosines are preferentially deaminated to thymine. The percentage of methylated versus nonmethylated *Ae. tauschii* cytosines with a C-to-T/G-to-A transition across the Watkins collection. (*D*) Accession clustering based on the gene families targeted by methylation. Many accessions from the same geographical origin show the same gene families targeted by methylation and are, thus, clustered close to each other in the Accessions axis (vertical dendrogram). Alongside the vertical dendrogram, the two columns of row barcodes (*left* and *right*) correspond to the SMP clusters in Figure 1D and SNP clusters in Figure 1B, respectively. (*E*) Clustering of the 12 accessions subjected to RNA-seq using average gene expression across the replicates for genes showing differential expression between at least two lines (after log_2_ transformation). The horizontal dendrogram has its three main clades labeled 1, 2, and 3. *Below* the horizontal dendrogram, the two barcode rows (*top* and *bottom*) correspond to the SMP and SNP clusters in Figure 1, D and B, respectively. Accessions are labeled by line number, country of origin, and phenotype, i.e., TGW (thousand grain weight), HD (heading date), GW (grain width), or Height, with maximum values in green and minimum values in red. Accessions showing the phenotypic tails for heading date that are of interest within this study are highlighted on the horizontal dendrogram with a blue box.

To assess the relative stability of uni-, bi-, and tri-genome methylation across the Watkins collection, we identified positions that were uni-, bi-, or tri-genome methylated in one or more of the accessions. From these positions, we selected all sites that had mapping coverage ≥10× in all accessions, independent of their methylation status. Figure 3A highlights a median of 20.95% of accessions showing conserved tri-genome methylation compared to only 2.85% of accessions with conserved uni- or bi-genome methylation. Furthermore, 14.3% of tri-genome sites were methylated in the majority of accessions (≥90%), whereas, on average, only 1.08% of uni- and bi-genome sites showed methylation conservation on this scale (Supplemental Table S14). Tri-genome methylation is significantly more conserved across the accessions compared to uni- and bi-genome methylation, respectively (bi-genome *t* = 74.66, df = 16,508, *P*-value < 2.2 × 10^−16^; uni-genome *t* = 67.56, df = 17,848, *P*-value <2.2 × 10^−16^), and this tri-genome methylation is evenly distributed across the genome (Methods; Supplemental Fig. S7, track 1, 5, and 9). Gene Ontology enrichments, for genes associated with the most stable subset of tri-genome methylation (in ≥90% of accessions), included core biological activities within the plant, such as phosphorylation, intracellular transport, transcription regulation, oxidation-reduction, proteolysis, and methylation (Supplemental Table S15).

### Genome-specific methylation associates with homoeologous SNPs

We analyzed methylation variation where all accessions contained the same sequence. Looking at the cytosine residue sites that were mapped to ≥10× in all of the accessions, most (89.0%) shared the same genetic sequence, i.e., cytosine CpG/CHG/CHH context, and were therefore used to identify 359,500 SMPs. Methylation is a source of variation in the absence of genetic variation; however, we also assessed the impact of SNPs on methylation. Across all the accessions, at cytosine sites showing tri-genome methylation, the average percentage of sites where a SNP altered the cytosine context between the subgenomes of wheat is unsurprisingly low (3.50%)—methylation levels at these positions are conserved between the genomes. Conversely, at uni-genome methylation sites, it is more common to see a homoeologous SNP between the subgenomes of wheat that differentiates the methylated genome from the other two subgenomes (at 65.1% of uni-genome methylated sites). This SNP typically infers a CpG site from a non-CpG site (96% of the time).

### Ancestral methylation can be hard-coded as SNPs

As per the methodology for the 104 Watkins accessions and Chinese Spring, we generated genotype and methylation information for the subgenome D ancestor (*Aegilops tauschii* accession AL8/78) to allow comparison with Watkins accessions. We observe that ancestral methylation significantly increases the chance of encountering a different allele in hexaploid bread wheat by approximately fourfold (*t* = −30.42, df = 103, *P*-value <2.2 × 10^−16^). It shows a predominance for C-to-T/G-to-A transitions that is also statistically significant (*t* = −283.7129, df = 103, *P*-value <2.2 × 10^−16^) (Supplemental Note S7A; Fig. 3B,C). These C-to-T/G-to-A transitions are characteristic of the deamination of a methylated cytosine. This apparent preferential deamination of 5-methylcytosine to thymine has been observed in other organisms and in *Arabidopsis*, where it contributed to bias in spontaneous nucleotide mutation (Duncan and Miller 1980; Ossowski et al. 2010). Furthermore, there was high methylation stability in wheat where most methylation was conserved between *Ae. tauschii* and subgenome D (83.7%). There was a low level of methylation gain in subgenome D compared to *Ae. tauschii* (3.1%) (Supplemental Note S7B).

### Differentially methylated region profiles reflect SMP profiles

Gene expression changes are often associated with methylated regions rather than single methylated nucleotides. Using nonoverlapping 100-bp windows across the genome, differentially methylated regions (DMRs) were identified in the CpG, CHG, and CHH contexts between each accession and Chinese Spring (Methods; Eichten et al. 2016). Per accession, on average 58.7 CpG (range 37–89), 13.4 CHG (range 8–23), and 20.1 CHH DMRs (range 0–168) were identified (Supplemental Table S16). In total, 2356 DMRs of 100 bp were identified across the accessions compared to Chinese Spring (491 CpG, 96 CHG, and 1769 CHH DMRs). Of these, 1901 DMRs associated with 1744 genes and 71 DMRs were located in promoter regions associated with 64 genes. For all 2356 DMR sites, similarly to the analysis for SMP sites, the percentage difference in methylation per accession compared to Chinese Spring was used to cluster the accessions (Supplemental Fig. S8). A strong positive correlation exists between the clustering of CpG SMPs and DMRs and similar trends are observed with DMRs as was seen for SMPs (Supplemental Note S8).

For all accessions, we summarized the number of differentially methylated genes (DMGs) by methylation context, i.e., genes with a DMR compared to our reference Chinese Spring (Supplemental Fig. S9A). Variation between accessions was highest for CHH DMGs, while the number of genes showing differential methylation in the CpG and CHG contexts is more stable across accessions. CHH variability may reflect the reported dynamic nature of CHH methylation during plant development (Bouyer et al. 2017). There was no evidence of bias in the methylation contexts CpG/CHG/CHH between the wheat A, B, and D subgenomes (Supplemental Figs. S9B–D, S10A–C).

### Accessions cluster by preferentially targeted genes and gene families

Accessions were clustered based on similarities in the proportion of the number of genes that are methylated in each gene family (Fig. 3D, vertical dendrogram; Supplemental Note S9). We observe inter-accession variation in gene families highly targeted for methylation. However, a number of gene families are preferentially targeted for methylation across multiple accessions with a high proportion of genes in the family methylated (Fig. 3D, horizontal dendrogram, colored red in heat map). GO enrichment analysis revealed the most common molecular functions associated with highly methylated gene families within and between accessions (Supplemental Tables S17, S18). Hexokinase activity and glucose binding were the top enriched molecular functions for highly methylated gene families conserved between accessions (Supplemental Table S18). These terms are linked to cellular glucose homeostasis and support the hypothesis that some gene families are consistently targeted by methylation across the Watkins collection.

We performed GO enrichment analyses on gene families that were less targeted by methylation within and between accessions (Supplemental Tables S19, S20). NAD binding and N-methyltransferase activity were the top enriched molecular functions for low-level methylated gene families conserved between accessions (Supplemental Table S20). Enriched GO terms for highly methylated gene families and less methylated families did not overlap, suggesting that genes of the same molecular function are either consistently methylated or nonmethylated across the accessions.

Finally, we focused on CpG methylated genes, appearing in a high, medium, or low number of accessions (Methods). Supplemental Figure S11 shows the distribution of the number of genes in the three groups: high, medium, and low; ∼35% of the 2145 CpG methylated genes were present in ≥90 accessions. Previously, we observed that few (0.5%) SMPs were methylated in at least 90% of the accessions, but this analysis considered CpG and non-CpG sites. For CpG sites, 13.9% of SMPs were methylated in ≥90 accessions. Therefore, at the gene level, we see a ∼2.5-fold increase in methylation conservation across accessions compared to SMPs (13.9%–35%). This demonstrates an increased tendency for methylation targeting the same genes across accessions even if the specific cytosine sites differ. Furthermore, the enriched molecular functions within the high, medium, and low groups were different with no overlaps (Supplemental Table S21).

### Differential methylation correlates with changes in gene expression

To test the correlation between methylation and gene expression across the Watkins collection, we performed RNA-seq analysis, using 14-day-old wheat seedlings on 12 accessions in triplicate, which represent phenotypic tails for height, heading date, thousand-grain weight, and grain width (Supplemental Table S22). We generated gene expression level estimates to allow pairwise comparisons and identify differential gene expression between the accessions. All pairwise comparisons use the three replicates per accession to ensure statistically robust calls (Methods). Across the sample-set, 105,425 wheat genes were analyzed and, comparing the 12 accessions, 16,112 were differentially expressed; 32.3% from the A-genome, 44.6% from the B-genome, and 23.1% from the D-genome (15.3% of analyzed regions) (*P*-value < 0.05).

We normalized allelic gene expression so that per site cumulative expression values for the A, B, and D subgenomes were equal to 100%. The average expression level of subgenome A across the 289 tri-genome sites associated with promoter regions was 34.22%, subgenome B 33.43%, and subgenome D 32.35%, demonstrating approximately balanced allelic expression in the subgenomes. The average expression level of the methylated genome across the 128 promoter-associated uni-genome methylation sites was 28.82% while that of the other genomes was, on average, 35.59%. Therefore, there was a decreased expression of the promoter-methylated subgenome in comparison to the other two subgenomes (*P* < 0.0001, *t* = 5.95, df = 254).

Previously, we identified DMRs across the accessions by comparing nonoverlapping 100-bp windows with Chinese Spring (Methods). Here, we focused on the 12 accessions that were analyzed by RNA-seq and implemented pairwise comparisons to identify DMRs to allow correlation with differential gene expression from the same pairwise comparison. Inter-accession pairwise comparisons yielded an average of 58.9 CpG, 11.2 CHG, and 30.0 CHH DMRs per comparison (Supplemental Table S23); 32.3% of the DMRs were associated with differentially expressed genes. This reflects a more than twofold enrichment in the proportion of genes overall that show differential gene expression. All differentially expressed genes that correlated with DMRs were subjected to the enrichment of molecular functions and biological processes using topGO (*P* < 0.05) (Supplemental Tables S24–S26; Alexa et al. 2006). DMRs that correlate with differential gene expression are more likely to be influencing this expression change, and here, CpG DMRs show enrichment for biological processes related to homeostasis and essential housekeeping. Conversely, non-CpG methylation associates with differentially expressed genes in biological processes related to stimuli response.

For genes that were both differentially expressed and methylated, there is also a bias for enriched GO terms with molecular functions relating to metal ion transportation (Supplemental Table S24). Enrichment for transporter and metal ion binding activity was seen across SMP accession clusters (Supplemental Tables S10, S11; Fig. 1D). This bias of methylation to affect gene expression in pathways related to detoxification and metal ion transportation could be an adaptive response to differences in the soil composition in the country of origin of the accession (Supplemental Table S25; Supplemental Note S10). Furthermore, the methylation and gene expression correlations fit the directionality models predicted by previous studies for methylation based on genic positon (Brenet et al. 2011; Maussion et al. 2014; Yang et al. 2014). We focused on genes showing differential expression and methylation that had a clearly defined metal ion interaction. This narrowed our analysis to: first, a sodium/hydrogen exchanger that showed up-regulated expression from a (former) Yugoslavian accession 1190352 compared to the Cypriot accession 1190292. Up-regulation of this exchanger is associated with adaptation to salt tolerance that is biologically relevant since Yugoslavia reportedly had large areas of salt-affected soils when Cyprus was, at the time, unaffected (Szabolcs 1974; Apse et al. 1999). Furthermore, leaves from the Yugoslavian accession 1190352 show significantly higher Na concentrations (average 2182.1 ppm) compared to accession 1190292 (average 1257.7 ppm; *t* = 5.013, df = 4, *P*-value = 0.0074) (Supplemental Fig. S12A; Methods). Secondly, the ATP-dependent zinc metalloprotease *ftsH 2* showed up-regulation in the Palestinian accession 1190398 compared to a number of other accessions. *ftsH* is down-regulated after exposure of plants to elevated zinc concentrations (Garcia et al. 2009). Here, the Palestinian accession 1190398 shows *ftsH 2* up-regulation coupled with a lower average leaf Zn concentration (48.63 ppm) compared to each of the three accessions—1190141-China (66.64 ppm), 1190292-Cyprus (68.55 ppm), and 1190352-Yugoslavia (75.28 ppm)—for which leaf Zn concentrations were available. The differences in zinc concentrations were not significant, however; they fit the directional model for zinc response (*t* = 1.105, df = 10, *P* = 0.2949) (Supplemental Fig. S12B; Methods).

### Early heading date associates with SMP but not SNP profiles

The average expression levels per accession (across the replicates) for the 16,112 differentially expressed genes in one or more of the pairwise comparisons were used for hierarchical clustering (Fig. 3E). The barcodes in Figure 3E allow comparison of gene expression clusters with SNP/SMP clusters from Figure 1, B and D, respectively. Accessions that cluster into the same clades by gene expression profiles also cluster closely by SNP profile; 66.6%, 100%, and 40% of accessions within each dendrogram clade labeled 1, 2, and 3, respectively, in Figure 3E, are from a single conserved SNP cluster. This is demonstrated in Figure 3E by conserved color blocks in the SNP barcode within dendrogram clades. These patterns are also apparent from correlating gene expression and SMP profiles but to a lesser extent where 33.3%, 50%, and 40% of accessions within each dendrogram clade labeled 1, 2, and 3, respectively, in Figure 3E are from a single conserved SMP cluster.

Heading date associates with a distinct clustering of accessions (Fig. 3E). The two accessions 1190209/1190034, with earlier heading dates, show the most similar gene expression profiles of all analyzed accessions. The accessions 1190481/1190181, with later heading dates, cluster together almost as closely, but importantly, they are segregated from 1190209/1190034. The two accessions with earlier heading dates cluster into the same SMP clade but different SNP clades while, conversely, the accessions with later heading dates cluster into the same SNP clade but different SMP clades. This could indicate a common role for methylation in the establishment of an early heading date that correlates with gene expression profile.

We identified differentially expressed genes between early and late heading accessions in a pairwise comparison matrix if they were conserved across all replicates; 46 annotated genes were identified (Supplemental Table S27). This includes genes previously linked to flowering time or heading date regulation, e.g., *REVEILLE 8*-like/*LHY-CCA1*-like 5 that is here down-regulated in early heading date plants (Farinas and Mas 2011). Where methylation associates with these genes, it correlates with the expected directional effect (Supplemental Note S11A). Furthermore, Supplemental Table S28 shows the most significantly enriched GO terms and associated biological processes, respectively, for the 46 differentially expressed genes (topGO, *P* < 0.05) (Alexa et al. 2006). Enriched processes are predominantly related to meristem growth, development, cell cycle process, and phase transition and therefore show biological relevance to the phenotype (Supplemental Note S11B).

### Transposable element abundance is highly variable across the Watkins collection

When using sequence capture typically a small proportion of the resultant sequencing data is off-target since it has not been specifically targeted by the probe set but has been carried through to sequencing as background noise during capture. Analysis of Chinese Spring off-target sequence data demonstrates that it is unbiased sampling of the genome, equivalent to low coverage shotgun sequencing of total wheat DNA, since proportions of TE types closely match those seen in previous shotgun sequence data (Supplemental Table S29; Supplemental Note S12A; Brenchley et al. 2012). To assess TE methylation levels for each Watkins accession, off-target sequencing data was aligned to the wheat TREP database of repeat sequences (Wicker et al. 2002). Across the Watkins collection, transposons are highly methylated compared to the enriched gene-rich regions (Supplemental Note S12B; Supplemental Table S30). This hypermethylation of repeats is consistent with other plant species and is associated with reducing transposon mobilization.

**Figure 4.**
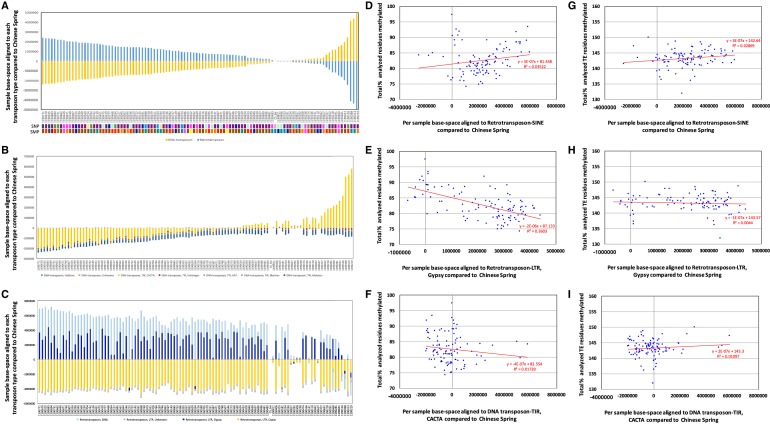
Analyzing transposable element methylation profiles across the Watkins collection. (*A*) Base-space per Watkins accession aligned to DNA-transposons and retrotransposons in comparison to Chinese Spring (Methods). (*B*) Base-space per Watkins accession aligned to DNA-transposons in comparison to Chinese Spring. (*C*) Base-space per Watkins accession aligned to retrotransposons in comparison to Chinese Spring. (*D*) Base-space per Watkins accession aligned to retrotransposon-SINE in comparison to Chinese Spring, plotted versus the total cumulative percentages of enriched cytosine residues (gene-associated) that were methylated for CpG, CHG, and CHH methylation. (*E*) as per *D* but for retrotransposon-LTR;Gypsy. (*F*) as per *D* but for DNA-transposon-TIR;CACTA. (*G*) Base-space per Watkins accession aligned to retrotransposon-SINE in comparison to Chinese Spring plotted versus the total cumulative percentages of TE-associated cytosine residues that were methylated for CpG, CHG, and CHH methylation. (*H*) as per *G* but for retrotransposon-LTR;Gypsy. (*I*) as per *G* but for DNA-transposon-TIR;CACTA.

We observed high variability across the Watkins collection in the proportions of reads aligning to each TE compared to Chinese Spring (Methods; Supplemental Note S12C; Fig. 4A–I); expansion of retrotransposons is most frequent, with 44.2% of accessions showing an increase in mapped base-space of 2% or more compared to Chinese Spring, although large expansions of the mapped base space of 8%–10% are seen in DNA transposons in a small subset of lines (Fig. 4A). TE expansions do not correlate closely with gene-associated SNP/SMP clusters or geographical clustering. It appears that expansion within the TIR;CACTA group is responsible for increasing the proportion of DNA transposons compared to Chinese Spring in a subset of Watkins accessions (Fig. 4B). This expanded group of DNA transposons showed conservation of the high methylation levels seen typically across TEs (Fig. 4I). SINE and LTR;Gypsy retrotransposons show prominent and variable expansion compared to Chinese Spring across the Watkins collection (Fig. 4C) coupled with conservation of the high methylation levels seen typically across TEs (Fig. 4G,H). These findings are consistent with previous observations that LTR retrotransposons are epigenetically controlled and a major contributor to genome size change in plants (Lee and Kim 2014).

## Discussion

Using sodium bisulfite treatment and targeted gene enrichment, we observe high epigenomic diversity in the Watkins collection. We identified three main sources of variation across wheat landraces; high transposable element variability, alongside epigenetic and genetic diversity. Although we found a general correlation between methylation patterns and genotypic variation, there was a geographical component to methylation patterns that may indicate a response to or selection by local environmental conditions. Both methylation and genotype are influenced by the geographical origin of the accession, although genotypic profiles cluster across wider geographic regions while the methylation profiles of accessions tend to cluster into more local groups. Therefore, we hypothesize that methylation acts as a fast-adaptive response to environmental stimulus. Furthermore, we show that ancestral methylation increases the chance of C-to-T or G-to-A transitions in Chinese Spring wheat that are characteristic of the deamination of a methylated cytosine and may demonstrate this transfer of methylation to SNPs (Duncan and Miller 1980; Ossowski et al. 2010). This phenomenon could be an important driver of evolutionary change.

We show that tri-genome methylation is more conserved between accessions and therefore the most stable form of methylation, while genome-specific methylation sites show enrichment for homoeologous SNPs that differentiate the genome that is methylated from the other subgenomes. This SNP typically infers a CpG site from a non-CpG site. Tri-genome methylation correlates with equal expression levels across the three subgenomes, while uni-genome methylation correlates with a significant reduction in expression of the affected subgenome compared to the other two subgenomes in promoter regions.

Watkins accessions were clustered according to methylation profiles, and the clusters show unique profiles of enriched gene function. These variations could contribute to the underlying phenotypic differences between the accessions. Using gene expression analyses, we saw conserved methylation and gene expression profiles in accessions with an early heading date, suggesting that methylation may play a role in the coordination of heading date in wheat. DMRs linked directly to gene expression show a bias for genes related to metal ion transportation that links to phenotypic change and could be part of an adaptive response that has been maintained in certain accessions due to differences in the soil composition in the country of origin of the accession.

In addition to epigenomic diversity across the Watkins collection, using Chinese Spring as a baseline, we observe the potential expansion of retrotransposons SINE and LTR;Gypsy most frequently, although some of the largest expansions are seen in a small subset of lines in DNA transposons. These expanded groups of TEs showed conservation of the high methylation levels seen across TEs.

We explore genome-wide epigenetic, alongside genotypic and TE variation across a diverse landrace cultivar collection and open up a new level of genetic variation, which can be exploited by breeders. This provides further opportunities to address important biological questions such as the interaction between epitype and genotype, the role of epigenetics in the domestication of crops, and the stability of and long-term function of methylation in a polyploid genome.

## Methods

### Design of the methylation enrichment system

The 12-Mb target sequence for this Agilent enrichment system was generated as per Supplemental Figure S1 from Olohan et al. (2018). For the capture, 99,949 120-mer RNA baits were designed. The 120-mer baits were uploaded onto Agilent's eArray (online custom microarray design tool) to allow submission for manufacture. Bait “boosting” was selected to allow excess unused design space (<1 Mb in this case) to be filled with repeat sequences of baits that are predicted to perform less efficiently, i.e., those with an above average GC content are “boosted” to ultimately gain even depth of sequence coverage across the target region.

### Preparation and mapping analysis of DNA samples

Single seedlings were examined for all 104 core lines from the A.E. Watkins bread wheat landrace collection plus the reference variety Chinese Spring (Supplemental Table S1). DNA was extracted from seedlings, fragmented, and taken through a modified sequence capture protocol to allow genetic and methylation analysis of the same enriched genomic DNA sample by splitting the sample post-capture (Supplemental Methods; Olohan et al. 2018).

The non-bisulfite-treated paired-end sequencing data sets for the accessions were mapped to the TGAC reference sequence using BWA MEM version 0.7.10 (Li and Durbin 2009). Mapping results were processed using SAMtools (Li et al. 2009); any nonuniquely mapping reads, unmapped reads, poor quality reads, and duplicate reads were removed. SNP calling was carried out using the GATK (McKenna et al. 2010) Unified genotyper (after Indel realignment), which was used with a minimum quality of 30 and filtered using standard GATK recommended parameters, a minimum coverage of 5, and homozygous SNPs only selected (alternate allele in ≥80% of sequencing reads). Homozygous SNP alleles were used to correct the TGAC reference sequence to generate an accession-specific reference sequence for each analyzed accession that was implemented for mapping the corresponding bisulfite-treated data set to using Bismark, an aligner and methylation caller designed specifically for bisulfite-treated sequence data (Krueger and Andrews 2011).

For mapping analyses using Bismark, the nondirectional nature of the library was specified, and subsequently SAMtools was used to identify and remove duplicate reads. The Bismark methylation extractor tool was used to identify all cytosine residues within the mapping and categorize the reads mapping to them as unmethylated or methylated at that position while also detailing which type of potential methylation site was present (CHH, CHG, or CpG). This output can then be used to calculate the percentage of the reads that were methylated at each cytosine residue site. Under the same rationale, differential methylation was identified between subgenomes and/or accessions at a minimum difference of 50% to ensure elimination of replicate variance and the analysis of genuine methylation changes.

### Alignment of the three subgenomes of wheat

The three subgenomes were aligned using the software NUCmer from the MUMmer package that is specialized for the alignment of incomplete genomes with large numbers of reference contigs (Kurtz et al. 2004). After the alignment, using the MUMmer package, result files were filtered to determine a one-to-one mapping of subgenome A to B and A to D, and indels were identified between the sequences. Finally, for each subgenome A cytosine/guanine position in the TGAC reference sequence, if this was in a region showing an alignment with both subgenome B and subgenome D, the corresponding position in subgenomes B and D was calculated. Indel information was used to ensure that single positions were correctly translated between aligned subgenomes even if alignments contained gaps.

### Calculation of bisulfite conversion rate

Bisulfite treatment involves converting cytosine to uracil while leaving 5-methylcytosine (5-mC) intact. Therefore, bisulfite conversion rates can be measured by mapping reads to the chloroplast genome, which is unmethylated, since we know that here all cytosines should be converted to uracil (Fojtová et al. 2001; Lister et al. 2008). While we did not enrich for chloroplast DNA, because we used total wheat DNA, a proportion of our off-target sequences mapped to the wheat chloroplast genome. The off-target DNA was mapped to an average of 66.5% of the chloroplast genome across all accessions to 406× per accession (Supplemental Table S3).

### Setting thresholds for calling methylation

To discriminate methylated CpG, CHG, and CHH sites from nonmethylated residues, we used standard thresholds for each category based on previously published methodologies (Song and Chen 2015) that take into account the tendency for a high-level average CpG methylation and low-level average non-CpG methylation in gene-rich enriched data sets that was reflected in this study (Supplemental Fig. S2). Thresholds of ≥75% methylation were used to categorize the CpG data as methylated and thresholds of ≥10% methylation to categorize the CHG and CHH sites as methylated. However, this means that intermediate-level methylation, which is likely to be associated with tissue-specific regulation, was not fully described and is beyond the scope of this study.

### Implementation of methylKit

The software methylKit (Akalin et al. 2012) was implemented to identify regions of differential methylation using positional information for each cytosine plus the number of reads hitting it per subgenome and each read's methylation status. Pairwise comparisons were used, and as such, Fisher's exact test was used to discriminate statistically significant differences (*q* < 0.01 and methylation difference of ≥50%). First, methylKit was implemented to define differential methylation between the subgenomes of wheat, within each accession, in regions where the three subgenomes could be aligned. Differences were recorded at single cytosine residues between one genome and the other two (uni-genome methylation) and vice versa (bi-genome methylation). Finally, after identification of DMRs, methylKit was implemented with pairwise comparisons of accessions to define DMRs between the two accessions (*q* < 0.01 and methylation difference of ≥50%).

### Identification of SMPs and SNPs

SMPs were identified by looking for sites that were covered by at least 10 reads and were either called methylated (denoted as 100%), using our standard thresholds, or showed no methylation (<1%), which we defined as an unmethylated site (denoted as 0%). Any other sites with no coverage were listed as missing or, with intermediate methylation levels, were listed as heterozygous (denoted as 50%). A general SMP was defined as any site with sufficient coverage for all of the analyzed accessions where at least two accessions were called methylated, at least two accessions were called unmethylated, and where all accessions contained the same sequence as the Chinese Spring reference genome, i.e., no SNP altering the cytosine context between CpG, CHH, and CHG. An individual accession SMP was defined as any site from the general SMP list where the accession was denoted as being 100% methylated. SNPs were called as previously detailed. For clustering analyses, 53,341 SNP sites were identified across the 105 accessions, where all accessions showed mapping coverage at ≥5× and ≥1 accession was found to have a SNP, i.e., variable sites.

### Dendrogram construction

If dendrograms accompany heat maps, then they have been produced using the R function heatmap.2 (R Core Team 2013) from the gplots package with the default clustering parameters (complete linkage method with Euclidean distance measure). The dendrograms that lack heat maps were produced by first generating a distance matrix with R's *dist* function and passing this matrix to the *hclust* function, both with their default parameters. Furthermore, the R package pvclust was implemented to generate the dendrograms as detailed, however with the additional computation of *P*-values for clusters; AU (approximately unbiased) *P*-values were computed by multiscale bootstrap resampling (minimum bootstrap number of 10,000).

The SNP-based tree (Fig. 1B) was cut into nine groups using the R package cutree, and the SMP-based tree was similarly cut into eight main groups to allow direct comparison of SMP and SNP groups (Supplemental Methods).

We repeated the clustering analysis on the 53,341 SNPs using a maximum likelihood (ML) modeling approach (Supplemental Fig. S1; Supplemental Methods).

### Distance matrix construction and comparison

Distance matrices were constructed individually for the 18,965 CpG SMP sites and a subset of 17,780 of the 53,341 variable SNP sites using the R function *dist* (R Core Team 2013). These distance matrices were then compared using the mantel.randtest function in R to perform the Mantel test with 999 permutations. Distance matrices were also converted to heat maps using the R function heatmap.2 without dendrogram construction or clustering.

### Construction of pseudochromosomes

We used 21 wheat chromosomal pseudomolecules that were created by organizing and concatenating the TGAC genome assemblies onto POPSEQ-based pseudomolecules using the software NUCmer (Kurtz et al. 2004; Chapman et al. 2015; Gardiner et al. 2016). After the alignment, using the MUMmer package, result files were filtered to determine a one-to-one mapping of TGAC subgenome A to POPSEQ-based subgenome A, B to B, and D to D. Relative positions for the TGAC contigs along the POPSEQ chromosomal pseudomolecules could then be used to order them into our chromosomal pseudomolecules.

### Identification of DMRs

We focused on the 853,932 cytosine residue sites that were mapped to a minimum of 10× in all of the 104 accessions plus Chinese Spring. Using these sites, DNA methylation for the three contexts (CpG/CHG/CHH) was averaged independently across nonoverlapping 100-bp windows. A window was only considered if a minimum of five cytosines were included in the region. This yielded 2277 CpG, 3721 CHG, and 44,371 CHH windows for analysis. For every window, each accession was compared individually with Chinese Spring (see implementation of methylKit) to identify DMRs. For CpG and CHG sites, a DMR was called if a region showed a difference in methylation of at least 50% (*q*-value < 0.01). However, for CHH sites, a DMR was called if a difference of at least 15% was observed (with one accession showing “low” or ≤5% methylation and a *q*-value of <0.01).

### Association between SMP and SNP clusters and enrichment for molecular functions

Gene set enrichment analysis (GSEA) was performed on the eight main SMP clusters that are shown in Figure 1D using the R package topGO (Supplemental Methods; Alexa et al. 2006).

The arrangement of accessions in the merged SMP (Fig. 1D) and SNP (Fig. 1B) trees in Figure 1E were assessed for randomness using the randomness test (Supplemental Methods).

### Enrichment analysis for the methylated genes in three groups (high, medium, and low)

We calculated the number of genes targeted by methylation by tallying all CpG methylated genes with copy number ≥1 that are present in at least one accession. These genes were categorized into three groups, namely, those with (1) high representation in most accessions (appear in 90 or more accessions, i.e., high group), (2) medium representation across accessions (appear in 40–90 accessions, medium group), and (3) low representation across accessions (appear in less than 40 accessions, low group). All genes in these groups were those targeted by CpG methylation. The genes in each group were collated as gene sets and analyzed for the enrichment of significant molecular functions and corresponding overrepresented GO terms. This analysis aids assessing any differences in the enrichment of gene sets from the groups, thereby enabling inference into the gene methylation profiles within each group. This also shines light into what enriched molecular functions can be associated with any phenotypic differences as a result of the underlying methylation profiles of targeted genes within each group.

### Data filtering and inference: association between methylation and gene families

To investigate the association of methylation with gene families, we extracted family clusters (Supplemental Methods). In all accessions, gene families with an average of ≥25% representation of genes targeted by methylation were considered for GSEA using a targeted and nontargeted approach. The heatmap.2 function (gplots R package) was used to generate dendrograms. A distance matrix was first generated using the *dist* function and then passed to the *hclust* function for hierarchical clustering.

### Generation of RNA-seq data for 12 accessions for differential gene expression analysis

Three seedlings were examined for all 12 lines from the A.E. Watkins bread wheat landrace collection (Supplemental Table S22). Total RNA was extracted from the areal tissue of these 14-day-old wheat seedlings grown at a constant 24°C under long days using Qiagen RNeasy plant mini-kits. Library preparation and RNA-seq was performed by the Earlham Institute platforms and pipelines using the HiSeq 4000. Raw sequencing reads were trimmed for adapter sequence and also for regions where the average quality per base dropped below 15 (Trimmomatic version 0.32) (Bolger et al. 2014). After trimming, reads below 40 bp in length were eliminated from the data set. Trimmed reads for each sample were individually aligned to the Chinese Spring wheat reference genome using the splice-aware aligner HISAT2 (Pertea et al. 2016). Uniquely mapped reads were selected and duplicate reads filtered out to yield a “final mapped reads set” per sample. The program StringTie was implemented to assemble transcripts, guided by the read alignments to the reference genomes, and to estimate their abundances for each sample. Transcript assemblies or gene structure annotations could then be collated across the samples to form an analysis-specific gene annotation summary, i.e., a comprehensive list of all genes showing expression in at least one sample in the study. StringTie was then used to calculate gene and transcript abundances for each sample across the analysis-specific annotated genes. Finally, Ballgown allowed visualization of results and identification of differential expression between accessions (Pertea et al. 2016). Differential gene expression was called between accessions at a threshold of *P* < 0.05, taking into account all three replicate samples per accession to confirm a call.

### Ionomics

Twelve plants for each Watkins accession were grown in groups of four, and these were pooled (to give three replicate sets of plants, each replicate originating from one “cigar roll”). All plants were grown on a standard nutrient solution (Bai et al. 2013). For the three replicates of each Watkins accession, elemental analysis was performed on an ICP-MS (inductively coupled plasma mass spectrometry), and normalized concentrations of the samples were obtained as per the methods from Hosmani et al. (2013). Twenty elements (Li, B, Na, Mg, P, S, K, Ca, Mn, Fe, Co, Ni, Cu, Zn, As, Se, Rb, Sr, Mo, and Cd) were monitored, of which Na and Zn were used here.

### Transposon analysis

For each Watkins accession, the cumulative coverage from the alignment of off-target reads to the TREP database was normalized to 50,000,000 bp to match Chinese Spring most closely. Chinese Spring was also normalized to 50,000,000 bp. For each transposon type, the base-space alignment coverage for Chinese Spring was subtracted from the corresponding Watkins accession value to yield a comparative value, i.e., negative meaning the transposon showed higher coverage in Chinese Spring and positive meaning the Watkins accession showed higher coverage. These values were used to construct Figure 4A–C. Note that for Figure 4A, since only two transposon type categories are compared, an increase in one type compared to Chinese Spring is coupled with a proportional decrease in the other group, and as such, this plot shows an apparent symmetry in the increase/decrease of TEs.

## Data access

DNA sequence reads from this study have been submitted to the European Nucleotide Archive (ENA; http://www.ebi.ac.uk/ena/) under accession number PRJEB23320. The 12-Mbp sequence capture probe set is available in Supplemental File S1.

## Supplementary Material

Supplemental Material
