# Supplementary material for: Hidden variation in polyploid wheat drives local adaptation
Source: Genome Res. 2018 Sep;28(9):1319–32. doi: 10.1101/gr.233551.117 (PMC6120627; doi:10.1101/gr.233551.117)
Supplement: Supplemental Material [file supp_28_9_1319__index.html]

Hidden variation in polyploid wheat drives local adaptation — Supplemental Material 

# Hidden variation in polyploid wheat drives local adaptation

## Supplemental Material

- Supplemental\_Material.pdf
- Supplemental\_File\_S1.txt
