# Supplementary material for: Hidden variation in polyploid wheat drives local adaptation
Source: Genome Res. 2018 Sep;28(9):1319–32. doi: 10.1101/gr.233551.117 (PMC6120627; doi:10.1101/gr.233551.117)
Supplement: Supplemental Material [file supp_gr.233551.117_Supplemental_Material.pdf]

Supplemental Material

Supplemental Methods

Preparation of DNA samples for sequencing
Dendrogram cutting to define groups
Maximum Likelihood method for clustering
GO enrichment for molecular functions.
Randomness test
Association between methylation and gene families

Supplemental Notes

Supplemental note S1-Supplemental note S12

Supplemental Figures

Supplemental Figure S1-Supplemental Figure S12

Supplemental Tables

Supplemental Table S1-Supplemental Table S30

Supplemental References

Supplemental Methods

Preparation of DNA samples for sequencing

Total genomic DNA was extracted from the areal tissue of these 14-day-old wheat seedlings grown at a constant 24°C under long days using Qiagen DNeasy plant mini kits. 3µg of each sample was sheared for 22 cycles of 30s on, 30s off, using a Bioruptor Pico (Diagenode) and 0.65ml Bioruptor tubes. Fragmented DNA was purified using 1.8 × Agencourt AMPure XP beads (Beckman Coulter) and then used as input material for preparation of libraries according to Agilent's SureSelect^{XT} Methyl-Seq Protocol Version C.0, January 2015. The pre-capture libraries were quantified by Qubit double-stranded DNA high sensitivity assay (Thermo Fisher Scientific) and the size distribution assessed by analysis on a Fragment Analyser (Advanced Analytical Technologies) using a high sensitivity NGS Kit. Each library was then enriched using the 12 Mb custom SureSelect RNA oligomer baits with use of a modified sequence capture protocol to allow genetic and methylation analysis of the same enriched genomic DNA sample by splitting the sample post-capture (Olohan et al 2018). For this, hybridization set-up and post-capture washing were carried out in batches of 48 using a Tecan Freedom EVO NGS Workstation. Enriched DNA was eluted from the Streptavidin beads with 27µl of Elution Solution and then neutralised with an equal volume of Neutralisation Solution prior to purification using 1.8 × Agencourt AMPure XP beads. At this point the enriched purified DNA was divided at a ratio of approximately 3:1. ¾ of the DNA was bisulfite treated using a Zymo Research EZ-96 DNA Methylation Gold Kit (deep well format) according to the manufacturer's instructions, but with double elution from the Binding plate using 16µl of M-Elution Buffer each time. The bisulfite-converted DNA and the remaining ¼ of untreated enriched DNA were amplified in parallel as described by Olohan et al 2018 but using 10 PCR cycles for the final indexing amplification. Following purification and QC, final libraries were pooled in equimolar amounts (genetic and methylation analysis) and sequencing was carried out on an Illumina HiSeq 4000, with version 1 chemistry, generating 2 × 150bp paired-end reads.

Dendrogram cutting to define groups

The SNP based tree (Figure 1b) was cut into 9 groups using the R package cutree. The first group, consisting of Chinese Spring only is a result of this accession being used as the reference genome and as such was disregarded from the analysis. The remaining 8 clusters represent, moving down the dendrogram, the lowest cut where we can maximize group number for analysis but the majority of groups still include enough accessions to be informative with AU p-values ≥70% i.e. lowest cut where at that point and above >50% of cluster groups show >5 members. The SMP based tree was similarly cut into the 8 main groups to allow direct comparison of SMP and SNP groups.

Maximum Likelihood method for clustering

We repeated the clustering analysis on the 53,341 SNPs using a maximum likelihood (ML) modelling approach (Supplemental Figure S1a). To most closely represent the approach that we took for hierarchical clustering we trialled 1000× repeated random haplotype sampling (RRHS), which allows the integration of information from all alleles at heterozygous sites into phylogenetic tree estimation with maximum-likelihood (ML) methods. For this analysis, we had to convert SNPs into a binary call format rather than using raw allele frequencies (Steinig et al 2016; Leache et al 2015; Lischer et al 2013). We then used information from the 1000 RRHS phylogenetic

trees to construct a consensus network for the SNPs (Supplemental Figure S1b and S1c). The edge thickness represents the support values (i.e. fraction of trees in which the edge was the chosen in minimum spanning tree representation with phylogenetic distances inferred from the ML trees).

GO enrichment for molecular functions.

Gene set enrichment analysis (GSEA) was performed on the 8 main SMP clusters that are shown in (Figure 1d) using the R package topGO (Alexa et al 2006). The gene sets in each of the clusters were collated from all accessions within each cluster and enriched for molecular functions using gene ontology, GO. TopGO integrates the GO topology in the scores and identifies over-represented terms globally. For the enrichment analysis, we used genes from CpG DMRs in the 104 accessions referenced to Chinese Spring (with an absolute methylation difference of ≥50% and a minimum of 5 cytosines).

Randomness test

The randomness test evaluates the association between the methylation (SMPs) and genetic (SNPs) components. We used the non-parametric one-sample runs test (Wald and Wolfowitz 1940) to assess the randomness of accession membership in the coloured clusters (Figure 1e). By maintaining the ordering of accessions in the SNP dendrogram (Figure 1e), the 8 SMP clusters shown in distinct colours were assigned to different categories and the neighbouring accessions in the tree were indexed as dichotomous variables (1 for the same cluster membership, and 0 otherwise). The two classes had large accessions with 61 (ones) and 43 (zeros); thereby, justifying the large-sample test approximation to the standard normal distribution, $N(0,1)$.

Association between methylation and gene families

To investigate the association of methylation with gene families we extracted family clusters with at least 2 members from bread wheat. Wheat gene families were inferred using the OrthoFinder software (Emms and Kelly 2015). We used the default parameters and recommendations given in the OrthoFinder manual. To improve the taxonomic resolution of the resulting gene families we included the comprehensive set of plant proteomes from Phytozome version 11 (Goodstein et al 2012) and the recently published barley gene catalogue (Mascher et al 2017). Protein sequences of the clustered species were additionally screened for domain architectures with HMMER3 (PMID: 20180275) against the PFAM domain database (version 30) using the cut_ga option (Finn et al 2016). The resulting domain matches were filtered using the multi-objective optimization approach implemented in DAMA (Bernardes et al 2016). As protein catalogues from genome-wide predictions contain sequences derived from transposons and gene-fusion artefacts, the top orthogroups with the most members frequently are comprised of a mixture of gene families. To eliminate these from our analyses we have manually inspected the PFAM domain profiles of the clusters and finally excluded orthogroups with more than 40 distinct PFAM domains. The resulting set of 12,323 orthogroups was used as bona fide gene (sub) families comprising a total of 164,756 loci.

In all accessions, gene families without any genes targeted by methylation were excluded from further analysis. There was a large variation in the size of targeted gene families and the number of genes within each gene family. Genes from targeted gene families (excluding larger-sized families, ≥40) were included in the analysis. The number of filtered genes was scaled by the size of the corresponding gene families since variations in the

size of gene families might influence the results. This results in a proportional representation for targeted genes within each gene family. The use of proportions evens out any bias in the representation of targeted genes resulting from the larger-sized gene families. In all accessions, gene families with an average of ≥25% representation of genes targeted by methylation were considered for GSEA using a targeted and non-targeted approach.

Supplemental Notes

Supplemental note S1: Analysis targets and parameters

a) In a previous study, we tested the performance of our experimental pipeline, and we saw high reproducibility between biological replicates (<0.09% of residues showing differential methylation) (Gardiner et al 2015). As such, for this study, DNA from single seedlings was examined for the 104 lines from the Watkins landrace collection plus the reference variety Chinese Spring.

b) Using paired-end Illumina libraries, we extend sequencing coverage out from the capture baits, allowing us to score methylation across an average of 48MB of the genome at ≥10X coverage per sub-genome, translating into an average of 10.9M cytosines per accession (Supplemental Table S2). For Chinese Spring this covered transcribed (43.9%) and promoter regions across the genome (79,623 genes including; 138,040 exons and 121,240 introns along with, 26,828 promoter regions, defined as up to 2000bp upstream of the 5'UTR) providing a comprehensive survey of genome methylation. Using the software NUCmer (Kurtz et al 2004) we defined homoeologous gene triplets within the wheat genome allowing us to accurately compare methylation across the 3 genomes at an average of 1.85M cytosines per accession in our data set at ≥10X coverage.

c) To assess the bisulfite conversion efficiency for each of the accessions, reads were mapped to the non-methylated chloroplast genome; across all accessions, on average 98.7% of cytosine bases were successfully bisulfite converted (Supplemental Table S3). This value is within the limits determined for successful bisulfite conversion in previous studies (Genereux et al 2008).

Supplemental note S2: Genetic variation across the Watkins collection clusters geographically

Additional clustering models were trialled that supported the genotype-based population structure we observed. We repeated the clustering analysis on the 53,341 SNPs using a maximum likelihood (ML) modelling approach (Methods). The resultant phylogenetic tree (Supplemental Figure S1a) has low support for the backbone clades however; the fine-scale groups that were identified within it are similar to the hierarchical clustering groups that we identified previously. This can be observed in Supplemental Figure S1a where we colour coded accessions in the ML tree according to their previously defined hierarchical clusters (Figure 1b). The conserved colour blocks across the phylogenetic tree denote accessions that are closely related using the ML model being also from the same hierarchical cluster.

We then used the ML phylogenetic tree information to construct a consensus network for the SNPs (Methods; Supplemental Figure S1b and S1c). The network in Supplemental Figure S1b confirms our previous prediction of two main ancestral groups with a clear breakaway group to the left that appears to be made up of accessions that are largely of Asian origin; to clarify this in Supplemental Figure S1c we colour code accessions according to continent of origin rather than the cluster defined from hierarchical clustering and here the division of the accessions into two main groups is clear; Asian and European/Other.

In previous analyses of the Watkins collection-using array SNP data, it was noted that European accessions clustered away from Asian and Middle Eastern accessions (Wingen et al 2014; Winfield et al 2017). This is seen

here within group 1 where Iranian accessions and those from Sub-Saharan Africa that border the Middle East, sub-cluster together (group 1a) away from European accessions. Winfield et al also saw accessions from southern USSR clustering with Middle Eastern (Iran)/Asian accessions while accessions from northern USSR tended to cluster with Eastern European accessions. We also observe both of these tendencies here.

Supplemental note S3: Global methylation patterns match other plant species

In rice, the percentages of methylated cytosines at CpG, CHG and CHH sites were 54.7%, 37.3% and 12.0% respectively. The corresponding average methylation levels in the three contexts were 44.5%, 24.1% and 4.7% (Li et al 2012). In a previous analysis of Chinese Spring wheat (6 Mb capture) we observed similar methylation patterns to that in rice; however, CHG methylation levels were more similar to CHH levels likely due to the study's focus on gene body regions that typically show enrichment for CpG methylation (Gardiner et al 2015). With the 12 Mb capture used here, for Chinese Spring, we see 71.4%, 24.2% and 1.8% of cytosines were methylated across CpG, CHG and CHH sites respectively and the average methylation levels were 66.0%, 20.9% and 1.77% (Supplemental Figure S2a). We confirm high-level CpG methylation and low-level CHH methylation in Chinese Spring. Notably, CHG methylation levels are elevated compared to those seen with the 6 Mb capture since the 12Mb capture does not bias towards gene body regions to the same extent (43.9% of analyzed residues here versus ~80% previously). Furthermore, by averaging the percentages of methylated cytosines across the 105 accessions we found 61.5%, 19.4% and 1.69% methylated across CpG, CHG and CHH sites respectively and the average methylation levels were 52.9%, 12.9% and 2.09% (Supplemental Table S5).

To discriminate methylated CpG, CHG and CHH sites from non-methylated residues we used standard thresholds based on previously published methodologies (Gardiner et al 2015) (Methods). This resulted in 18.5% of residues being called methylated in Chinese Spring (15.9% across all accessions) with most CpG sites (48%) closely followed by CHG sites (33%) and CHH (19%) sites (Supplemental Figure S2b). In general, most methylated residues were in non-transcribed regions (54.7% CpG, 75.7% CHG and 62.6% CHH sites) (Supplemental Figure S2c). There was a bias for CpG methylation in transcribed regions with 63.5% of methylated sites in the CpG context, 19.3% were CHG sites and 17.2% CHH sites. However, the prevalence of non-CpG methylation increases in non-transcribed regions where 41.2% of the methylated sites were CpG, 39.9% in CHG and 18.9% in the CHH context (Supplemental Figure S2d). These figures closely reflect those seen in our previous smaller scale survey of methylation in Chinese Spring wheat (Gardiner et al 2015). The distribution of methylated sites is relatively consistent across the genome with a slight bias for CpG and CHG methylation near centromeric regions (Supplemental Figure S2e).

Supplemental note S4: CHG and CHH methylation can be categorized as non-CpG for global analyses

We focused on SMP sites and performed pair-wise associations between methylation contexts for; CpG and CHG, CpG and CHH, CpG and non-CpG and CHG and CHH (Supplemental Figure S3a, c, d and b respectively). A strong positive association was observed between methylation in the CHG and CHH contexts ($p<2.2e-16$, $R^2=0.985$) which supports the pooling of these methylation types into a single non-CpG category. Pair-wise associations between the other contexts were not significant (all $p>0.05$ and $R^2<0.005$).

Supplemental note S5: Methylation association with local adaptation is clearer for accessions with accurate positional information for geographical origin

For example, 15 accessions have India as their country of origin, however, positional information shows that 7 of them originated in Pakistan and 3 at its border with Afghanistan (1190246, 1190433 and 1190707). Consequently, these 3 accessions show more linkage with other accessions that originate from Afghanistan. Conversely, we see accessions from geographically distant locations with comparable methylation profiles. This may represent conserved environmental conditions that have resulted in a similar adaptive change in methylation profiles e.g. accessions from China are seen in group 2c with a large number of accessions from Western Europe (Figure 1c, 1d, Supplemental Table S8). Considering climate, this observation is unsurprising since the Chinese accessions in group 2c, for which accurate geographical origins are available, are associated with the east coast of China where a mild temperate climate exists-similar to that of much of Western Europe.

Supplemental note S6: Uni-, Bi- and Tri-genome methylation analysis

For the analysis of tri-, bi- and uni-genome methylation, our focus is on sites where all three genomes align and each contains a cytosine residue that was mapped to ≥10X. This leaves a subset of cytosine residues that we are unable to incorporate in this sub-analysis including; 156,933 positions (18.9% methylated) where two sub-genomes have cytosine residues with the remaining sub-genome having an adenine/thymine residue at that position and 166,758 positions (24.2% methylated) where only one sub-genome has a cytosine residue while the remaining two sub-genomes show adenine/thymine residues.

Most sites show tri-genome methylation (56.2%) with 22.5% of sites showing uni-genome and 21.3% of sites showing bi-genome methylation on average across the accession set (Supplemental Table S13). The A, B and D sub-genomes show uni-genome methylation in similar proportions and methylation levels in the bi-genome group were also conserved across the genome pairs and accessions. For uni-, bi- and tri-genome methylation we see a drop in CpG and an increase in non-CpG methylation in non-transcribed regions compared to transcribed regions, this is consistent with other plant genomes. In transcribed regions, the bi- and tri-genome methylated residues are almost exclusively CpG sites (on average 98.6% CpG, 1.2% CHG and 0.2% CHH sites). This differs from the observation for uni- genome associations where CHG methylation is slightly elevated (on average 94.6% CpG, 5.1% CHG and 0.3% CHH sites). It is possible that non-CpG methylation in transcribed regions associates with pseudo genes in wheat, however, we only see this association for uni-genome but not bi-genome methylation.

Supplemental note S7: Ancestral methylation may become hard-coded as SNPs

a) We assessed cytosine residues across 1,236,557 sites where we see a cytosine residue with both genotype and methylation information in *Ae. tauschii* and we have the genotype and/or methylation information in Chinese Spring for sub-genome D at the same position. Firstly, we focused on sites with methylation in *Ae. tauschii* at ≥10% and this encompassed 10.4% of the 1,236,557 sites. 0.31% of these residues changed from a methylated cytosine in *Ae. tauschii* to a predominantly different allele in Chinese Spring wheat with a bias for C-to-T and G-to-A transitions present in 80.0% of cases. Secondly, we focused on sites where no methylation was observed in *Ae. tauschii* that included 89.6% of the 1,236,557 sites that were less than 10% methylated. Only 0.07% of these residues were altered from a cytosine in *Ae. tauschii* to a different allele in Chinese Spring wheat. There was a reduced bias for C-to-T and G-to-A transitions within this residue subset that were seen in

only 54.1% of cases. Ancestral methylation increases the chance of encountering a different allele in Chinese Spring by almost 5-fold and it also shows a bias for C-to-T or G-to-A transitions.

We next assessed cytosine residues across 11,815,542 sites where we see a cytosine residue with methylation information in *Ae. tauschii* and we have genotype and/or methylation information in at least one Watkins accession at the same position. Of the 11,815,542 sites 2,534,824 were methylated in *Ae. tauschii* at ≥10%. This encompassed on average 3,475,713 sites per accession for comparison (464,023 methylated and 3,011,690 non-methylated in *Ae tauschii*). Across the Watkins collection on average, 1.96% of conserved residues per accession changed from a methylated cytosine in *Ae. tauschii* to a different allele in the Watkins with a bias for C-to-T and G-to-A transitions that were seen in 76.36% of cases. In contrast to this at non-methylated *Ae. tauschii* C sites (9,280,717 sites) only 0.50% of residues changed to a different allele in the Watkins with a reduced bias for C-to-T and G-to-A transitions seen in 54.94% of cases. This reflects the analysis using Chinese Spring, however a higher SNP rate in general is observed using this diverse bread wheat collection. Moreover, using per-sample paired percentages of SNP rate and CT/GA transition rate from methylated versus non-methylated *Ae. tauschii* sites we observe a significant increase in SNP rate in general if the ancestral *Ae tauschii* showed methylation (paired t test, $t=-30.4174$, $df=103$, $p\text{-value}<2.2e-16$) and a further significant bias for C-to-T and G-to-A transitions (paired t test, $t=-283.7129$, $df=103$, $p\text{-value}<2.2e-16$).

b) We can assess methylation stability in wheat using the 1,236,557 cytosine sites, which contain residues that show methylation information for *Ae. tauschii* and Chinese Spring's D sub-genome. Looking at the 10.4% of methylated sites in *Ae. tauschii*, if these cytosine residues were conserved in Chinese Spring, a high level of methylation (83.7%) was conserved between *Ae. tauschii* and sub-genome D. This is highly similar to the 13.6% difference that was observed previously between sub-genome D methylation and *Ae. tauschii* (Gardiner et al 2015). Furthermore, looking at the 89.6% of non-methylated cytosine residues in *Ae. tauschii*, for the cytosine residues that were conserved in Chinese Spring, we observed a relatively low level of methylation gain in sub-genome D compared to *Ae. tauschii* (3.1%) i.e. a high level of conservation of non-methylated status (97.0%).

Supplemental note S8: Differentially methylated region (DMR) profiles reflect SMP profiles

For each of the 2,356 DMRs, the percentage difference in methylation per accession when compared to Chinese Spring was used to cluster accessions and assess diversity across the 104 accessions (Supplemental Figure S8). Hierarchical clustering of accessions was used to order accessions based on similarity, however, dendrograms were not constructed due to the low number of regions under analysis. Based on the DMR locations (vertical axis of Supplemental Figure S8) a larger proportion of non-CpG DMRs are located in promoter and non-transcribed regions (53.1% CHG, 22.6% CHH compared to 15.5% CpG sites), supporting our previously observed SMP bias for non-CpG methylation in non-transcribed regions. Non-CpG DMRs, in particular CHH DMRs, tend to be rare variants that are each in a relatively low number of accessions (Supplemental Figure S8b and S8c). Most of this methylation change is low-level or potentially tissue-specific so its detection may be limited in this investigation. However, a higher degree of inter-accession methylation variation is observed at CpG DMRs where variants are typically more common across the population (Supplemental Figure S8a).

We compared distance matrices for the 18,965 CpG SMP sites and the 491 variable CpG-DMRs using the non-parametric Mantel test to compute Pearson product-moment correlation between the matrices (Methods). A

strong positive correlation of 0.612 was observed between the matrices (alpha=0.05, p<0.001). This is also predictably stronger than the correlation between SMP and SNP sites, even given the low number of CpG-DMRs for comparison with CpG-SMPs that may decrease our ability to detect a strong correlation.

Supplemental note S9: Accessions cluster by preferentially targeted genes and gene families

Accessions were clustered based on overlapping gene families that were targeted by methylation. There was a large variation in the number of genes within each analyzed family therefore a proportional representation of methylated targeted genes per family was used (Figure 3d). In Figure 3d, unlike accessions from smaller countries, those from geographically larger countries, e.g. China, USSR and India appear spread out into different clusters; this is reflective of the clustering seen using SMP information. The distribution of the colour barcodes for SNPs and SMPs also indicates that there is an association between these features and accession clusters based on the preferentially targeted gene families, however differences are observed that are likely to be due to the method of clustering used here. This method highlights which gene families are preferentially methylated, but it is less useful to cluster accessions since it only addresses levels of family methylation rather than if the same genes are targeted specifically. Furthermore, large and small gene families are given the same weighting for clustering.

In Figure 3d it is clear that a number of gene families were preferentially targeted for methylation and hierarchical clustering shows the presence of these preferentially targeted gene families (horizontal dendrogram). All genes in the highly targeted gene families within and between accessions (696 and 235 genes respectively) were subjected to enrichment of molecular functions using topGO (p <0.01, weight01 GO scoring for Fisher's exact test) and Supplemental Tables S17 and S18 provide an overview of the most significantly enriched GO terms and associated molecular functions. Six molecular functions (28.6%) were common between the highly targeted gene families within and between accessions, while the majority, 13 (61.9%) were unique to comparisons between accessions.

We also performed GO enrichment on genes from the predominantly gray regions (heatmap, Figure 3d), which contain genes that were targeted by methylation but to a lesser extent. The gene sets consisted of 1774 and 1017 genes from within and between accessions, respectively (Supplemental Table S19 and S20).

Supplemental note S10: Differential expression and methylation associate with metal ion transportation

Genes with differential expression and methylation that contributed to enriched molecular functions for metal ions and transportation include ATP-dependent zinc metalloprotease *FTSH*2, a Sodium/hydrogen exchanger, ABC transporter C, Aquaporin *SIP1-2* and Protein DETOXIFICATION (Supplemental Table S25);

a) Two genes showed up-regulated expression from a (former) Yugoslavian cluster 1a accession 1190352. The first gene is a Sodium/hydrogen exchanger showing a >50% increase in CpG gene-body methylation compared to the accession 1190292-Cyprus in cluster 2c. The second is an ABC transporter C family member involved in detoxification that showed a >50% decrease in first-exon methylation compared to the accession 1190181-Poland in cluster 2f. These methylation and gene expression correlations fit the directionality models predicted by previous studies for gene-body and first exon methylation (Yang et al 2014;

Brenet et al 2011). Furthermore, previous analyses have shown metals such as potassium, uranium and mercury to be at typically lower concentrations in soils across Cyprus and Poland compared to the Yugoslavian region where concentrations were increased. Therefore, this change in gene expression could be part of an adaptive response (Reimann et al 2014; Ottesen et al 2013).

b) The gene ATP-dependent zinc metalloprotease *FTSH2* showed up-regulation in the Palestinian accession 1190398 coupled with a consistent decrease in CpG methylation of >50% in the 3'UTR compared to accessions 1190292, 1190181, 1190481, 1190103, 1190299, 1190352, 1190308, 1190141 and 1190777. Hypermethylation at 3'UTR's was previously linked to decreased expression due to effects on transcription and gene splicing so the mirrored effect of hypomethylation seen here with gene up-regulation fits the directional model (Maussion et al 2014).

c) The gene Aquaporin *SIP1-2* showed up-regulation in the Polish accession 1190481 coupled with increased CpG gene body methylation >50% compared to accession 1190034. This result fits the directionality models predicted by previous studies for gene-body methylation having a positive effect on expression.

d) The gene Protein DETOXIFICATION showed up-regulation in the Palestinian accession 1190398 coupled with an increase in CHH methylation >20% overlapping the beginning of the 5'UTR compared to accessions 1190034, 1190292 and 1190299.

Supplemental note S11: Early heading date associates with similar global SMP but different SNP profiles

a) Forty six annotated genes were differentially expressed between heading dates, but direct methylation data was available for 2 of them; 26S protease regulatory subunit 7 showed a 3.9% decrease in gene body methylation correlating with a down-regulation of expression across accessions with early heading dates and the gene *FAR1*-RELATED SEQUENCE 5-like showed a 5% increase in promoter methylation correlating with a down-regulation in gene expression across accessions with early heading dates compared to late heading. Here, we look at methylation in only 2 of the 46 differentially expressed genes between heading dates; within this study we can only confidently cluster accessions by global methylation profile, we do not have sufficient information for all of the 46 genes of interest, that were identified from the RNA-seq analysis, to make further methylation inferences.

b) The top two enriched GO terms and associated biological processes from analysis of the 46 differentially expressed genes relate to the gene Chromatin assembly factor 1 (*CAF-1*) that is down-regulated in early heading plants (Supplemental Table S28). *CAF-1* ensures stable inheritance of epigenetic states through plant development and its absence may leave replicated DNA naked and easily accessible, therefore, increasing the probability of transcription (Ono et al 2006). It is therefore possible that increased transcriptional activity could assist a plant in increasing its rate of development or growth.

Supplemental note S12: Transposon assessment through the analysis of off target sequence

a) Chinese Spring bisulfite-treated sequencing data that could not be mapped to the reference sequence, was aligned to the non-redundant TREP nucleotide database of *Triticeae* transposable elements (TEs) (Wicker et

al 2002). This off-target sequence should be unbiased carryover DNA and is equivalent to shotgun sequencing of total wheat DNA at low coverage. To test this, we compared the proportions of transposon types in the enriched data for Chinese Spring and found that they closely matched the proportions in previous shotgun sequence data (Brenchley et al 2012) (Supplemental Table S29). From Supplemental Table S29, the only notable difference is the proportion of sequence data aligning to the SINE retrotransposons, which is greatly increased in our analysis compared to that of Brenchley et al. However, the TREP database has progressed from 2012 when the Brenchley et al study was carried out. Since then the SINE retrotransposon reference has increased in size from only one contig of 256bp to 128 contigs covering 412,949bp. This may explain the increased proportion of reads associating with this retrotransposon type.

b) To assess TE methylation levels for each Watkins accession individually, off-target sequencing data was aligned to the collapsed wheat TREP database as per the methodology for Chinese Spring. For each transposon type, mapped reads were analyzed across all of the cytosine residues and the overall percentage of mapped reads showing methylation was calculated. Transposons were hyper-methylated; for gene-associated enriched regions if we average the percentages of methylated cytosines across the accessions we see 61.5%, 19.4% and 1.69% methylated across CpG, CHG and CHH sites respectively, whilst for TE associated sequences we see corresponding values of 82.0%, 58.0% and 3.2% respectively (Supplemental Table S30). In particular, non-CpG methylation shows an almost 3-fold increase in methylation in TEs.

c) Although TE percentage methylation levels were stable across the Watkins collection, there was variation in the proportions of reads aligning to each transposon type after coverage normalization (Methods). Using normalized read counts and mapping to a collapsed TREP database, we observe the explosion of a specific TE group as an increase in the proportion of reads mapping to this group with the additional proportional drop-out of coverage from other groups. This occurs since the collapsed reference contigs for the expanded TE group represents more sequence than represented previously and therefore attract a larger proportion of the off-target sequence. As such, for each transposon type, we used Chinese Spring as a baseline and compared the cumulative mapping coverage for each accession to Chinese Spring to ascertain transposon variability (Figure 4, Methods).

From figure 4a, both DNA transposon and retrotransposon expansion can be observed depending on the specific Watkins accession being compared to the baseline of Chinese Spring. Expansion of retrotransposons is more frequently observed across the collection although some of the largest expansions are seen in a small subset of lines in DNA transposons. For DNA transposons, it appears that expansion within the TIR; CACTA group specifically are responsible for increasing the proportion of DNA transposons compared to Chinese Spring in a subset of Watkins accessions (Figure 4b). This expanded group of DNA transposons showed conservation of the high methylation levels across TEs (Figure 4i) but no corresponding increase in gene body methylation was observed in these accessions (Figure 4f). Focusing on retrotransposons it appears that only SINE and LTR; Gypsy groups show prominent increases compared to Chinese Spring and there is variability in the extent of these increases across the Watkins collection (figure 4c). The expanded groups of SINE and LTR; Gypsy retrotransposons showed conservation of the high methylation levels seen across TEs (Figure 4g and 4h)- although no corresponding increase in gene body methylation was observed (Figure 4d and 4e).

Supplemental Figures

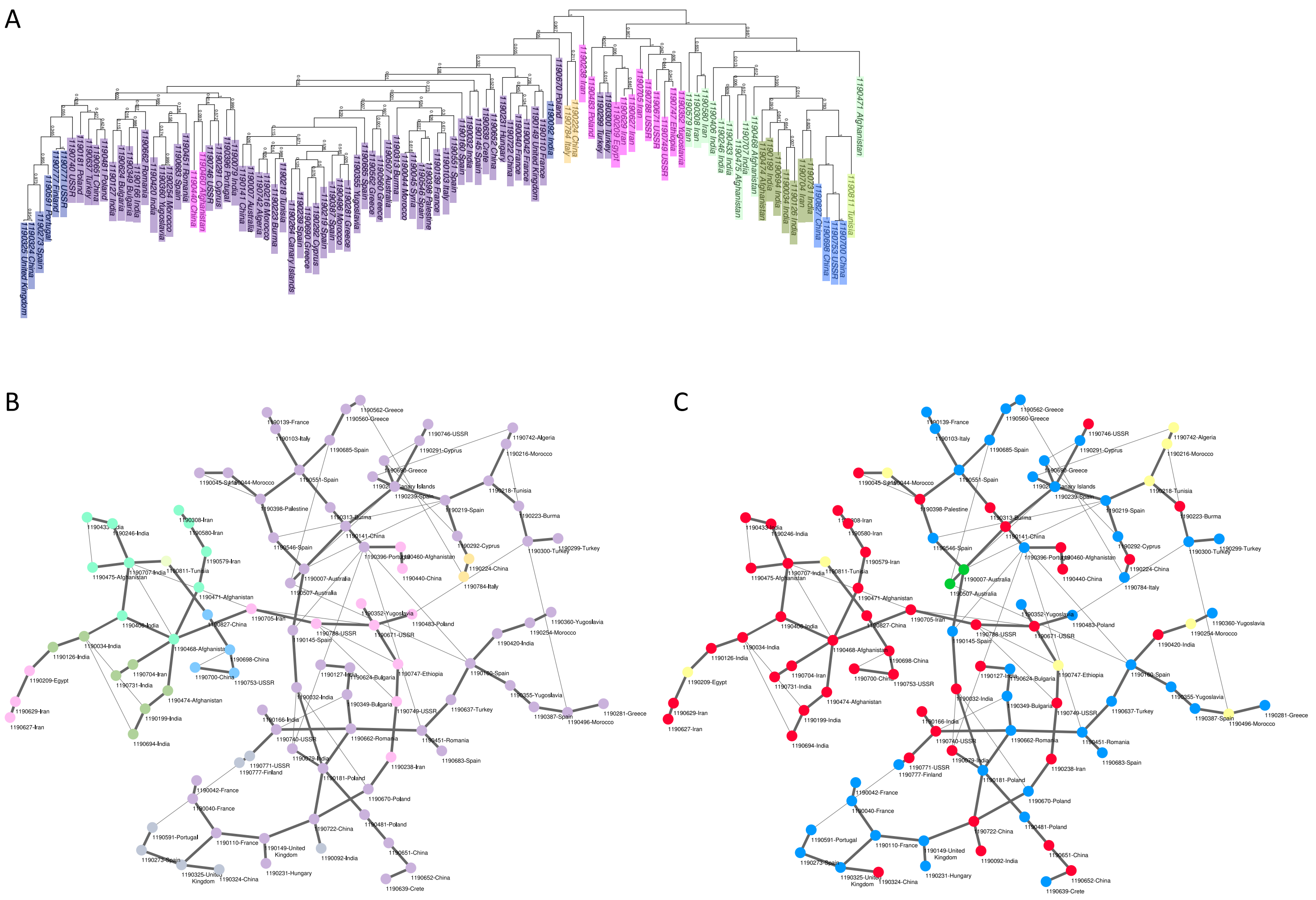

Supplemental Figure S1. Maximum likelihood cluster analysis on the 104 Watkins accessions. (A) Phylogenetic tree constructed using the Maximum likelihood method to cluster accessions based on SNP allele frequency across 53,341 SNP sites. The tree is colour coded based on the 8 groups defined in Figure 1b (Methods). **(B)** Consensus network constructed using the tree from (A) using the same colour code for accessions (Methods). **(C)** depicts the same network as seen in **(B)** however here colour coding is according to continent of origin; Asia (Red) Europe (Blue) Africa (Yellow) Australia (Green)

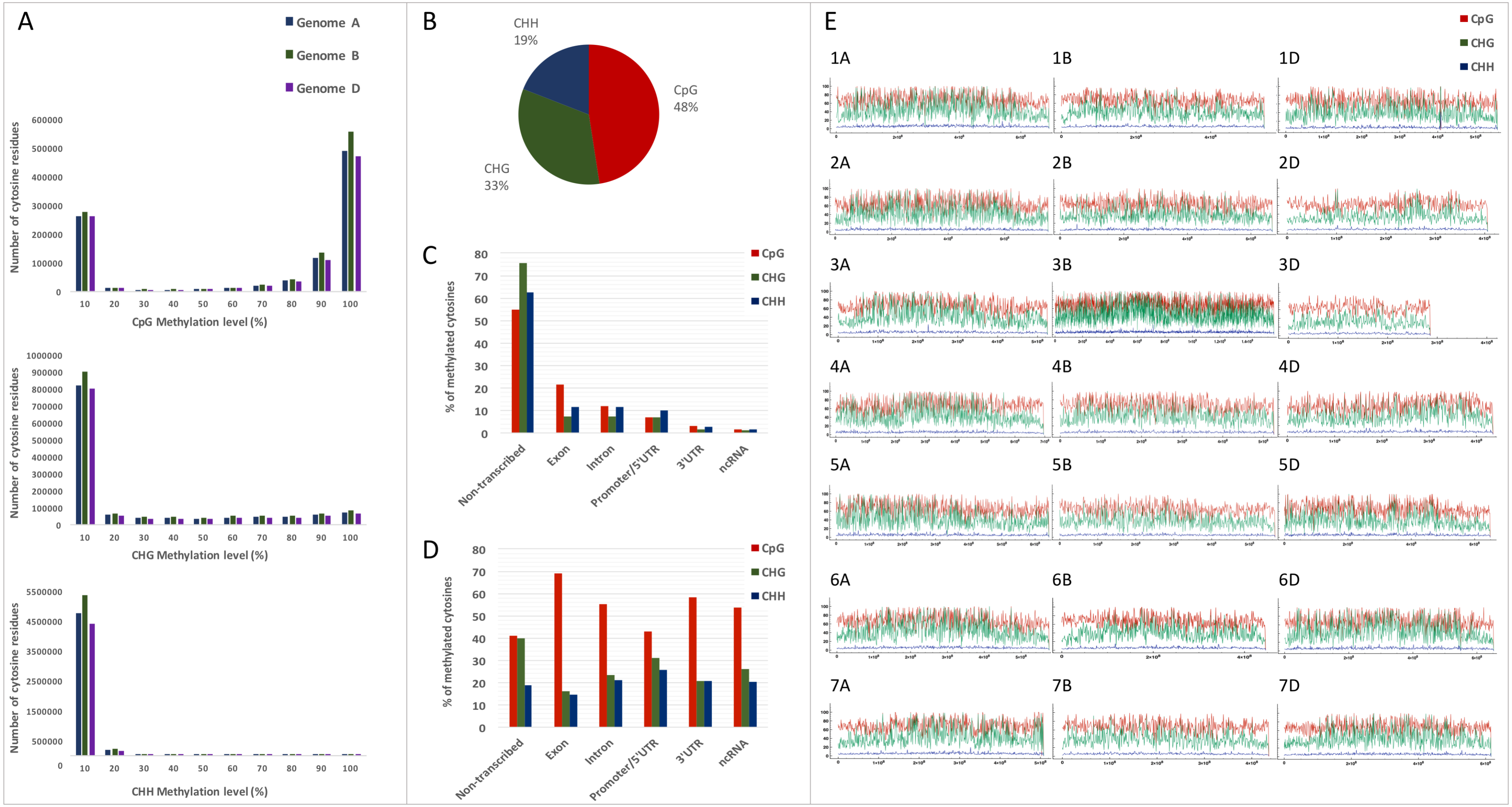

Supplemental Figure S2. General patterns of methylation in Chinese Spring. (**A**) Number of CpG, CHG, and CHH sites at each methylation level across the A, B and D sub-genomes of wheat. The *x*-axis is divided into 10 individual bins that correspond to methylation levels 0-100%. The *y*-axis is the total cytosine residue counts for each respective bin. Here sites are only utilized if they have a minimum depth of 10X. **(B)** Distribution of methylated sites between CpG, CHG and CHH sites **(C)** Methylation sites are firstly categorized by cytosine residue status i.e. CpG, CHG and CHH, then within these categories the percentage of sites in each gene location category is shown (Non-transcribed, Exon, Intron, Promoter, 3'UTR or ncRNA). **(D)** Methylation sites are firstly categorized by gene location, then, within these categories, the percentages of sites with CpG, CHG or CHH status are shown. **(E)** Percentage of CpG, CHG or CHH sites that are methylated per 1Mbp window along each wheat chromosome (start position of 1Mbp window displayed).

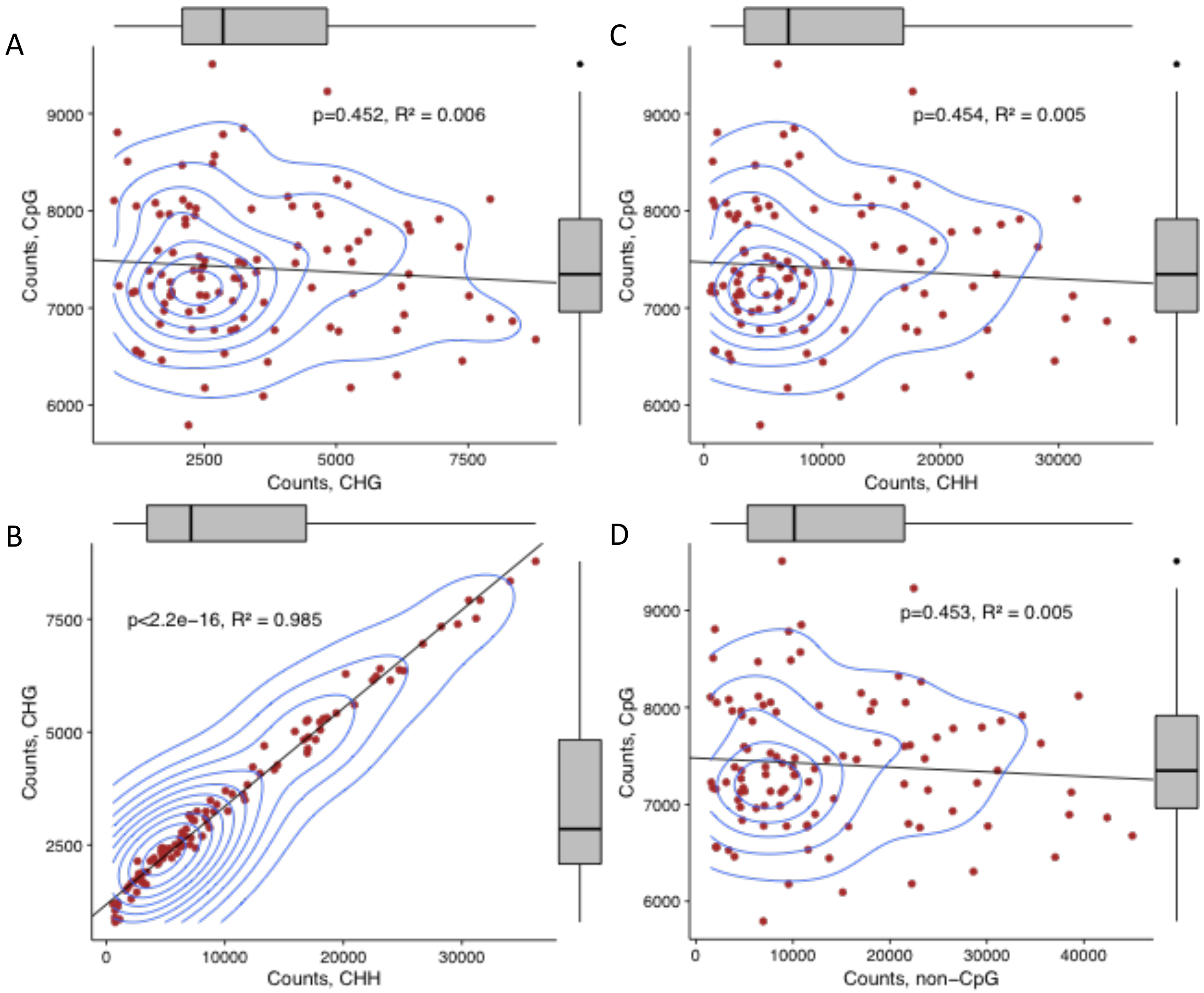

Supplemental Figure S3. Association between methylation contexts (CpG/CHG/CHH). (A)-(D) Regression model fit to scatter plots of methylation contexts for the 105 accessions. Counts of the number of methylated sites for the contexts plotted against the other, and each dot corresponds to an accession.

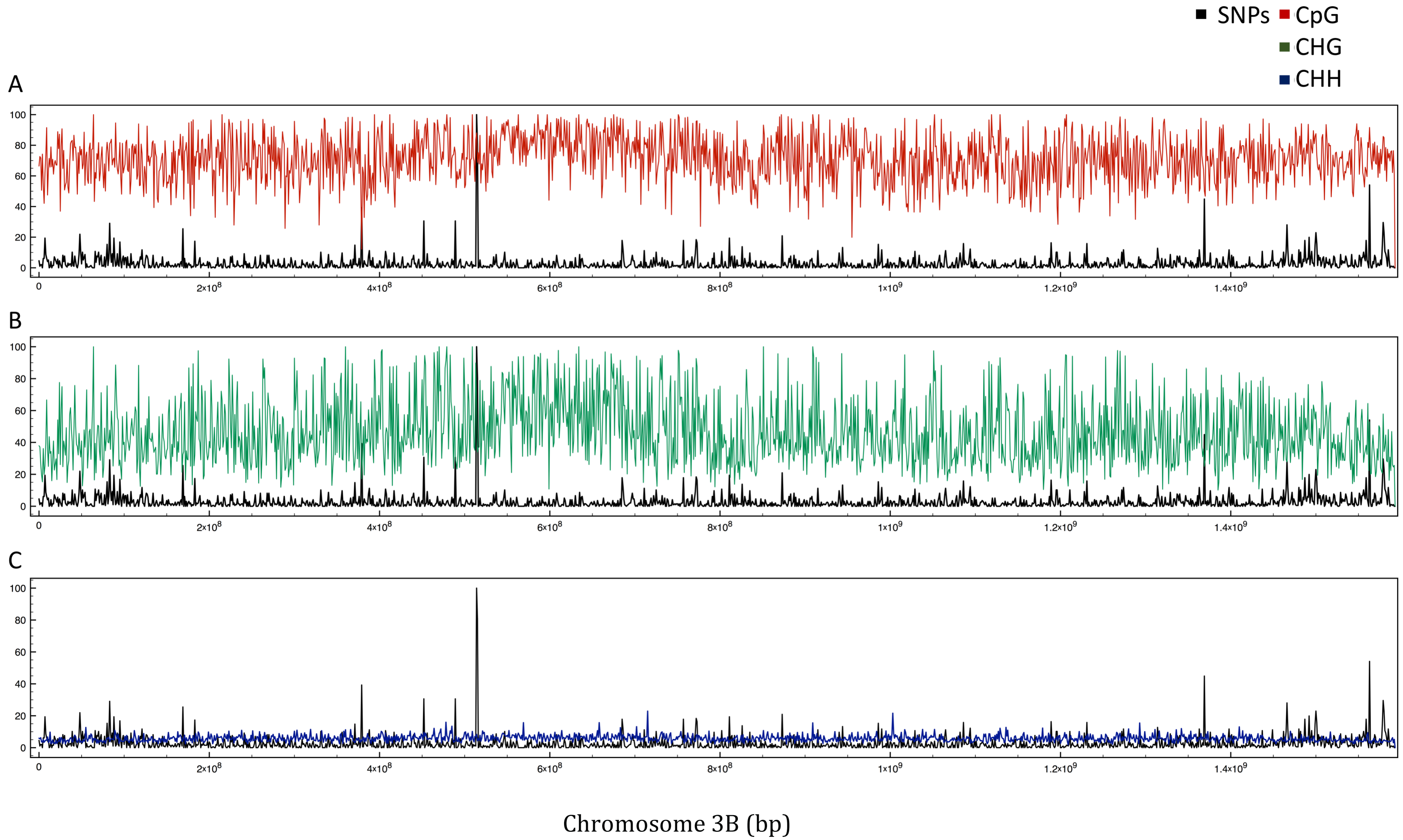

Supplemental Figure S4. Methylation versus SNP frequency in Chinese Spring. Percentage of **(A)** CpG, **(B)** CHG or **(C)** CHH sites that are methylated per 1Mbp window along each wheat chromosome (start position of 1Mbp window displayed). In addition, each plot shows the frequency of SNPs per 1Mbp (normalised to a 0-100 scale).

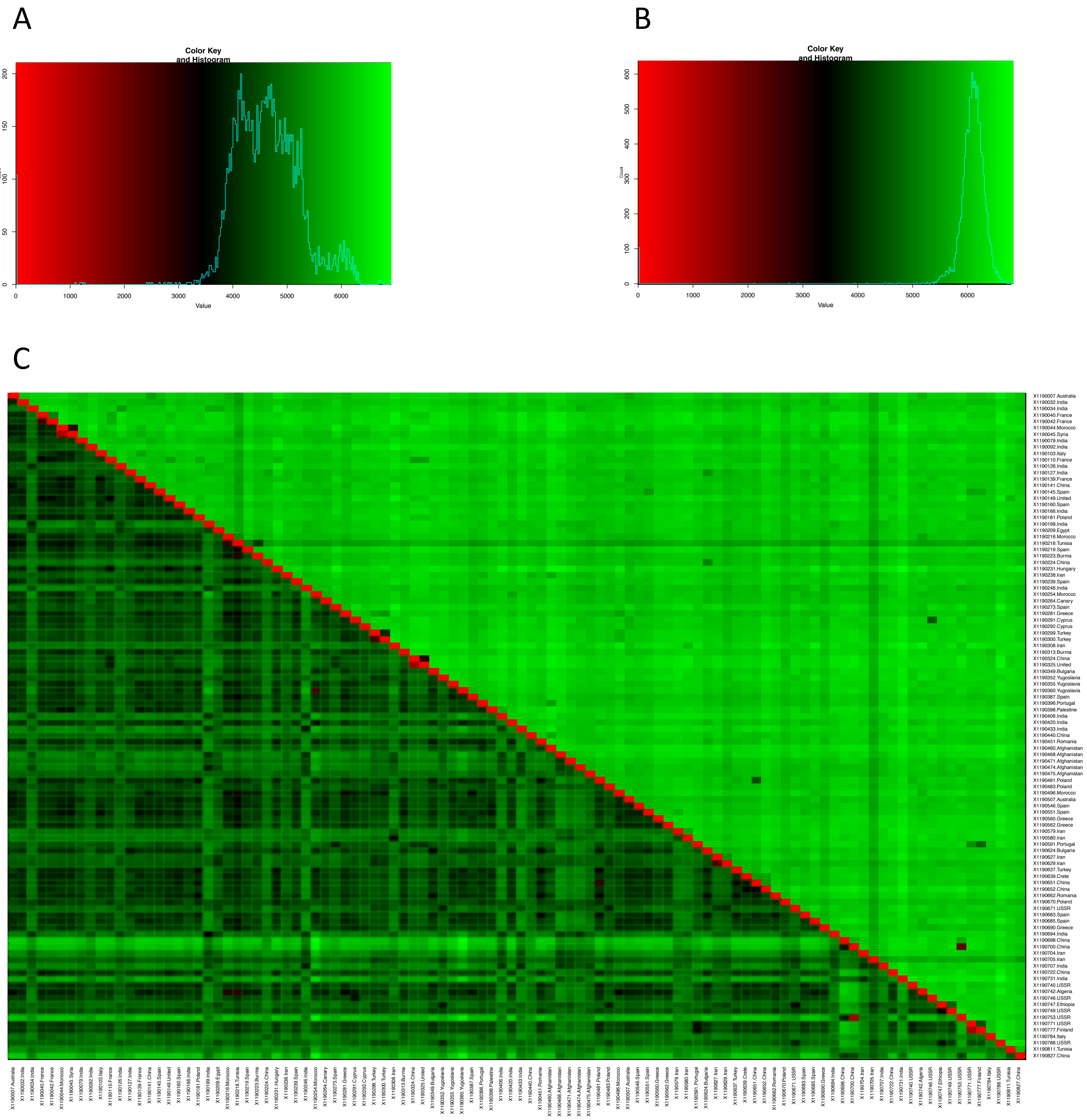

Supplemental Figure S5. Combined distance matrices for SNPs and SMPs from 104 accessions from the Watkins core collection. (A) Colour key and histogram for SNP distance matrix. **(B)** Colour key and histogram for SMP distance matrix. **(C)** Combined distance matrices; lower triangle corresponds to matrix based on a subset of the 53,341 variable SNP sites and upper triangle corresponds to matrix based on 18,965 CpG SMP sites. Distance matrices constructed using the R function dist.

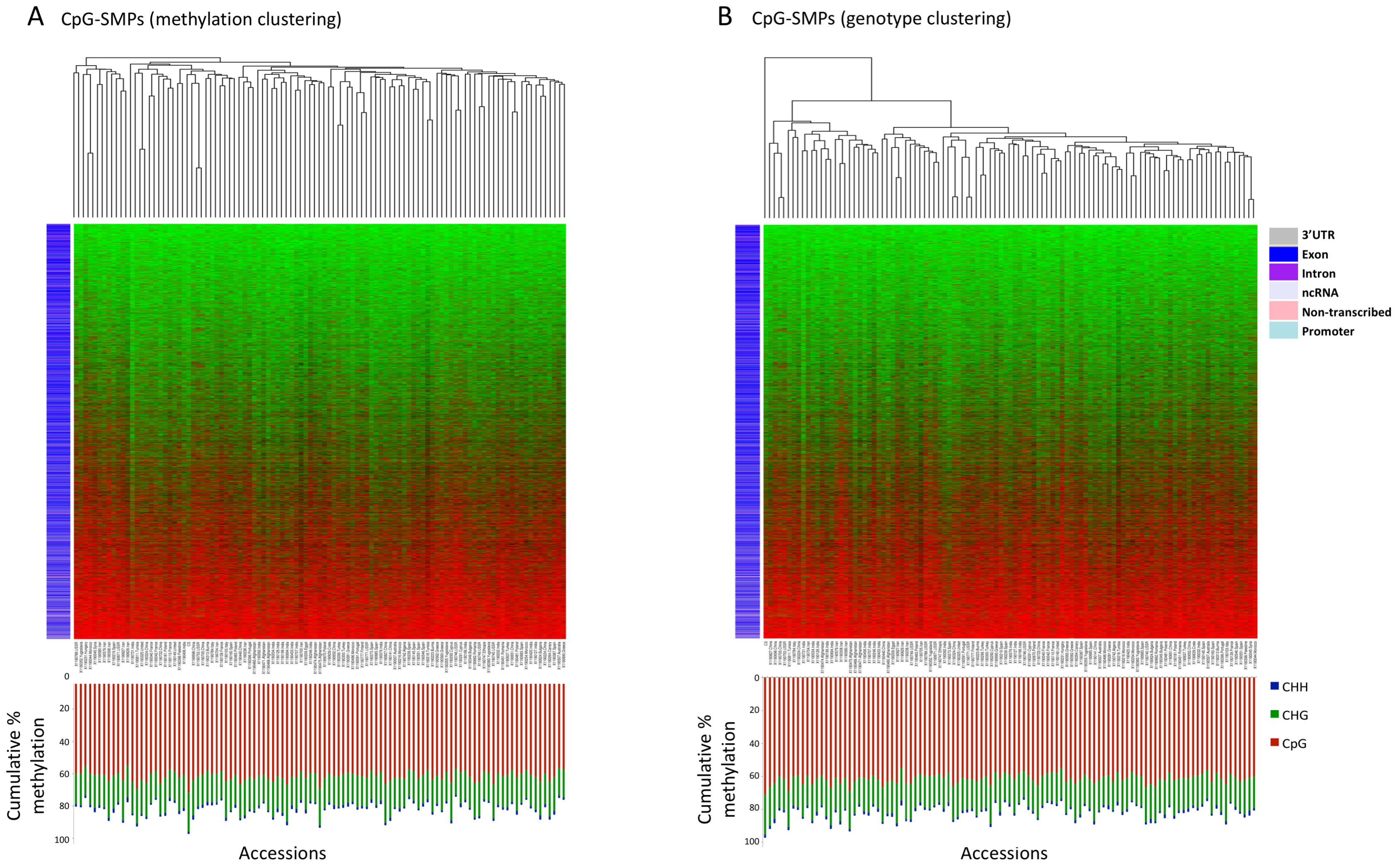

Supplemental Figure S6. Visualizing methylation levels for the 105 wheat accessions across 359,500 SMP sites. Using sites with coverage in all 104 Watkins collection accessions plus Chinese Spring we generated heatmaps for methylation levels across **(A)** CpG-SMPs (taken from figure 2a) and (**B**) CpG-SMPs with accessions (taken from figure 2b). The coloured row labels on the left of the heatmap indicate which genomic location a SMP falls into (see legend). Rows indicate the genomic locus of the SMP and columns indicate accessions. The stacked column charts represent the overall methylation level in each accession for CpG, CHG and CHH contexts and columns correspond to the accession labelled directly above in the heatmap.

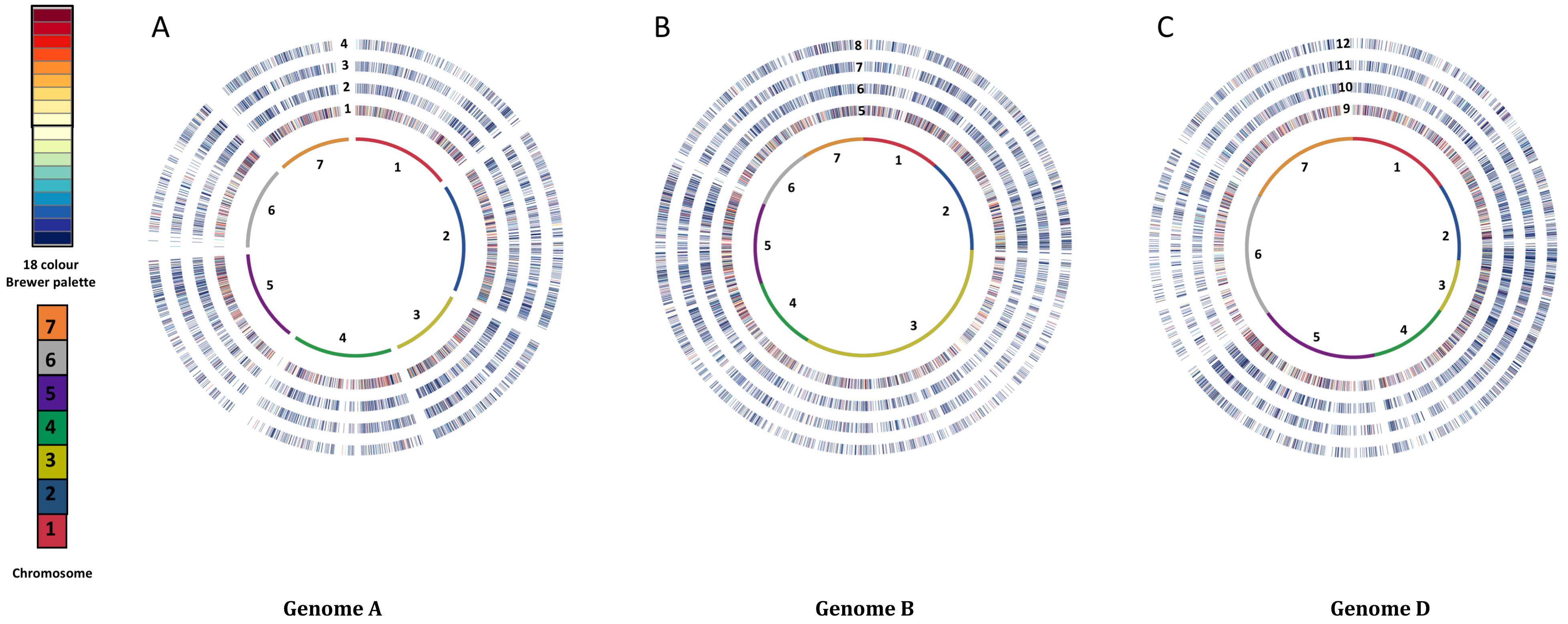

Supplemental Figure S7. Uni-, bi- and tri-genome methylation conservation across the 104 Watkins accessions plus Chinese Spring. The software Circos was implemented to produce circular heat maps for all uni-, bi- and tri-genome methylation sites that showed 10X or more coverage across the 105 accessions. An 18 colour Brewer palette scale of blue to red was used to represent methylation site conservation at each site in 1 to 105 accessions respectively (see methods). **Track 1, 5 and 9** Tri-genome methylation in sub-genome A, B and D respectively. **Track 2, 6** Bi-genome AB methylation in sub-genomes A and B respectively. **Track 3, 10** Bi-genome AD methylation in sub-genomes A and D respectively. **Track 4, 8, 12** Uni-genome methylation in sub-genomes A, B and D respectively. **Track 7, 11** Bi-genome BD methylation in sub-genomes B and D respectively.

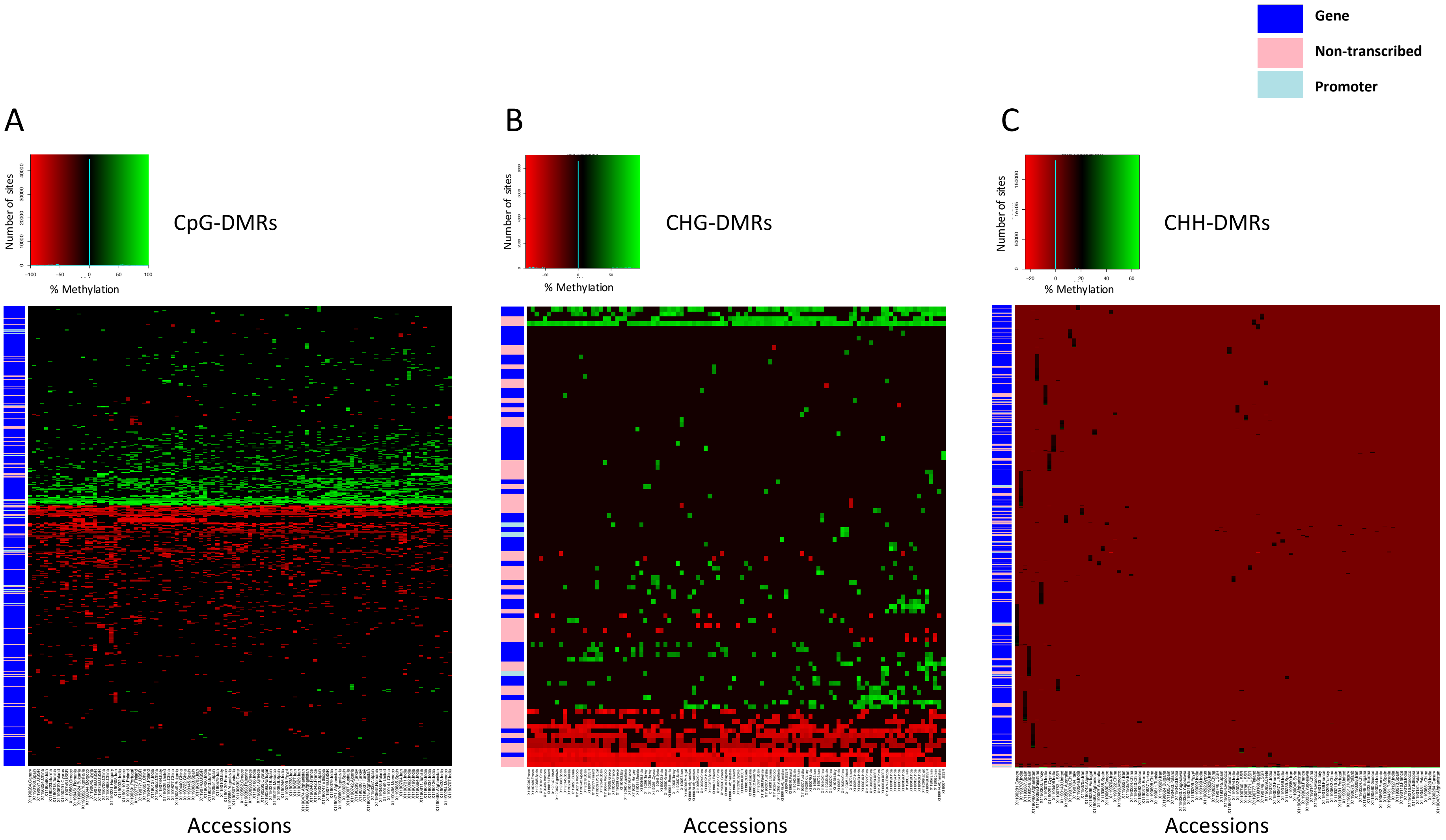

Supplemental Figure S8. Visualizing methylation levels for the 105 wheat accessions across DMRs. Heatmaps representing the methylation levels across (**A**) 491 CpG-DMRs (**B**) 96 CHG-DMRs and (**C**) 1769 CHH-DMRs with coverage in all 104 Watkins collection accessions plus Chinese Spring. The coloured row labels on the left of the heatmap indicate which genomic location a SMP falls into (see legend). Rows indicate the genomic locus of the 100bp DMR and columns indicate accessions

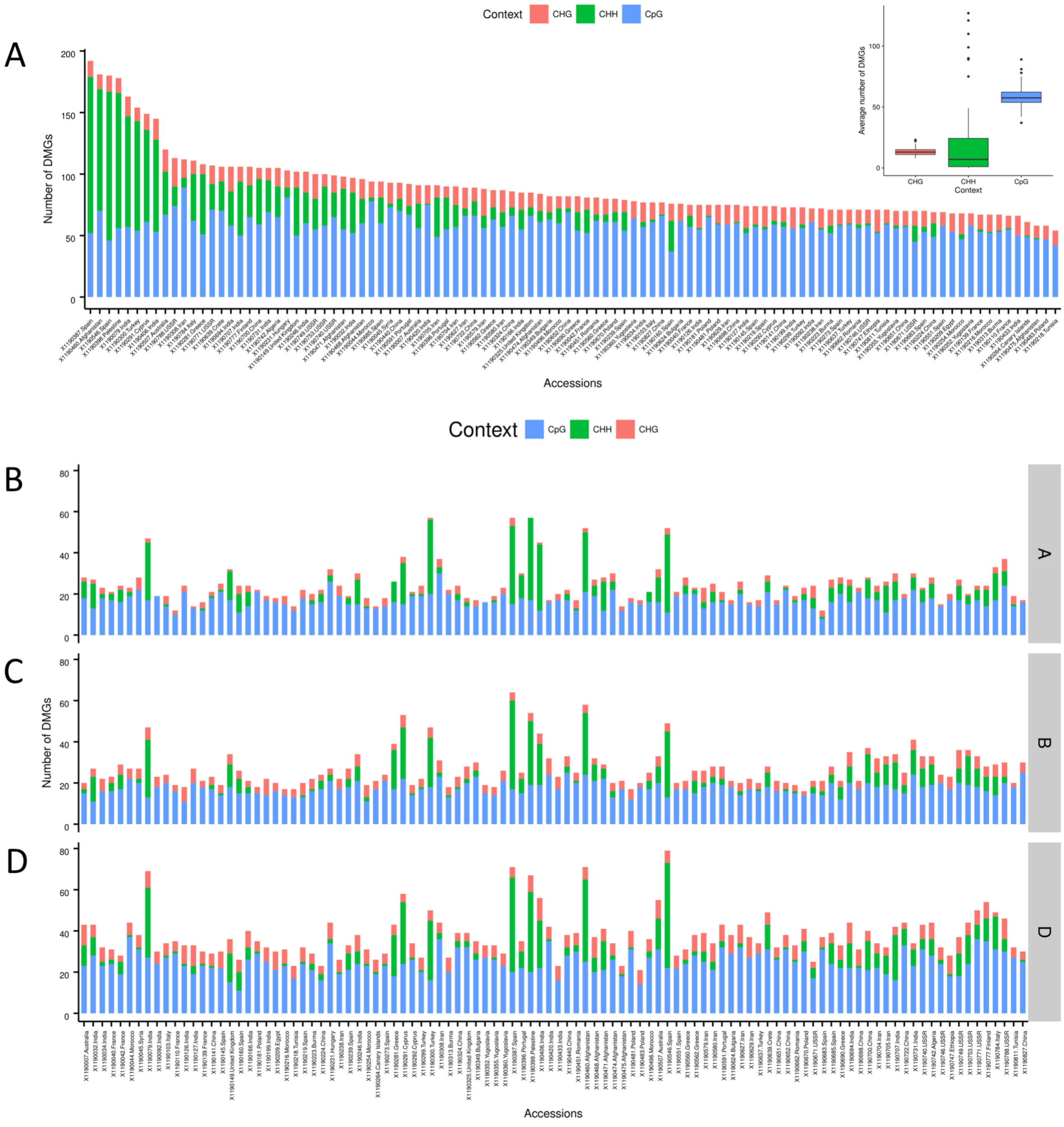

Supplemental Figure S9. Context of differential methylation of genes across the 104 accessions. Distribution of the number of differentially methylated genes, DMGs. DMGs represent genes showing DMRs compared to Chinese Spring. **(A)** Both known and unknown DMGs categorized by methylation context. **(B)-(D)** Distribution of number of DMGs across sub-genomes A, B and D.

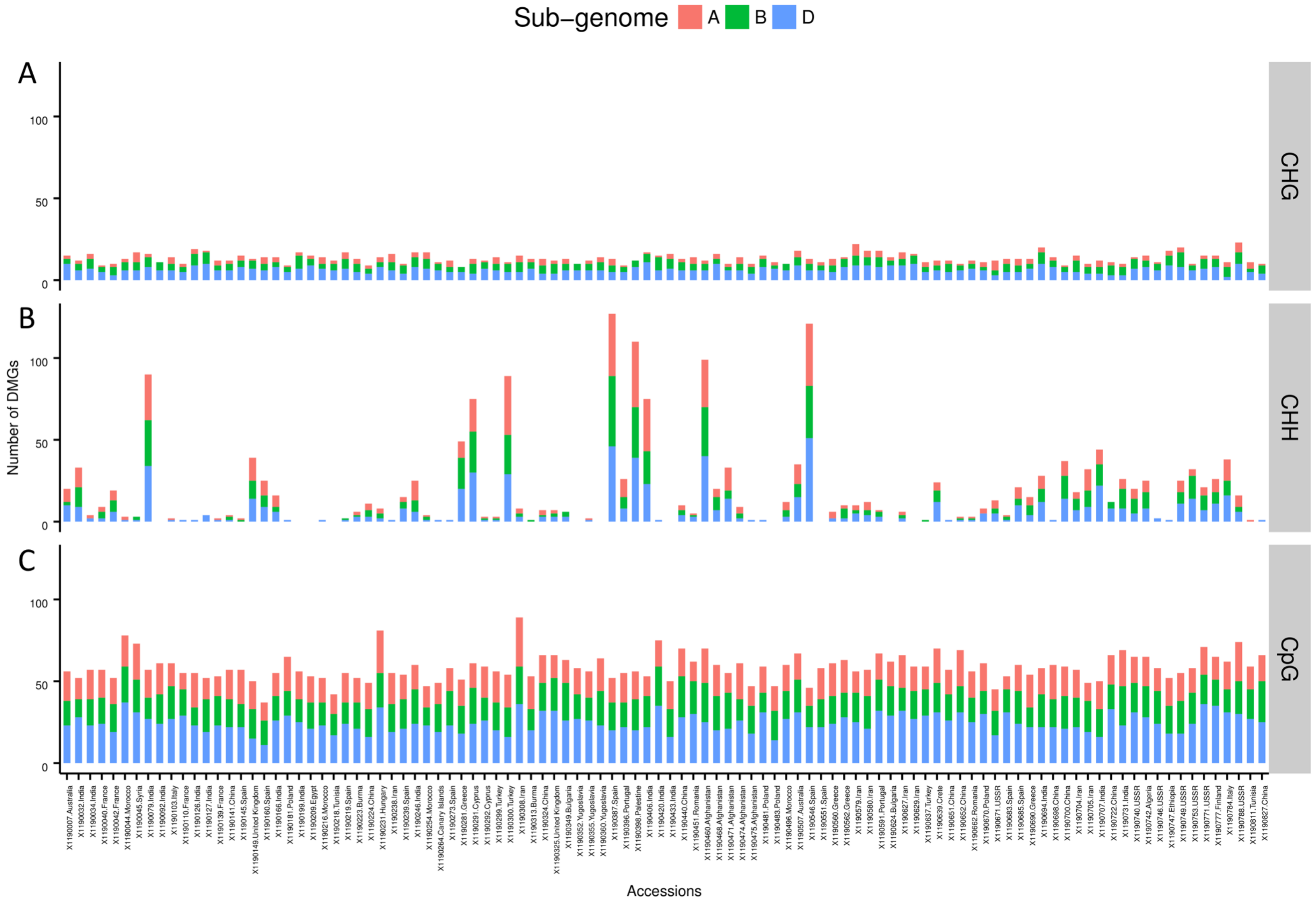

Supplemental Figure S10. Sub-genome specific context of differential methylation of genes across the 104 accessions. (A)-(C) Distribution of the number of differentially methylated genes (see figure 11) by methylation context (CpG/CHG/CHH) across sub-genomes A, B and D.

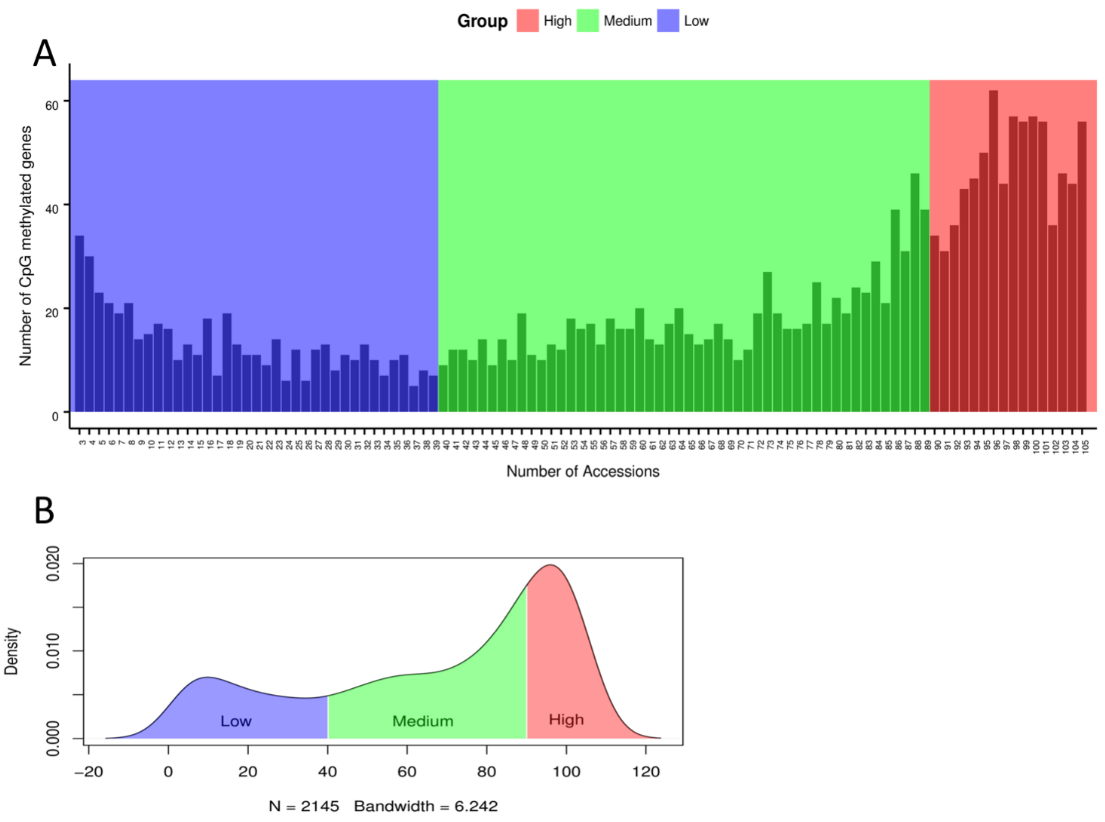

Supplemental Figure S11. Distribution of CpG methylated genes across accessions. (A) Bar graph of CpG methylated genes categorized into 3 groups (high, 753 genes; medium, 897; and low, 495). **(B)** Corresponding density plot for the CpG methylated genes.

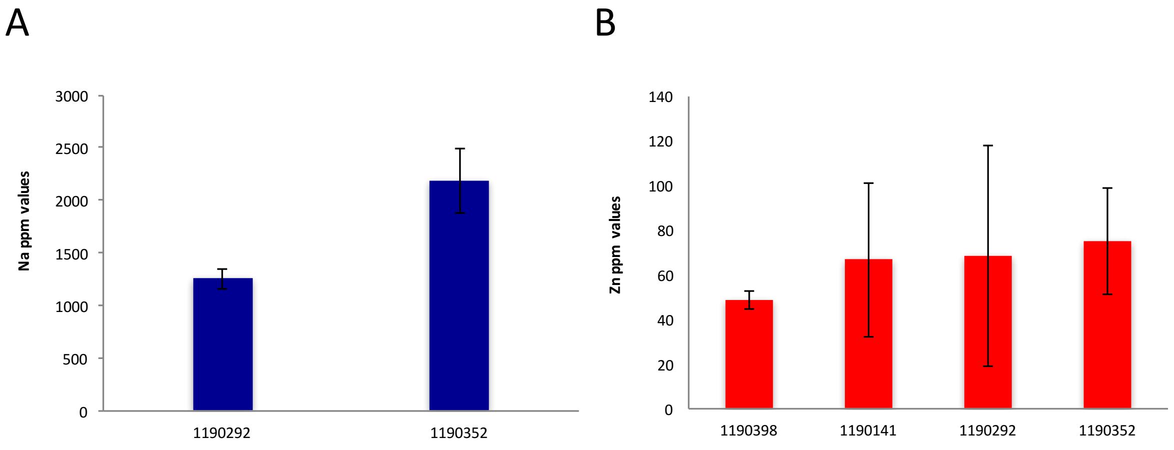

Supplemental Figure S12. Metal ion concentration in wheat leaves from the Watkins collection. (A) Na (sodium) concentrations in ppm **(B)** Zn (zinc) concentrations in ppm. Bar charts illustrate average concentrations across three replicates, error bars show standard deviations between replicates.

Supplemental Tables

Watkins Line	Country of origin	Watkins Line	Country of origin	Watkins Line	Country of origin	Watkins Line	Country of origin
1190007	Australia	1190239	Spain	1190474	Afghanistan	1190704	Iran
1190032	India	1190246	India	1190475	Afghanistan	1190705	Iran
1190034	India	1190254	Morocco	1190481	Poland	1190707	India
1190040	France	1190264	Canary Islands	1190483	Poland	1190722	China
1190042	France	1190273	Spain	1190496	Morocco	1190731	India
1190044	Morocco	1190281	Greece	1190507	Australia	1190740	USSR
1190045	Syria	1190291	Cyprus	1190546	Spain	1190742	Algeria
1190079	India	1190292	Cyprus	1190551	Spain	1190746	USSR
1190092	India	1190299	Turkey	1190560	Greece	1190747	Ethiopia
1190103	Italy	1190300	Turkey	1190562	Greece	1190749	USSR
1190110	France	1190308	Iran	1190579	Iran	1190753	USSR
1190126	India	1190313	Burma	1190580	Iran	1190771	USSR
1190127	India	1190324	China	1190591	Portugal	1190777	Finland
1190139	France	1190325	UK	1190624	Bulgaria	1190784	Italy
1190141	China	1190349	Bulgaria	1190627	Iran	1190788	USSR
1190145	Spain	1190352	Yugoslavia	1190629	Iran	1190811	Tunisia
1190149	UK	1190355	Yugoslavia	1190637	Turkey	1190827	China
1190160	Spain	1190360	Yugoslavia	1190639	Crete		
1190166	India	1190387	Spain	1190651	China		
1190181	Poland	1190396	Portugal	1190652	China		
1190199	India	1190398	Palestine	1190662	Romania		
1190209	Egypt	1190406	India	1190670	Poland		
1190216	Morocco	1190420	India	1190671	USSR		
1190218	Tunisia	1190433	India	1190683	Spain		
1190219	Spain	1190440	China	1190685	Spain		
1190223	Burma	1190451	Romania	1190690	Greece		
1190224	China	1190460	Afghanistan	1190694	India		
1190231	Hungary	1190468	Afghanistan	1190698	China		
1190238	Iran	1190471	Afghanistan	1190700	China		

Supplemental Table S1. 104 lines from the core set of the Watkins bread wheat landrace collection. These are the Watkins lines that were sequenced for analysis in this study.

Accession	Number of reads	Mapped bp	Mapped bp (10x min)	Final number mapped reads	Number of cytosines	Number of cytosines (10x min)
1190007	58298174	280639264	37812188	10227682	69004056	8537602
1190032	59424158	279810539	36692486	10019328	69663986	8478351
1190034	61213408	416179938	64840594	17957264	103869428	14297940
1190040	70635140	279422841	43659871	11571750	69293190	10015825
1190042	88291932	237787641	35195282	9438914	58488239	8148913
1190044	37776350	359763053	52539617	14195980	89210833	11675493
1190045	88567762	286902444	37146727	9822402	70430372	8392593
1190079	81589344	277336495	41533354	10816496	68276540	9478334
1190092	98749936	251692871	39485434	10343708	61893100	9131411
1190103	52728122	665443453	103007328	39431722	173561917	22749709
1190110	89479950	275470835	37668357	10139388	69279549	8872266
1190126	182271992	213702604	33907813	9021308	52604293	7905024
1190127	57861372	382792421	56930798	15564764	95677888	12730160
1190139	66745680	342476659	56019875	15081944	85121908	12633363
1190141	55643898	689171191	106191536	41863722	181583103	23642083
1190145	63410754	314438843	48308397	13097394	78198971	11044290
1190149	82311354	260621725	35646314	9689396	64686615	8344658
1190160	80546420	242750002	33757920	9116158	59490892	7722865
1190166	78823140	265111041	36329861	9891256	66119476	8459884
1190181	153264968	404907660	59684534	16495582	101762883	13447838
1190199	136318882	287914433	38213987	10332882	70921734	8733043
1190209	184565590	262060629	39840681	10565974	64108797	9158868
1190216	125457690	243752741	36035078	9561718	59519606	8268399
1190218	135521318	230429781	32771716	8840712	56743046	7660379
1190219	183512478	207515506	25637942	7467302	50888436	6124812
1190223	72684442	437598164	71429800	20321462	110011794	15897205
1190224	60007556	354639753	56604014	15648780	88736683	12861149
1190231	63131166	297343410	43510493	11924524	74335403	10079530
1190238	58650854	712064427	113524662	45000570	187389382	25231088
1190239	59960184	306442037	48057612	13187952	76906593	11108435
1190246	76327376	276279987	41586323	10940460	67881431	9467069
1190254	66255448	279542943	43554842	11592064	69203427	10003296
1190264	192092030	435255002	70099620	19534652	108568957	15604345
1190273	134725066	338814838	53237255	14560010	85100083	12349366
1190281	169305296	337881725	51055864	13845326	84838110	11829184
1190291	114948088	234965334	34332604	9164498	57895936	8018562
1190292	52623708	667873495	103237876	39497116	173754318	22743098
1190299	66272146	357520154	56444057	15262814	88557472	12607054
1190300	98144634	256929188	36390170	9644948	63581574	8398210
1190308	54763950	694469153	106931123	41105626	181217196	23561586
1190313	183822364	202481112	27228618	7685344	50262670	6565849
1190324	65438292	431639482	71882248	19930672	106821122	15721327
1190325	62313948	327578886	48135290	12802858	80636524	10739786
1190349	40680558	283637257	40463876	10802364	69852587	9141660
1190352	67941620	268438154	37447519	10051694	66390858	8603960
1190355	81400862	265129222	40229901	10737632	65398973	9306700
1190360	80649908	253305723	36923120	9916498	62631722	8515628
1190387	134541670	395454998	55453099	14722772	97635322	12188393
1190396	121984370	333754017	49414540	13149806	82413901	11141824
1190398	159608934	315159763	40301408	11172614	77719318	9306825
1190406	93592420	266438293	37149308	9874632	65952916	8608615
1190420	92744206	266504646	37970613	9926252	65502444	8674284
1190433	124617490	206781786	31997215	8383384	50479162	7405635
1190440	66275442	409908084	64278852	17360288	101255134	14033783
1190451	57020610	328755485	51205170	13511552	80766741	11381259
1190460	82928742	187520370	21060533	6151610	44991210	4823764

1190468	73128188	296146794	42742983	11347818	73184339	9682515
1190471	70854740	256525743	36028246	9657330	63166539	8293436
1190474	65881408	272680106	40325140	10639402	67299152	9209155
1190475	163635408	491178844	82503074	23022868	121023838	17801991
1190481	121922176	316165349	43257651	11488806	78178797	9785894
1190483	139340876	305286873	44319918	11693694	75343383	10063965
1190496	92049468	266100075	42264440	11075604	65190303	9642222
1190507	74511548	254232952	38067927	9954056	62705791	8835420
1190546	121013904	231899113	33131780	8757278	56835763	7701362
1190551	62579168	350575095	55079063	14660032	86117868	12133073
1190560	55308664	292349317	43253133	11302132	71154823	9590202
1190562	68456950	256028799	37798803	9928928	62308605	8537225
1190579	91604506	248005073	36281885	9686370	60859128	8375664
1190580	81879596	255981671	37045531	9822578	62666210	8475860
1190591	52806652	260470565	37408166	9901610	64080826	8511177
1190624	152509950	338930873	47082831	12500064	82927266	10392624
1190627	55263242	678863856	105945065	41431398	178505569	23610993
1190629	149434604	269630608	36410439	9985962	67330256	8696908
1190637	145321392	269640888	38140605	10128238	66419667	8752166
1190639	106596060	250612459	39273407	10260296	61380283	9026510
1190651	133382512	217661310	31233585	8498286	53882460	7464017
1190652	126330308	211673546	28711171	7766962	51666032	6704629
1190662	45609078	359375968	52229219	13931354	88110873	11384020
1190670	57138300	326024571	50043385	13215054	79523167	11068285
1190671	70797174	281913780	38487311	10132526	68832711	8652789
1190683	57270274	257735271	37940780	9972534	63115250	8652418
1190685	78376044	245006580	35823026	9544964	60273253	8271453
1190690	90730230	241819400	35956391	9493718	59344507	8329226
1190694	48182428	312433668	38382596	10360296	75720530	8410726
1190698	89551280	293718668	38504472	10236214	71619016	8602178
1190700	87355516	304405035	43628593	11394990	74545687	9835344
1190704	67615086	295012355	46113362	12089502	72726712	10506993
1190705	74477892	264768060	40398903	10452966	65001420	9258356
1190707	123197830	207272598	28212297	7639324	50741487	6604237
1190722	84870304	345410621	53469790	14308232	84642443	11784147
1190731	78533236	288897882	42149930	11104720	70095553	9333535
1190740	65306474	252665069	35886915	9784492	61334093	8041849
1190742	91747538	261756119	39006185	10281188	63736248	8792766
1190746	69329398	279863034	45920616	12158030	68973983	10494432
1190747	81730244	280350264	44860416	11849406	69038925	10231330
1190749	59986842	327072252	45111435	11697190	79442292	9831492
1190753	121281546	252782055	37845848	9966678	60955837	8474026
1190771	69610630	290396608	41661060	10898758	70628687	9272330
1190777	89374982	284725387	43551726	11432508	69744137	9761870
1190784	109998660	257153397	39061188	10301716	63036350	8959266
1190788	69919998	217425997	31350630	8321834	52826692	7184316
1190811	53799996	703287260	108543539	40874466	183393441	23835828
1190827	57845512	721845146	113890259	44293654	189920353	25288035
CS	49938632	735256374	107010740	39865486	189546470	23279550

Supplemental Table S2. Mapping statistics for the 104 accessions from the core set of the Watkins bread wheat landrace collection plus Chinese Spring. Paired end sequencing reads aligned to the 13.4 Gbp wheat reference sequence.

Accession	Bisulfite conver-sion rate (%)	Depth of coverage chloro-plast	% coverage chloro-plast	Accession	Bisulfite conver-sion rate (%)	Depth of coverage chloro-plast	% coverage chloro-plast
1190007	99.1	327.0	63.4	1190440	99.0	430.8	61.6
1190032	99.0	293.6	76.8	1190451	99.1	280.0	87.0
1190034	99.0	371.8	80.9	1190460	98.7	393.1	39.0
1190040	99.1	297.8	66.5	1190468	98.9	348.0	65.8
1190042	99.1	361.1	65.4	1190471	98.7	355.7	50.8
1190044	99.1	247.5	79.2	1190474	98.7	269.3	59.7
1190045	97.8	481.9	56.3	1190475	99.1	622.2	92.0
1190079	98.1	357.5	57.5	1190481	97.7	647.8	44.9
1190092	98.4	365.7	61.4	1190483	98.2	536.9	49.6
1190103	98.8	404.8	98.4	1190496	98.7	413.1	47.2
1190110	98.5	337.8	69.0	1190507	98.5	326.8	51.9
1190126	97.7	537.7	48.5	1190546	98.5	381.2	64.1
1190127	98.5	361.7	48.5	1190551	99.0	341.6	76.7
1190139	99.0	301.0	77.8	1190560	99.1	304.6	78.7
1190141	98.8	432.3	98.8	1190562	99.1	335.4	68.5
1190145	98.9	270.0	64.6	1190579	99.0	363.2	71.8
1190149	98.7	330.3	54.6	1190580	98.9	364.2	63.6
1190160	99.1	315.5	75.9	1190591	98.9	249.2	61.0
1190166	99.0	340.0	57.9	1190624	97.7	737.1	64.0
1190181	98.4	601.5	69.7	1190627	98.7	382.5	98.2
1190199	98.5	599.8	60.7	1190629	97.8	644.3	53.1
1190209	98.4	627.0	70.9	1190637	98.6	497.8	72.4
1190216	98.7	464.0	70.4	1190639	98.9	392.1	73.4
1190218	98.4	480.7	52.4	1190651	98.7	469.2	58.2
1190219	98.0	470.7	74.8	1190652	98.5	420.8	75.9
1190223	98.8	405.3	63.4	1190662	99.2	347.3	71.7
1190224	98.4	305.4	50.2	1190670	99.1	323.2	69.1
1190231	98.7	264.5	56.2	1190671	99.0	289.1	60.3
1190238	98.8	391.9	98.0	1190683	98.8	303.9	52.6
1190239	98.8	232.3	68.2	1190685	99.0	360.0	62.1
1190246	98.6	390.0	46.3	1190690	99.0	358.6	76.6
1190254	98.9	256.8	65.4	1190694	98.4	382.7	61.6
1190264	97.7	654.3	53.1	1190698	97.7	620.5	38.8
1190273	97.4	458.3	51.7	1190700	98.6	354.2	76.7
1190281	97.9	469.3	67.7	1190704	98.6	311.0	63.2
1190291	98.6	454.9	59.9	1190705	98.6	328.3	66.7
1190292	98.9	493.6	98.9	1190707	98.3	527.9	48.7
1190299	98.9	309.4	78.9	1190722	99.1	494.0	60.9
1190300	98.3	418.5	44.9	1190731	98.9	407.9	53.0
1190308	98.7	498.0	99.1	1190740	99.0	368.2	59.1

1190313	98.3	599.0	55.8	1190742	99.1	384.4	74.9
1190324	99.2	398.9	91.8	1190746	98.9	262.3	74.4
1190325	98.7	380.6	53.9	1190747	98.8	296.1	72.5
1190349	98.9	230.7	54.0	1190749	98.9	445.4	66.5
1190352	98.7	300.4	58.4	1190753	98.8	569.1	58.4
1190355	98.7	378.4	54.5	1190771	98.8	389.8	67.3
1190360	99.1	330.9	62.5	1190777	98.6	414.0	50.7
1190387	98.1	608.8	58.4	1190784	98.6	473.0	53.5
1190396	98.7	511.8	61.8	1190788	98.7	363.2	56.1
1190398	98.4	411.7	94.8	1190811	98.9	476.6	98.9
1190406	98.2	430.2	48.8	1190827	98.9	429.7	98.4
1190420	98.9	357.4	89.1	CS	98.8	500.8	99.3
1190433	98.4	433.4	75.5				

Supplemental Table S3. Bisulfite conversion statistics for the 104 lines from the core set of the Watkins bread wheat landrace collection plus Chinese Spring. Mapping statistics for the paired end sequencing reads aligned to the non-methylated chloroplast wheat reference sequence to determine bisulfite conversion rates.

**Geographic region**	**Number of accessions in region**										
	Cluster 1	Cluster 2	Cluster 1a	Cluster 1b	Cluster 1c	Cluster 2a	Cluster 2b	Cluster 2c	Cluster 2d	Cluster 2e	Outlier
Western Europe	25	1	0	4	21	0	0	0	0	1	
Eastern Europe	18	1	5	1	12	1	0	0	0	0	
North/Sub-Saharan Africa	9	1	2	0	7	0	0	0	1	0	
Middle East	7	0	0	0	7	0	0	0	0	0	
South Central/South East Asia	13	17	5	1	7	0	10	7	0	0	
North East Asia	7	4	1	1	4	3	0	0	0	1	1
Australia	2	0	0	0	2	0	0	0	0	0	
**TOTAL**	**81**	**24**	**13**	**7**	**60**	**4**	**10**	**7**	**1**	**2**	**1**

Supplemental Table S4. Geographical accession origins combined with hierarchical cluster analysis on 104 accessions from the Watkins core collection plus Chinese Spring wheat for SNPs. Geographical positions of the accessions colour coded by their allocated cluster from Figure 1b after SNP hierarchical clustering**.**

Accession	% of methylated cytosines across CpG sites	% of methylated cytosines across CHG sites	% of methylated cytosines across CHH sites	Average methylation levels at CpG sites (%)	Average methylation levels at CHG sites (%)	Average methylation levels at CHH sites (%)
1190007	62.4	18.3	1.4	52.3482	10.0187	1.38997
1190032	59.7	19.7	1.5	48.6254	11.1144	1.95899
1190034	65.1	22	1.4	56.9242	15.3017	1.5516
1190040	59.9	18	1.3	50.6906	10.6596	1.35311
1190042	58.1	16.8	1.3	48.88	9.6202	1.28245
1190044	60	19.4	1.4	50.4025	11.818	1.28387
1190045	60.7	20.3	2.8	50.2212	12.111	3.56553
1190079	58.9	17.6	2.2	49.9362	10.9977	3.01493
1190092	61	18.7	1.9	52.9087	12.8084	2.85181
1190103	64.6	22.8	1.7	59.7386	20.0708	1.63412
1190110	57.4	17.7	1.8	47.7724	11.2052	2.82888
1190126	61.4	20.1	2.3	54.1384	14.4749	3.38895
1190127	61.9	20.4	1.5	52.6617	13.401	1.61081
1190139	58.6	16.7	1.4	49.8788	11.0279	1.59785
1190141	64.3	23.1	1.8	60.3007	20.9271	1.80224
1190145	58.6	18.4	1.4	49.395	11.4925	1.49169
1190149	58.3	18.2	1.5	48.539	10.6659	1.96721
1190160	63.6	19.1	1.3	54.5773	11.5183	1.5167
1190166	58.2	18	1.3	47.8539	10.0684	1.38056
1190181	62.4	21.7	1.9	55.1051	16.5056	3.19695
1190199	62.6	21.6	2	53.2542	14.1509	2.87567
1190209	62.6	19.6	2.3	54.972	14.4005	3.44357
1190216	62.4	19.3	1.9	54.0214	12.4523	2.17963
1190218	57.6	18	2	49.2645	11.7649	2.80703
1190219	61.1	20.4	2.6	52.1884	13.2625	3.15349
1190223	63.3	20.5	1.4	55.8874	15.3767	1.58775
1190224	59.9	19.1	1.6	51.1855	13.0894	1.79937
1190231	55.3	18.1	1.5	44.9866	10.8662	1.67572
1190238	63.9	22	1.6	60.0884	20.6549	1.55412
1190239	57.3	16.9	1.5	48.3816	10.8272	1.91798
1190246	59.4	17.6	1.5	50.2149	11.1384	1.78041
1190254	57.5	17.3	1.2	48.4022	10.4222	1.24784
1190264	64.8	21.7	1.9	57.7582	18.0504	3.3926
1190273	57.9	18	2.3	50.5391	13.3748	3.40991
1190281	59.2	18.7	2.3	51.5497	14.1868	3.93599
1190291	59.3	19.3	2	50.121	12.3349	2.32051
1190292	65.4	23.5	1.8	60.2867	20.839	1.71362
1190299	61.7	18.6	1.4	52.8595	12.3602	1.61435
1190300	60.2	19	1.9	51.0578	12.051	2.58088
1190308	64.5	22.8	1.8	59.584	20.3885	1.7778
1190313	58	19.4	2.3	49.7758	13.2901	2.99512
1190324	65.8	20.7	1.4	58.0387	14.9867	1.52313
1190325	64	20.8	1.5	54.8952	13.1188	1.77225
1190349	61.8	19.3	1.3	51.7893	10.865	1.1626
1190352	60	19.5	1.4	50.0685	12.0239	2.03592
1190355	59	17.8	1.5	49.779	11.078	1.62284
1190360	59.5	17.4	1.2	50.298	10.3035	1.29902
1190387	62	21.2	2	52.5169	14.387	3.14956
1190396	61.8	20	1.6	52.8227	13.4234	2.23216
1190398	62.3	20.8	1.9	53.4928	14.0208	3.09706
1190406	60.9	20.2	2	51.6976	12.9305	2.51612
1190420	60.9	19	1.6	52.3527	12.4441	2.81278
1190433	61.9	17.8	2.1	54.5651	12.5891	3.0108
1190440	66.7	20.9	1.4	58.3069	14.5937	1.55644
1190451	61.7	19.1	1.4	52.5088	12.1595	1.5061

1190460	63.5	19.4	1.4	54.0217	10.716	1.57965
1190468	63.2	19.4	1.4	54.2499	12.5949	1.89984
1190471	60.9	16.2	1.4	51.7036	9.89855	1.55667
1190474	59.4	18.2	1.3	49.8723	10.9151	1.31184
1190475	69	22.8	1.6	62.2984	18.7097	2.59797
1190481	62.4	20.6	2.4	53.3035	13.7513	3.48196
1190483	60.6	18.8	1.8	52.3604	13.212	3.15503
1190496	60.1	17.3	1.4	51.8331	11.0797	1.83121
1190507	56.2	17.2	1.7	46.7452	10.5544	2.29829
1190546	60.6	17.9	2	52.5109	11.4425	2.75141
1190551	63.2	18.3	1.3	54.4556	11.4475	1.32376
1190560	63.5	19	1.2	54.5601	11.3608	1.24016
1190562	61.1	17.5	1.3	51.8332	10.1372	1.30397
1190579	60.7	19.9	1.5	51.1617	12.0326	1.48266
1190580	60.9	18.6	1.5	51.2179	11.0888	1.51877
1190591	61	19.8	1.6	50.7455	10.9875	1.30782
1190624	64.7	21.1	2.6	56.1512	15.0593	4.16469
1190627	64.4	24.3	1.8	59.6365	22.1985	1.78439
1190629	55	19.7	2.9	45.9455	13.4022	3.95513
1190637	60.8	19.8	1.9	52.3945	13.7661	2.99689
1190639	59.7	17.9	1.6	50.858	11.5322	1.92461
1190651	57.7	19.6	1.6	48.8911	12.9021	2.41615
1190652	61.4	18.3	2.1	53.6107	11.6186	2.59651
1190662	65.7	21.5	1.3	56.7423	13.5195	1.24572
1190670	62.7	18.7	1.3	53.9351	11.6058	1.4007
1190671	59.6	18.1	1.4	49.2086	10.0899	1.39275
1190683	60	18	1.3	50.3834	10.6376	1.31464
1190685	61.7	17.9	1.5	52.5313	10.5283	1.51216
1190690	57.4	17.2	1.5	47.6548	9.88814	1.48775
1190694	66.9	22.6	2.3	56.4992	12.6584	2.11879
1190698	63.9	22	2.7	53.4617	13.4704	3.11727
1190700	60.3	18.9	1.8	50.958	11.8885	2.43581
1190704	59.9	17.7	1.7	50.6512	11.2732	2.11427
1190705	58.6	17.4	1.9	49.4979	10.6608	2.20478
1190707	62.2	19.7	2.2	53.7897	12.9307	2.63103
1190722	65.6	19.8	1.3	56.9969	13.1299	1.54053
1190731	65.1	19.4	1.4	56.8183	11.8388	1.6703
1190740	66.5	21.2	1.4	56.8392	12.4115	1.4042
1190742	63	17.3	1.2	54.9714	10.921	1.61915
1190746	56.8	16.1	1.3	48.388	10.0604	1.41558
1190747	58.5	17.4	1.3	50.3138	11.2645	1.85386
1190749	64.9	21.4	1.5	55.6296	12.7219	1.73164
1190753	61.5	18.8	1.6	53.3241	12.139	1.98668
1190771	62.1	18.1	1.7	52.6043	10.558	1.99465
1190777	61.5	18.3	1.6	52.7804	11.6659	2.15545
1190784	60	17.8	1.7	51.4841	11.2072	2.19702
1190788	59.8	19	1.7	49.8182	11.2258	1.55419
1190811	69.1	21.8	1.7	64.2645	19.3691	1.62891
1190827	66.5	23.7	1.7	62.4356	21.903	1.70654
CS	71.4	24.2	1.8	65.9617	20.8912	1.77139

Supplemental Table S5. Methylation levels for the 104 lines from the core set of the Watkins bread wheat landrace collection plus Chinese Spring. Detailing the percentages of methylated cytosines found at CpG, CHG and CHH sites and also the average methylation levels in the three sequence contexts.

	Sub-genome A number	Sub-genome A (%)	Sub-genome B number	Sub-genome B (%)	Sub-genome D number	Sub-genome D (%)	Unknown
Cytosines mapped (>= 10X)	257,312	30.1	255,461	29.9	298,676	35.0	42,483
	**CpG Number**	**CpG (%)**	**CHG Number**	**CHG (%)**	**CHH Number**	**CHH (%)**	**Unknown**
Cytosines mapped (>= 10X)	77,249	10.7	109,125	15.1	535,929	74.2	131,629
	**CpG Number**	**CpG (%)**	**CHG Number**	**CHG (%)**	**CHH Number**	**CHH (%)**	
Associated SMP sites (359,500 total)	18,965	5.3	61,666	17.2	278,869	77.5	

Supplemental Table S6. Detailing the 853,932 cytosine sites that were mapped to ≥10X in all 104 accessions plus Chinese Spring. Detailing the numbers of sites associated with each sub-genome of wheat, the proportions found at CpG, CHG and CHH sites and also the numbers identified as SMPs in the three sequence contexts.

Accession	Number of SMP sites	Number of SMP sites with CS	Number of SMP sites with CS (>50% difference)	Accession	Number of SMP sites	Number of SMP sites with CS	Number of SMP sites with CS (>50% difference)
1190007	19774	165441	8619	1190440	16712	166844	5831
1190032	23266	173884	10604	1190451	15335	166034	6151
1190034	15563	166636	5134	1190460	32742	169798	15891
1190040	16104	165582	6860	1190468	25489	173743	10004
1190042	20605	165060	9497	1190471	22333	167730	9911
1190044	13764	163097	6223	1190474	17808	163708	7072
1190045	54267	196265	27785	1190475	21164	183448	7636
1190079	37566	187760	17338	1190481	47484	194180	23235
1190092	39420	186826	17918	1190483	38893	190423	18170
1190103	11279	162092	3921	1190496	22862	172315	9408
1190110	35541	187153	17259	1190507	26982	179407	12745
1190126	59084	195819	29611	1190546	40119	184183	19298
1190127	15742	166268	5752	1190551	14597	164131	5840
1190139	12766	169501	5136	1190560	17412	163410	7451
1190141	13380	162662	3791	1190562	18425	164253	8391
1190145	15023	166587	5836	1190579	21324	165816	9587
1190149	26028	174210	11699	1190580	21370	167130	9765
1190160	22255	167535	10298	1190591	18776	163404	8218
1190166	19959	166566	9342	1190624	60507	203496	30827
1190181	32621	189695	14173	1190627	11867	162594	3898
1190199	43028	186194	20566	1190629	64659	202448	33059
1190209	54104	194376	26523	1190637	41554	188655	20437
1190216	32657	176669	15490	1190639	24745	173207	11092
1190218	43215	187268	21216	1190651	35726	182424	17483
1190219	56293	189334	29872	1190652	46367	183730	23280
1190223	13554	167379	4647	1190662	16471	161156	6341
1190224	16096	170246	5155	1190670	15900	164878	5862
1190231	16626	170594	7781	1190671	18377	164227	7688
1190238	11990	162700	3758	1190683	18375	164236	7889
1190239	19032	173151	6831	1190685	23892	167864	10754
1190246	20557	170817	8967	1190690	22843	167818	10985
1190254	15801	163449	6521	1190694	32547	172082	14484
1190264	30259	192585	12469	1190698	46352	186977	22622
1190273	39655	194365	17203	1190700	27534	178227	11523
1190281	48291	201703	22873	1190704	20395	175554	8367
1190291	36142	179874	17739	1190705	27716	175790	11569
1190292	12148	162065	4028	1190707	46009	182389	22231
1190299	15604	169163	5872	1190722	18916	167286	7493
1190300	36276	183287	17383	1190731	23798	168057	9052

1190308	12028	162804	4067	1190740	22632	165674	10083
1190313	55831	191099	28935	1190742	23056	169925	9624
1190324	14918	167107	5304	1190746	15323	166067	6138
1190325	19299	169540	7584	1190747	20680	172989	7927
1190349	16496	161444	7071	1190749	22894	170473	9793
1190352	25303	174814	10974	1190753	29086	173318	13475
1190355	20011	168144	8592	1190771	23830	172956	10672
1190360	18803	165273	8650	1190777	22195	174817	9292
1190387	37045	189891	17646	1190784	28478	176682	12959
1190396	23024	177397	10161	1190788	26265	167787	13113
1190398	38708	186391	18741	1190811	12580	161490	3853
1190406	37136	180644	16283	1190827	13597	161174	3408
1190420	37345	185992	17265	CS	14395	-	-
1190433	52200	190301	25742				

Supplemental Table S7. Single Methylation Polymorphisms (SMPs) for the 104 lines from the core set of the Watkins bread wheat landrace collection and Chinese Spring. Cytosine sites that were covered by at least ten reads were either called methylated (denoted as 100%) using our standard thresholds, or were called un-methylated (denoted as 0%) if < 1% of reads showed methylation, otherwise sites showing intermediate methylation levels were listed as heterozygous (denoted as 50%). Accession specific SMPs are here defined from the 359,500 global SMPs, firstly, if a accession showed 100% methylation, secondly, if a accession showed a different methylation status to Chinese Spring and thirdly, if the accession showed a >50% difference i.e. accession denoted as 0% methylated and Chinese Spring 100% methylated or vice versa.

Geographic region	Number of accessions in region									
	Cluster 1	Cluster 2	Cluster 1a	Cluster 1b	Cluster 2a	Cluster 2b	Cluster 2c	Cluster 2d	Cluster 2e	Cluster 2f
Western Europe	1	25	0	1	1	0	15	0	4	5
Eastern Europe	3	15	1	2	0	1	11	0	2	1
North/Sub-Saharan Africa	1	9	0	1	0	1	7	0	0	1
Middle East	1	6	0	1	0	0	5	0	1	0
South Central/South East Asia	6	25	1	5	2	13	6	1	3	0
North East Asia	0	11	0	0	1	0	5	0	3	2
Australia	0	2	0	0	0	0	2	0	0	0
**TOTAL**	**12**	**93**	**2**	**10**	**4**	**15**	**51**	**1**	**13**	**9**

Supplemental Table S8. Geographical accession origins combined with hierarchical cluster analysis on 104 accessions from the Watkins core collection plus Chinese Spring wheat. Geographical positions of the accessions colour coded by their allocated cluster from Figure 1d after CpG SMP hierarchical clustering**.**

Accession No.	Country of Origin	Region / Locality	Position	Elevation
1190460	Afghanistan			
1190468	Afghanistan			
1190471	Afghanistan			
1190474	Afghanistan			
1190475	Afghanistan			
1190742	Algeria			
1190007	Australia			
1190507	Australia			
1190349	Bulgaria	Varna, Voditza		
1190624	Bulgaria	Vetovo		
1190223	Burma	Ketku, E Jawnghwe State		
1190313	Burma	S. Shan States		
1190264	Canary Islands	Teneriffe		
1190141	China	Mukden		
1190224	China	Tsinan district		
1190324	China	Peking. Grown in city suburbs		
1190440	China			
1190651	China			
1190652	China			
1190698	China			
1190700	China			
1190722	China			
1190827	China	Shanghai (Tsao-Ka-Doo)		
1190639	Crete	Yerolakko, Canea		
1190291	Cyprus	Whole Island		
1190292	Cyprus	Paphos district.		
1190209	Egypt			
1190747	Ethiopia			
1190777	Finland			
1190040	France			
1190042	France			
1190110	France			
1190139	France			
1190281	Greece	Karditza & Trikkala		
1190560	Greece	Corfu Island		
1190562	Greece	Tripolis district, Peloponese		
1190690	Greece	Kalvarita district, Peloponese		
1190231	Hungary			
1190032	India	Sandhori, Nushki, Baluchistan	30N 66E	964
1190034	India	Etawah market, United Provinces	27N 79E	152
1190079	India	United Provinces		
1190092	India	Etawa, United Provinces	27N 79E	152
1190126	India	United Provinces		
1190127	India	Kurk, Sibi, Baluchistan	29N 68E	93
1190166	India	Saharanpur, United Provinces	30N 78E	314
1190199	India	Margnzani, Sibi, Baluchistan	29N 68E	93
1190246	India	Sadar Killa Saifulla, Baluchistan	32N 68E	2298
1190406	India	Hasanganj, United Provinces	27N 81E	125
1190420	India	Barchhamai, United Provinces	27N 79E	152
1190433	India	Musafirpur, Baluchistan	32N 68E	2298
1190694	India	Punjab	31N 74E	194
1190707	India	Panjpai, Baluchistan		
1190731	India	Sikkim	27N 89E	1108
1190238	Iran	Mehraban, N Hamadan	36N 48E	1480
1190308	Iran	Rasht, Caspian shore	38N 50E	-29
1190579	Iran	Hamadan	35N 48E	1812

1190580	Iran	Rasht, Caspian shore	38N 50E	-29
1190627	Iran	Asadabad, SW Hamadan	34N 48E	1745
1190629	Iran	Hashdarud, Azerbaijan province	40N 50E	-29
1190704	Iran	53 miles SE of Yazd, Mehdiabad	32N 52E	2048
1190705	Iran	Sauj Bolak, W of Tehran	36N 52E	3423
1190103	Italy			
1190784	Italy	Royal Exp. Sta. Rieti		
1190044	Morocco	Quebdana, Melilla	35N 3W	172
1190216	Morocco	Zenatas, Shaouia	34N 8W	0
1190254	Morocco	Tazi, Kenitra	34N 6W;	278
1190496	Morocco	Mazagan district		
1190398	Palestine			
1190181	Poland			
1190481	Poland			
1190483	Poland			
1190670	Poland			
1190396	Portugal	Golegã		
1190591	Portugal	Frontiera		
1190451	Romania			
1190662	Romania			
1190145	Spain	Navarre		
1190160	Spain	Navarre		
1190219	Spain	Vallodolid district		
1190239	Spain	Gallegos, Salamanca		
1190273	Spain	Valverde del Camino, Huelva	37N 6W	8
1190387	Spain	Minorca, southern part of Mahon	40N 4E	151
1190546	Spain	Navarre		
1190551	Spain	Navarre		
1190683	Spain	Navarre		
1190685	Spain	Campiñas, Seville	37N 6W	8
1190045	Syria	Lebanon, Bekaa, Damascus		
1190218	Tunisia	Soul-el-Khemis	37N 9E	88
1190811	Tunisia	Maison Carree		
1190299	Turkey	Seraikeuy		
1190300	Turkey	Kirk Agach, 50 miles NE of Izmir	39N 28E	1092
1190637	Turkey	Kirk Agach, 50 miles NE of Izmir	39N 28E	1092
1190149	UK			
1190325	UK			
1190671	USSR	Armenia		
1190740	USSR	Siberia		
1190746	USSR	Azerbaijan		
1190749	USSR	Siberia		
1190753	USSR	Don		
1190771	USSR	Irkutsk		
1190788	USSR	Turkestan		
1190352	Yugoslavia	Bugojno, Travnik, Bosnia	44N 18E	633
1190355	Yugoslavia	Bukovica, Dalmatia		
1190360	Yugoslavia	Veliko Hoce		

Supplemental Table S9. Geographical accession origins for 104 accessions from the Watkins core collection. Detailed geographical positions of the accessions outlining country of origin, region/locality, position and elevation where available**.**

Cluster	GO.ID	Term	Annotated	Significant	Expected	Topgo Fisher
Cluster 1a	GO:0009678	hydrogen-translocating pyrophosphatase activity	6	1	0.01	0.0064
	GO:0010309	acireductone dioxygenase [iron(II)-requiring]	7	1	0.01	0.0075
	GO:0004407	histone deacetylase activity	10	1	0.01	0.0107
	GO:0008889	glycerophosphodiester phosphodiesterase activity	13	1	0.01	0.0139
	GO:0004427	inorganic diphosphatase activity	16	1	0.02	0.0171
	GO:0015095	magnesium ion transmembrane transporter	16	1	0.02	0.0171
	GO:0016149	translation release factor activity	17	1	0.02	0.0181
	GO:0003723	RNA binding	703	4	0.76	0.0218
	GO:0004652	polynucleotide adenylyltransferase activity	21	1	0.02	0.0223
	GO:0005524	ATP binding	4629	10	4.97	0.0229
	GO:0008271	secondary active sulfate transmembrane transporter	22	1	0.02	0.0234
	GO:0008138	protein tyrosine/serine/threonine phosphate	24	1	0.03	0.0255
	GO:0005509	calcium ion binding	621	3	0.67	0.029
	GO:0005267	potassium channel activity	29	1	0.03	0.0307
	GO:0009982	pseudouridine synthase activity	33	1	0.04	0.0349
	GO:0004672	protein kinase activity	3014	7	3.24	0.0405
Cluster 1b	GO:0004489	methylenetetrahydrofolate reductase (NAD(P)H)	2	1	0	0.0027
	GO:0047334	6-phosphofructokinase (diphosphate) activity	5	1	0.01	0.0068
	GO:0003723	RNA binding	703	5	0.96	0.0070
	GO:0009678	hydrogen-translocating pyrophosphatase activity	6	1	0.01	0.0081
	GO:0004425	indole-3-glycerol-phosphate synthase	7	1	0.01	0.0095
	GO:0010309	acireductone dioxygenase [iron(II)-requiring]	7	1	0.01	0.0095
	GO:0030060	L-malate dehydrogenase activity	10	1	0.01	0.0135
	GO:0003872	6-phosphofructokinase activity	13	1	0.02	0.0175
	GO:0004427	inorganic diphosphatase activity	16	1	0.02	0.0216
	GO:0016149	translation release factor activity	17	1	0.02	0.0229
	GO:0004556	alpha-amylase activity	18	1	0.02	0.0242
	GO:0008271	secondary active sulfate transmembrane transporter	22	1	0.03	0.0295
	GO:0009982	pseudouridine synthase activity	33	1	0.04	0.044
	GO:0005524	ATP binding	4629	11	6.3	0.0456
Cluster 2a	GO:0016597	amino acid binding	53	1	0.01	0.0076
	GO:0020037	heme binding	1388	2	0.2	0.0151
	GO:0004497	monooxygenase binding	179	1	0.03	0.0254
Cluster 2b	GO:0004852	uroporphyrinogen-III synthase activity	4	1	0	0.0031
	GO:0009678	hydrogen-translocating pyrophosphatase activity	6	1	0	0.0046
	GO:0010309	acireductone dioxygenase [iron(II)-requiring]	7	1	0.01	0.0053
	GO:0005524	ATP binding	4629	9	3.54	0.0064
	GO:0016308	1-phosphatidylinositol-4-phosphate 5-kinase activity	10	1	0.01	0.0076
	GO:0004427	inorganic diphosphatase activity	16	1	0.01	0.0122
	GO:0008271	secondary active sulfate transmembrane transporter	22	1	0.02	0.0167
Cluster 2c	GO:0008271	Secondary active sulfate transmembrane transporter	22	1	0.01	0.011
	GO:0004672	protein kinase activity	3014	5	1.51	0.015
	GO:0046982	protein heterodimerization activity	376	2	0.19	0.015
	GO:0003746	translation elongation factor activity	45	1	0.02	0.022
	GO:0005524	ATP binding	4629	6	2.32	0.023
	GO:0016620	oxidoreductase activity	89	1	0.04	0.044
Cluster 2d	GO:0051060	pullulanase activity	2	1	0	0.0014
	GO:0004072	aspartate kinase activity	5	1	0	0.0036
	GO:0010309	acireductone dioxygenase [iron(II)-requiring]	7	1	0.01	0.005
	GO:0004425	indole-3-glycerol-phosphate synthase activity	7	1	0.01	0.005
	GO:0004435	phosphatidylinositol phospholipase C activity	9	1	0.01	0.0064
	GO:0004462	lactoylglutathione lyase activity	10	1	0.01	0.0071
	GO:0016151	nickel cation binding	12	1	0.01	0.0086
	GO:0046983	protein dimerization activity	1204	4	0.86	0.0197
	GO:0009982	pseudouridine synthase activity	33	1	0.02	0.0234
	GO:0016597	amino acid binding	53	1	0.04	0.0373
Cluster 2e	GO:0005524	ATP binding	4629	28	13.15	8.2e-05
	GO:0004672	protein kinase activity	3014	18	8.56	0.0022
	GO:0004399	histidinol dehydrogenase activity	1	1	0	0.0028
	GO:0003723	RNA binding	703	7	2	0.0056
	GO:0008236	serine-type peptidase activity	449	5	1.28	0.0094
	GO:0004852	uroporphyrinogen-III synthase activity	4	1	0.01	0.0113
	GO:0004733	pyridoxamine-phosphate oxidase activity	5	1	0.01	0.0141
	GO:0047334	diphosphate-fructose-6-phosphate	5	1	0.01	0.0141
	GO:0016887	ATPase activity	547	6	1.55	0.0161
	GO:0009678	hydrogen-translocating pyrophosphatase activity	6	1	0.02	0.0169
	GO:0003860	3-hydroxyisobutyryl-CoA hydrolase activity	7	1	0.02	0.0197
	GO:0010309	acireductone dioxygenase [iron(II)-requiring	7	1	0.02	0.0197
	GO:0004619	phosphoglycerate mutase activity	7	1	0.02	0.0197

	GO:0008964	phosphoenolpyruvate carboxylase activity	9	1	0.03	0.0253
	GO:0004407	histone deacetylase activity	10	1	0.03	0.0281
	GO:0004462	lactoylglutathione lyase activity	10	1	0.03	0.0281
	GO:0016151	nickel cation binding	12	1	0.03	0.0336
	GO:0003872	6-phosphofructokinase activity	13	1	0.04	0.0363
	GO:0005351	sugar:proton symporter activity	14	1	0.04	0.0391
	GO:0004427	inorganic diphosphatase activity	16	1	0.05	0.0445
	GO:0016149	translation release factor activity	17	1	0.05	0.0472
	GO:0004556	alpha-amylase activity	18	1	0.05	0.0499
Cluster 2f	GO:0004350	glutamate-5-semialdehyde dehydrogenase	1	1	0	0.0036
	GO:0004399	histidinol dehydrogenase activity	1	1	0	0.0036
	GO:0004349	glutamate 5-kinase activity	2	1	0	0.00072
	GO:0003887	DNA-directed DNA polymerase activity	28	1	0.01	0.00998
	GO:0004298	threonine-type endopeptidase activity	49	1	0.02	0.01741
	GO:0003723	RNA binding	703	2	0.25	0.02556
	GO:0051287	NAD binding	118	1	0.04	0.04144

Supplemental Table S10. Gene-set (GO) enrichment analysis. Analysis of the genes that are enriched within each of the 8 methylation based clusters defined in Figure 1d. This analysis was carried out using topGO ($p<0.05$, weight01 soring for Fishers exact test) and here, groups are colour coded by their allocated cluster from Figure 1d after CpG SMP hierarchical clustering**.**

Cluster 1a	
TRIAE_CS42_7BL_TGACv1_577494_AA1876920	Vacuolar proton pyrophosphatase
TRIAE_CS42_2AL_TGACv1_093955_AA0290160	1,2-dihydroxy-3-keto-5-methylthiopentene dioxygenase 4
TRIAE_CS42_2DL_TGACv1_158772_AA0525990	Histone deacetylase
TRIAE_CS42_4AL_TGACv1_290903_AA0990360	Glycerophosphodiester phosphodiesterase
TRIAE_CS42_7DL_TGACv1_602693_AA1965430	Non-imprinted in Prader-Willi/Angelman syndrome region protein
TRIAE_CS42_3B_TGACv1_226808_AA0819470	Eukaryotic peptide chain release factor subunit 1-2
TRIAE_CS42_2BS_TGACv1_146489_AA0466450	Pseudouridine synthase family protein
TRIAE_CS42_4AS_TGACv1_309150_AA1030480	evolutionarily conserved C-terminal region 2
TRIAE_CS42_7AL_TGACv1_557636_AA1784350	Poly(A) polymerase, Uncharacterized protein
TRIAE_CS42_1DL_TGACv1_062903_AA0221570	Protein kinase-like protein
TRIAE_CS42_2AL_TGACv1_096719_AA0320910	Chaperonin family protein
TRIAE_CS42_2DS_TGACv1_177910_AA0586990	Protein kinase-3
TRIAE_CS42_3AL_TGACv1_194659_AA0637340	protein, Putative wall-associated kinase 4, Uncharacterized protein
TRIAE_CS42_3AL_TGACv1_195208_AA0646470	Protein kinase-like
TRIAE_CS42_3AS_TGACv1_213124_AA0705560	Protein kinase superfamily protein
TRIAE_CS42_3DL_TGACv1_249981_AA0859840	Serine/threonine-protein kinase CTR1, Uncharacterized protein
TRIAE_CS42_5DL_TGACv1_433087_AA1401440	DNA-binding protein SMUBP-2, Uncharacterized protein
TRIAE_CS42_7AL_TGACv1_558004_AA1788850	protein, Putative CRK1 protein, Uncharacterized protein
TRIAE_CS42_7BL_TGACv1_576872_AA1857930	ABC transporter G family member 42
TRIAE_CS42_7AS_TGACv1_570504_AA1836730	Sulfate transporter
TRIAE_CS42_3AL_TGACv1_194119_AA0626810	Dual specificity phosphatase domain protein, Uncharacterized protein
TRIAE_CS42_4DL_TGACv1_342399_AA1112500	Calcineurin B-like protein 1, Uncharacterized protein
TRIAE_CS42_5AL_TGACv1_376562_AA1238490	Outward-rectifying potassium channel
Cluster 1b	
TRIAE_CS42_5AL_TGACv1_374680_AA1206310	Methylenetetrahydrofolate reductase
TRIAE_CS42_5BS_TGACv1_423221_AA1371470	Pyrophosphate--fructose 6-phosphate 1-phosphotransferase subunit alpha
TRIAE_CS42_1AL_TGACv1_000242_AA0007060	KH domain-containing protein, Uncharacterized protein
TRIAE_CS42_2BS_TGACv1_146489_AA0466450	Pseudouridine synthase family protein
TRIAE_CS42_3B_TGACv1_226808_AA0819470	Eukaryotic peptide chain release factor subunit 1-2,
TRIAE_CS42_5AL_TGACv1_376669_AA1239650	Ribonuclease
TRIAE_CS42_5DS_TGACv1_458282_AA1493070	Pumilio homology domain family member 4,
TRIAE_CS42_7BL_TGACv1_577494_AA1876920	Vacuolar proton pyrophosphatase
TRIAE_CS42_5BL_TGACv1_404243_AA1291930	Indole-3-glycerol phosphate synthase
TRIAE_CS42_2AL_TGACv1_093955_AA0290160	1,2-dihydroxy-3-keto-5-methylthiopentene dioxygenase 4,
TRIAE_CS42_7BL_TGACv1_578344_AA1893330	Malate dehydrogenase
TRIAE_CS42_2BL_TGACv1_129437_AA0383590	Alpha-amylase, Uncharacterized protein
TRIAE_CS42_7AS_TGACv1_570504_AA1836730	Sulfate transporter
TRIAE_CS42_2AL_TGACv1_096719_AA0320910	Chaperonin family protein
TRIAE_CS42_2AS_TGACv1_113028_AA0350020	ABC transporter C family member 4, Uncharacterized protein
TRIAE_CS42_2DL_TGACv1_160790_AA0554180	Protein kinase superfamily protein
TRIAE_CS42_2DS_TGACv1_177910_AA0586990	Protein kinase-3
TRIAE_CS42_3AL_TGACv1_194659_AA0637340	protein, Putative wall-associated kinase 4, Uncharacterized protein
TRIAE_CS42_3AS_TGACv1_213124_AA0705560	Protein kinase superfamily protein
TRIAE_CS42_3DL_TGACv1_249981_AA0859840	Serine/threonine-protein kinase CTR1, Uncharacterized protein
TRIAE_CS42_4DS_TGACv1_362084_AA1176570	Non-specific serine/threonine protein kinase
TRIAE_CS42_7AL_TGACv1_558004_AA1788850	protein, Putative CRK1 protein, Uncharacterized protein
TRIAE_CS42_7BL_TGACv1_576872_AA1857930	ABC transporter G family member 42
Cluster 2a	
TRIAE_CS42_1AS_TGACv1_019776_AA0071350	ACT domain-containing protein
TRIAE_CS42_1AS_TGACv1_020449_AA0077560	Peroxidase
TRIAE_CS42_2BL_TGACv1_129435_AA0383560	Cytochrome P450, putative
Cluster 2b	
TRIAE_CS42_4BL_TGACv1_322099_AA1068530	Uroporphyrinogen III synthase
TRIAE_CS42_7BL_TGACv1_577494_AA1876920	Vacuolar proton pyrophosphatase
TRIAE_CS42_2AL_TGACv1_093955_AA0290160	1,2-dihydroxy-3-keto-5-methylthiopentene dioxygenase 4, Uncharacterized protein
TRIAE_CS42_1DL_TGACv1_062903_AA0221570	Protein kinase-like protein
TRIAE_CS42_2AL_TGACv1_096719_AA0320910	Chaperonin family protein
TRIAE_CS42_2DS_TGACv1_177910_AA0586990	Protein kinase-3
TRIAE_CS42_3AS_TGACv1_213124_AA0705560	Protein kinase superfamily protein
TRIAE_CS42_3B_TGACv1_225488_AA0808970	Leucine-rich repeat receptor protein kinase EXS, Uncharacterized protein
TRIAE_CS42_3B_TGACv1_227074_AA0821270	ABC transporter B family member 4, Uncharacterized protein
TRIAE_CS42_3DL_TGACv1_249981_AA0859840	Serine/threonine-protein kinase CTR1, Uncharacterized protein
TRIAE_CS42_4AL_TGACv1_288682_AA0955510	P-loop containing nucleoside triphosphate hydrolases superfamily protein
TRIAE_CS42_7DL_TGACv1_604906_AA2002900	protein, Phosphatidylinositol-4-phosphate 5-kinase 4, putative, expressed
TRIAE_CS42_7AS_TGACv1_570504_AA1836730	Sulfate transporter
Cluster 2c	
TRIAE_CS42_7AS_TGACv1_570504_AA1836730	Sulfate transporter
TRIAE_CS42_2DS_TGACv1_177910_AA0586990	Protein kinase-3
TRIAE_CS42_3AS_TGACv1_213124_AA0705560	Protein kinase superfamily protein
TRIAE_CS42_3B_TGACv1_225488_AA0808970	Leucine-rich repeat receptor protein kinase EXS, Uncharacterized protein
TRIAE_CS42_3DL_TGACv1_249981_AA0859840	Serine/threonine-protein kinase CTR1, Uncharacterized protein
TRIAE_CS42_7AL_TGACv1_558004_AA1788850	protein, Putative CRK1 protein, Uncharacterized protein
TRIAE_CS42_1DS_TGACv1_080958_AA0256060	Nuclear transcription factor Y subunit C-4
TRIAE_CS42_7DL_TGACv1_603225_AA1978740	Nuclear transcription factor Y subunit C-2, Uncharacterized protein
TRIAE_CS42_4BS_TGACv1_330398_AA1107460	Elongation factor P, Uncharacterized protein
TRIAE_CS42_2AL_TGACv1_096719_AA0320910	Chaperonin family protein
TRIAE_CS42_1DL_TGACv1_062822_AA0220490	Aldehyde dehydrogenase family 2 member C4, Uncharacterized protein
TRIAE_CS42_2BL_TGACv1_129964_AA0400580	Histidinol dehydrogenase, chloroplastic
Cluster 2d	
TRIAE_CS42_7AS_TGACv1_570057_AA1829510	Pullulanase
TRIAE_CS42_5BL_TGACv1_404321_AA1295470	Aspartokinase-homoserine dehydrogenase
TRIAE_CS42_2AL_TGACv1_093955_AA0290160	1,2-dihydroxy-3-keto-5-methylthiopentene dioxygenase
TRIAE_CS42_5BL_TGACv1_404243_AA1291930	Indole-3-glycerol phosphate synthase
TRIAE_CS42_2DS_TGACv1_177840_AA0585650	Phosphoinositide phospholipase C
TRIAE_CS42_2DS_TGACv1_177189_AA0568340	Lactoylglutathione lyase
TRIAE_CS42_6BS_TGACv1_513249_AA1636060	Urease accessory protein D
TRIAE_CS42_1AS_TGACv1_019480_AA0067170	basic helix-loop-helix (bHLH) DNA-binding superfamily protein
TRIAE_CS42_1BS_TGACv1_049786_AA0161460	basic helix-loop-helix (bHLH) DNA-binding superfamily protein
TRIAE_CS42_1DS_TGACv1_080958_AA0256060	Nuclear transcription factor Y subunit C-4

TRIAE_CS42_3B_TGACv1_229892_AA0829260	Basic helix-loop-helix (BHLH) DNA-binding superfamily
TRIAE_CS42_2BS_TGACv1_146489_AA0466450	Pseudouridine synthase family protein
Cluster 2e	
TRIAE_CS42_1DL_TGACv1_062903_AA0221570	Protein kinase-like protein
TRIAE_CS42_1DL_TGACv1_064143_AA0232870	ABC transporter A family member 7, Uncharacterized protein
TRIAE_CS42_2AL_TGACv1_093085_AA0271620	Protein kinase superfamily protein
TRIAE_CS42_2AL_TGACv1_096429_AA0319110	Serine/threonine-protein kinase
TRIAE_CS42_2AL_TGACv1_096629_AA0320370	Serine/threonine-protein kinase
TRIAE_CS42_2AL_TGACv1_096719_AA0320910	Chaperonin family protein
TRIAE_CS42_2AS_TGACv1_113331_AA0354530	DNA repair protein rhp54, Uncharacterized protein
TRIAE_CS42_2DS_TGACv1_177910_AA0586990	Protein kinase-3
TRIAE_CS42_2DS_TGACv1_178440_AA0595650	DNA repair and recombination protein
TRIAE_CS42_3AL_TGACv1_194659_AA0637340	protein, Putative wall-associated kinase 4, Uncharacterized protein
TRIAE_CS42_3AS_TGACv1_213124_AA0705560	Protein kinase superfamily protein
TRIAE_CS42_3B_TGACv1_221085_AA0729780	Kinase family protein
TRIAE_CS42_3B_TGACv1_224189_AA0793610	chromatin remodeling 8
TRIAE_CS42_3B_TGACv1_224499_AA0797310	ABC transporter C family member 3, Uncharacterized protein
TRIAE_CS42_3B_TGACv1_225488_AA0808970	Leucine-rich repeat receptor protein kinase EXS, Uncharacterized protein
TRIAE_CS42_3B_TGACv1_227074_AA0821270	ABC transporter B family member 4, Uncharacterized protein
TRIAE_CS42_3DL_TGACv1_249981_AA0859840	Serine/threonine-protein kinase CTR1, Uncharacterized protein
TRIAE_CS42_4DS_TGACv1_362084_AA1176570	Non-specific serine/threonine protein kinase
TRIAE_CS42_5AL_TGACv1_375259_AA1218670	protein, Putative brassinosteroid insensitive 1,
TRIAE_CS42_5AS_TGACv1_394416_AA1280240	Kinase family protein
TRIAE_CS42_5BS_TGACv1_423221_AA1371470	Pyrophosphate--fructose 6-phosphate 1-phosphotransferase subunit alpha
TRIAE_CS42_5DL_TGACv1_433034_AA1399050	Disease resistance protein RPS5, Uncharacterized protein
TRIAE_CS42_5DL_TGACv1_436138_AA1458450	protein, Putative brassinosteroid insensitive 1, Uncharacterized protein
TRIAE_CS42_6BS_TGACv1_514490_AA1660470	Calcium-transporting ATPase
TRIAE_CS42_6DS_TGACv1_542449_AA1720560	Kinase family protein
TRIAE_CS42_7AL_TGACv1_558004_AA1788850	protein, Putative CRK1 protein, Uncharacterized protein
TRIAE_CS42_7AL_TGACv1_558250_AA1791610	Pleiotropic drug resistance ABC transporter
TRIAE_CS42_7AS_TGACv1_569282_AA1812400	Receptor-like protein kinase, Uncharacterized protein
TRIAE_CS42_2BL_TGACv1_129964_AA0400580	Histidinol dehydrogenase, chloroplastic
TRIAE_CS42_1AL_TGACv1_000242_AA0007060	KH domain-containing protein, Uncharacterized protein
TRIAE_CS42_2AS_TGACv1_113863_AA0361580	Eukaryotic translation initiation factor 4G, Uncharacterized protein
TRIAE_CS42_2BS_TGACv1_146489_AA0466450	Pseudouridine synthase family protein
TRIAE_CS42_3B_TGACv1_226808_AA0819470	Eukaryotic peptide chain release factor subunit 1-2,
TRIAE_CS42_5DS_TGACv1_458278_AA1493000	Poly(RC)-binding protein 1, Uncharacterized protein
TRIAE_CS42_5DS_TGACv1_458282_AA1493070	Pumilio homology domain family member 4, Uncharacterized protein
TRIAE_CS42_6DS_TGACv1_543940_AA1745470	50S ribosomal protein L21
TRIAE_CS42_1AL_TGACv1_000981_AA0023030	Serine carboxypeptidase S28 family protein
TRIAE_CS42_1DL_TGACv1_061634_AA0200610	Serine carboxypeptidase S28 family protein
TRIAE_CS42_2BL_TGACv1_129333_AA0379190	Protease Do-like 8, chloroplastic, Uncharacterized protein
TRIAE_CS42_3B_TGACv1_222480_AA0765510	Subtilisin-like protease, Uncharacterized protein
TRIAE_CS42_7DS_TGACv1_624169_AA2059440	Serine carboxypeptidase-like protein 9, Uncharacterized protein
TRIAE_CS42_4BL_TGACv1_322099_AA1068530	Uroporphyrinogen III synthase
TRIAE_CS42_6BL_TGACv1_501948_AA1621790	protein, Pyridoxamine 5'-phosphate oxidase, putative, expressed
TRIAE_CS42_4AS_TGACv1_306691_AA1012140	Copper-translocating P-type ATPase family protein, expressed
TRIAE_CS42_7BL_TGACv1_577494_AA1876920	Vacuolar proton pyrophosphatase
TRIAE_CS42_1AL_TGACv1_000453_AA0012380	3-hydroxyisobutyryl-CoA hydrolase, mitochondrial, Uncharacterized protein
TRIAE_CS42_2AL_TGACv1_093955_AA0290160	1,2-dihydroxy-3-keto-5-methylthiopentene dioxygenase 4,
TRIAE_CS42_6DL_TGACv1_526666_AA1689390	phosphoglycerate/bisphosphoglycerate mutase
TRIAE_CS42_5BL_TGACv1_407230_AA1354660	Phosphoenolpyruvate carboxylase 2, Uncharacterized protein
TRIAE_CS42_6DS_TGACv1_543680_AA1743020	Histone deacetylase
TRIAE_CS42_2AS_TGACv1_114009_AA0363380	Lactoylglutathione lyase
TRIAE_CS42_6BS_TGACv1_513249_AA1636060	Urease accessory protein D
TRIAE_CS42_6DL_TGACv1_526713_AA1690350	Nucleotide-sugar transporter family protein
TRIAE_CS42_2BL_TGACv1_129437_AA0383590	Alpha-amylase, Uncharacterized protein
Cluster 2f	
TRIAE_CS42_3DL_TGACv1_250063_AA0861520	Delta-1-pyrroline-5-carboxylate synthetase, Uncharacterized protein
TRIAE_CS42_2BL_TGACv1_129964_AA0400580	Histidinol dehydrogenase, chloroplastic
TRIAE_CS42_1AS_TGACv1_019404_AA0066210	DNA polymerase
TRIAE_CS42_5AS_TGACv1_393252_AA1270380	Proteasome subunit alpha type-5
TRIAE_CS42_4AS_TGACv1_309150_AA1030480	evolutionarily conserved C-terminal region 2
TRIAE_CS42_5DS_TGACv1_458282_AA1493070	Pumilio homology domain family member 4, Uncharacterized protein

Supplemental Table S11. Genes encompassing enriched GO terms from gene-set (GO) enrichment analysis. Analysis of the genes that are enriched within each of the 8 methylation based clusters defined in Figure 1d was detailed in Table S10. Here, for each cluster from Figure 1d after CpG SMP hierarchical clustering the genes encompassing the enriched GO terms are detailed**.**

Accession	Tri-genome sites	Uni-genome A sites	Uni-genome B sites	Uni-genome D sites	Bi-genome BD sites	Bi-genome AD sites	Bi-genome AB sites
1190007	16537	2155	2240	1980	2178	2228	1910
1190032	15984	2104	2304	1826	2090	2068	1816
1190034	32664	4496	4816	4240	4543	4346	3823
1190040	20541	2883	3221	2804	2904	2898	2520
1190042	14481	2052	2238	1897	2133	1973	1858
1190044	23293	3729	4126	3394	3392	3479	3087
1190045	16268	1862	2039	1734	1913	2029	1755
1190079	20372	2425	2580	2282	2510	2548	2314
1190092	18201	2191	2468	2117	2367	2353	2079
1190103	48275	6926	7483	6538	6415	6757	5629
1190110	17406	2093	2394	1832	2167	2055	2026
1190126	13895	1546	1777	1493	1702	1702	1446
1190127	26557	3615	4077	3276	3621	3577	3030
1190139	26444	3594	4113	3469	3540	3685	3202
1190141	52378	7185	8190	6842	6859	7152	6140
1190145	23167	2980	3378	2928	3150	3108	2621
1190149	16138	2031	2268	1996	2233	2141	1851
1190160	14356	1832	1944	1649	1845	1953	1579
1190166	14861	1978	2276	1869	2006	1999	1743
1190181	28382	3701	4041	3293	3688	3645	3373
1190199	15619	1760	1937	1673	1844	1793	1595
1190209	17077	1988	2237	1799	2148	1991	1798
1190216	14800	1688	1885	1639	1872	1713	1571
1190218	12245	1312	1509	1217	1296	1293	1135
1190219	8072	964	1037	928	993	998	867
1190223	34601	4815	5411	4475	4741	4613	3962
1190224	28186	3982	4328	3554	3659	3755	3184
1190231	17916	2818	3105	2574	2538	2753	2417
1190238	54245	7671	8288	7338	7212	7643	6233
1190239	25273	3446	3954	3145	3628	3378	3049
1190246	17517	2172	2495	2031	2167	2189	1815
1190254	18881	2509	2838	2323	2488	2390	2165
1190264	34879	4477	4932	3929	4475	4417	3920
1190273	28496	3249	3772	2905	3350	3389	3038
1190281	25609	2802	3073	2368	3001	2953	2597
1190291	13004	1719	1870	1599	1706	1692	1435
1190292	49562	6965	7647	6753	6957	6840	5444
1190299	28075	3968	4542	3705	4094	3945	3271
1190300	15278	1877	2165	1881	2187	2001	1615
1190308	50496	7377	8335	7026	6963	7091	6041
1190313	8179	994	1239	991	1092	1080	872
1190324	38092	5439	6219	4907	5140	5294	4800
1190325	24130	3187	3677	2918	3208	3340	2999
1190349	18290	2491	3007	2393	2570	2468	2178
1190352	16802	2286	2564	2226	2290	2229	2024
1190355	18655	2465	2849	2291	2411	2491	2174
1190360	15177	1999	2420	1945	2131	1993	1898
1190387	23265	2965	3356	2721	3021	2966	2591
1190396	22037	2843	3108	2677	2920	2863	2494
1190398	16622	2102	2265	1898	2169	2229	1872
1190406	17238	2092	2384	1850	2194	2089	1921
1190420	17379	2015	2340	1949	2243	2133	1925
1190433	13305	1487	1712	1448	1557	1575	1371
1190440	31614	4066	4920	4071	4471	4256	3543
1190451	24999	3415	3736	3203	3473	3612	2924
1190460	6005	820	886	761	801	781	679
1190468	21170	2660	2962	2537	2736	2745	2422
1190471	15172	1786	2142	1725	1802	1799	1594
1190474	18240	2294	2551	2123	2247	2231	1951
1190475	41046	5401	5868	5096	5226	5300	4497
1190481	19749	2189	2449	2157	2491	2502	2092
1190483	20275	2419	2747	2141	2469	2535	2303
1190496	20038	2668	2863	2410	2694	2596	2302
1190507	15837	1963	2258	1927	2092	1920	1699
1190546	14520	1672	1788	1467	1679	1745	1506
1190551	26139	3560	4021	3447	3545	3543	3017
1190560	21347	2988	3210	2628	2961	2951	2480
1190562	16961	2321	2562	2247	2395	2395	1962
1190579	16014	2259	2409	2112	2205	2148	1851
1190580	16259	2119	2530	2101	2261	2101	1768
1190591	16507	2255	2424	1961	2402	2238	1969
1190624	20826	2256	2512	2133	2402	2484	2098

1190627	50847	7298	8430	6909	6989	7068	6122
1190629	14259	1572	1791	1444	1503	1569	1350
1190637	15300	1818	2147	1709	1970	1960	1753
1190639	16894	2136	2480	2091	2292	2129	1937
1190651	10870	1351	1485	1284	1453	1334	1182
1190652	11131	1334	1544	1321	1476	1384	1198
1190662	25308	3540	3955	3267	3469	3501	3011
1190670	24367	3092	3638	3124	3507	3120	2500
1190671	16536	2229	2522	2125	2285	2224	1956
1190683	16174	2104	2371	2082	2161	2269	1825
1190685	15319	1853	2077	1862	1979	2021	1659
1190690	14146	1713	1959	1650	1796	1704	1495
1190694	17254	2034	2275	1880	2190	2112	1862
1190698	16241	1955	2103	1884	2005	2074	1718
1190700	21161	2662	2966	2545	2773	2787	2440
1190704	22063	3095	3297	2766	3131	3116	2742
1190705	18509	2084	2445	2035	2062	2101	1705
1190707	10053	1168	1298	1078	1115	1147	1101
1190722	26538	3800	4116	3545	3828	3847	3209
1190731	21697	2971	3261	2680	2892	2991	2604
1190740	15001	1907	2091	1831	1951	2021	1691
1190742	18636	2343	2498	2165	2289	2335	2060
1190746	23028	3202	3575	2943	3102	3331	2871
1190747	22921	2881	3413	2716	3047	2941	2518
1190749	20035	2793	3074	2565	2771	2876	2375
1190753	14660	1916	2090	1849	1830	1979	1643
1190771	19184	2422	2679	2187	2536	2540	2159
1190777	21193	2784	2961	2481	2846	2774	2413
1190784	17651	2170	2501	2202	2411	2241	1999
1190788	12101	1804	2005	1681	1666	1641	1440
1190811	54666	7854	8622	7214	7377	7570	6627
1190827	59065	7542	8735	6931	7504	7504	6558
CS	57565	7261	8441	7091	7390	7509	6371

Supplemental Table S12. Describing uni-, bi- and tri-genome methylation across the 104 Watkins accessions plus Chinese Spring. Detailed are the identified number of tri-genome, bi-genome and uni-genome methylated sites per accession. We classified methylation as tri-genome (in all three sub-genomes) using standard thresholds, or uni-genome (in a single sub-genome) and bi-genome (in two sub-genomes) using the tool Methylkit to identify a minimum methylation difference of 50% ($q<0.01$) between sub-genomes combined with our standard thresholds.

Uni-Genome Methylation	A(33%)	B(36%)	D(31%)
Transcribed (%)	88.2	87.5	86.4
% CpG methylation	93.5	94.6	95.8
% CHH methylation	0.3	0.3	0.3
% CHG methylation	6.2	5.1	3.9
Not Transcribed (%)	11.8	12.5	13.6
% CpG methylation	76.6	76.5	74.6
% CHH methylation	0.9	0.5	0.5
% CHG methylation	22.5	23.0	24.9
**Bi-Genome Methylation**	**BD(35%)**	**AD(35%)**	**AB(30%)**
Transcribed (%)	89.4	88.1	89.5
% CpG methylation	99.4	99.0	98.9
% CHH methylation	0.0	0.0	0.0
% CHG methylation	0.6	1.0	1.1
Not Transcribed (%)	10.6	11.9	10.5
% CpG methylation	79.8	73.1	75.2
% CHH methylation	0.0	0.0	0.0
% CHG methylation	20.2	26.9	24.8
**Tri-genome Methylation**			
Transcribed (%)		88.4	
% CpG methylation		97.2	
% CHH methylation		0.6	
% CHG methylation		2.2	
Not Transcribed (%)		11.6	
% CpG methylation		55.0	
% CHH methylation		2.8	
% CHG methylation		42.2	

Supplemental Table S13. Summary of orientation of the methylation sites that were analyzed across the 104 accessions from the Watkins collection plus Chinese Spring. Breakdown of methylation into uni-, bi- and tri-genome sites; transcribed (exon/intron) and non-transcribed regions and finally CpG, CHH and CHG sites. Figures average across all analyzed accessions.

Methylation Site	Sites mapped min 105 accessions	% Sites methylated in min 105 accessions	% Sites methylated in min 90 accessions	% Sites methylated in min 5 accessions
**Tri-genome sites**	9000 (x3 genomes)	0.6	14.3	64.5
**Uni-genome A sites**	3666	0	1.36	42.6
**Uni-genome B sites**	4263	0	0.99	42.9
**Uni-genome D sites**	3840	0	1.25	42.9
**Bi-genome BD sites**	4538	0	1.04	42.6
**Bi-genome AD sites**	4514	0.04	1.09	41.9
**Bi-genome AB sites**	4346	0	0.76	38.7

Supplemental Table S14. Describing uni-, bi- and tri-genome methylation conservation across the 104 Watkins accessions plus Chinese Spring. Detailed are the identified number of tri-genome, bi-genome and uni-genome methylated sites after; collation of all sites identified in the study and filtering of only those sites mapped to a minimum of 10X per sample. The percentage of these sites that show methylation in >=105, >=90 and finally >=5 of the accessions are also shown.

GO Term	Annotated	Significant	Expected	Fisher p-value
GO:0006468	protein phosphorylation	1105	25	8.3e-19
GO:0006886	intracellular protein transport	259	19	2.2e-15
GO:0006355	regulation of transcription, DNA-depende...	944	24	8.2e-14
GO:0055114	oxidation-reduction process	1073	22	3.2e-11
GO:0016192	vesicle-mediated transport	215	11	3.3e-11
GO:0006508	proteolysis	651	17	4.8e-11
GO:0016310	phosphorylation	1431	36	2.5e-09
GO:0032259	methylation	325	13	3.9e-08
GO:0006396	RNA processing	386	17	3.6e-07
GO:0006413	translational initiation	101	6	4.1e-07

Supplemental Table S15. Gene-set (GO) enrichment analysis for Tri-genome methylated sites in at least 90% of accessions. This analysis was carried out using topGO with p-values calculated using the weight01 scoring for the Fisher's exact test**.** Here we show the top 10 most enriched biological processes ($p < 0.05$).

Accession	Number of CpG DMRs	Number of CHG DMRs	Number of CHH DMRs	Accession	Number of CpG DMRs	Number of CHG DMRs	Number of CHH DMRs
1190007	56	15	20	1190433	50	16	0
1190032	52	12	33	1190440	70	13	10
1190034	57	16	4	1190451	62	14	5
1190040	57	9	9	1190460	70	12	99
1190042	52	10	19	1190468	60	16	20
1190044	78	13	3	1190471	55	10	33
1190045	73	17	3	1190474	61	14	9
1190079	57	16	90	1190475	47	10	1
1190092	61	11	0	1190481	59	15	1
1190103	61	14	2	1190483	47	11	0
1190110	55	10	1	1190496	60	10	12
1190126	55	19	1	1190507	67	18	35
1190127	52	18	4	1190546	46	13	121
1190139	53	12	2	1190551	58	11	0
1190141	57	12	4	1190560	61	13	6
1190145	57	15	2	1190562	63	14	10
1190149	50	13	39	1190579	56	22	10
1190160	37	14	25	1190580	57	18	12
1190166	55	14	16	1190591	67	18	7
1190181	65	9	1	1190624	62	14	0
1190199	56	17	0	1190627	66	17	6
1190209	53	15	0	1190629	59	16	0
1190216	52	14	1	1190637	59	11	1
1190218	42	12	0	1190639	70	12	24
1190219	55	17	2	1190651	57	12	1
1190223	52	13	6	1190652	69	10	3
1190224	49	9	11	1190662	56	12	3
1190231	86	14	8	1190670	61	11	8
1190238	55	16	1	1190671	45	12	13
1190239	54	10	15	1190683	53	13	4
1190246	60	17	25	1190685	60	13	21
1190254	47	17	4	1190690	54	13	15
1190264	49	11	1	1190694	58	20	28
1190273	58	12	1	1190698	60	14	1
1190281	51	8	168	1190700	59	9	37
1190291	61	13	75	1190704	57	15	18
1190292	59	12	3	1190705	49	10	32
1190299	56	14	3	1190707	50	12	150
1190300	54	11	89	1190722	66	11	12
1190308	89	15	8	1190731	69	10	26
1190313	53	13	1	1190740	65	14	20
1190324	66	13	7	1190742	65	15	25
1190325	66	12	7	1190746	58	11	2
1190349	63	13	6	1190747	52	18	1
1190352	58	10	0	1190749	55	20	25
1190355	56	12	7	1190753	58	10	46
1190360	64	14	0	1190771	71	15	21
1190387	52	13	127	1190777	65	15	26
1190396	55	9	26	1190784	62	11	38
1190398	56	12	110	1190788	74	23	22
1190406	53	17	75	1190811	59	11	1
1190420	75	15	1	1190827	66	10	1

Supplemental Table S16. Number of DMRs identified for each of the 104 Watkins accessions. DMRs in the CpG, CHG and CHH context for each accession when compared to Chinese Spring wheat.

GO.ID	Term	Annotated	Significant	Expected	topgoFisher
GO:0004739	pyruvate dehydrogenase (acetyl-transferase)	8	3	0.04	9.30E-06
GO:0004427	inorganic diphosphatase activity	16	3	0.09	9.00E-05
GO:0003746	translation elongation factor activity	45	4	0.25	0.00012
GO:0004396	hexokinase activity	20	3	0.11	0.00018
GO:0005536	glucose binding	20	3	0.11	0.00018
GO:0004764	shikimate 3-dehydrogenase (NADP+) activity	4	2	0.02	0.00018
GO:0003855	3-dehydroquinate dehydratase activity	4	2	0.02	0.00018
GO:0009678	hydrogen-translocating pyrophosphatase activity	6	2	0.03	0.00046
GO:0046556	alpha-L-arabinofuranosidase activity	7	2	0.04	0.00064
GO:0000166	nucleotide binding	7432	59	41.34	0.00109
GO:0051539	4 iron, 4 sulfur cluster binding	38	3	0.21	0.00124
GO:0019904	protein domain specific binding	11	2	0.06	0.00164
GO:0016818	hydrolase activity	1027	15	5.71	0.00216
GO:0000287	magnesium ion binding	247	6	1.37	0.00271
GO:0009540	zeaxanthin epoxidase [overall] activity	1	1	0.01	0.00556
GO:0035299	inositol pentakisphosphate 2-kinase activity	1	1	0.01	0.00556
GO:0004077	biotin-[acetyl-CoA-carboxylase] ligase activity	1	1	0.01	0.00556
GO:0004583	dolichyl-phosphate-glucose-glycolipid alpha glucosyltransferase activity	1	1	0.01	0.00556
GO:0008601	protein phosphatase type 2A regulator	23	2	0.13	0.00722
GO:0003723	RNA binding	703	13	3.91	0.00778

Supplemental Table S17. Most significant GO terms from topGO GSEA of molecular functions from within highly targeted gene families. Summary of topGO gene set enrichment from the "within accessions analysis" of highly targeted gene families ($p < 0.01$, weight01 GO scoring for Fisher's exact test).

GO.ID	Term	Annotated	Significant	Expected	topgoFisher
GO:0004396	hexokinase activity	20	3	0.04	8.00E-06
GO:0005536	glucose binding	20	3	0.04	8.00E-06
GO:0004739	pyruvate dehydrogenase (acetyl-transferase)	8	2	0.02	0.00011
GO:0005267	potassium channel activity	29	2	0.06	0.00149
GO:0003950	NAD+ ADP-ribosyltransferase activity	33	2	0.06	0.00192
GO:0035299	inositol pentakisphosphate 2-kinase activity	1	1	0	0.00196
GO:0009540	zeaxanthin epoxidase [overall] activity	1	1	0	0.00196
GO:0003746	translation elongation factor activity	45	2	0.09	0.00355
GO:0004073	aspartate-semialdehyde dehydrogenase activity	2	1	0	0.00391
GO:0004798	thymidylate kinase activity	2	1	0	0.00391
GO:0008265	Mo-molybdopterin cofactor sulfurase activity	2	1	0	0.00391
GO:0008942	nitrite reductase [NAD(P)H] activity	2	1	0	0.00391
GO:0051060	pullulanase activity	2	1	0	0.00391
GO:0019829	cation-transporting ATPase activity	64	2	0.13	0.00705
GO:008565	protein transporter activity	64	2	0.13	0.00705
GO:0003896	DNA primase activity	4	1	0.01	0.00781
GO:0004749	ribose phosphate diphosphokinase activity	5	1	0.01	0.00975
GO:0003942	N-acetyl-gamma-glutamyl-phosphate reductase	5	1	0.01	0.00975
GO:0019789	SUMO transferase activity	5	1	0.01	0.00975

Supplemental Table S18. Most significant GO terms from topGO GSEA of molecular functions from between highly targeted gene families. Summary of topGO gene set enrichment from the "between accessions analysis" of highly targeted gene families ($p < 0.01$, weight01 GO scoring for Fisher's exact test).

GO.ID	Term	Annotated	Significant	Expected	topgoFisher
GO:0000166	nucleotide binding	7432	114	105.39	5.10E-06
GO:0030785	N-methyltransferase activity	2	2	0.03	0.0002
GO:0008176	tRNA (guanine-N7-)-methyltransferase activity	3	2	0.04	0.0006
GO:0009039	urease activity	3	2	0.04	0.0006
GO:0004605	phosphatidate cytidylyltransferase activity	3	2	0.04	0.0006
GO:0009882	blue light photoreceptor activity	4	2	0.06	0.0012
GO:0004488	methylenetetrahydrofolate dehydrogenase activity	4	2	0.06	0.0012
GO:0051287	NAD binding	118	7	1.67	0.0015
GO:0016157	sucrose synthase activity	17	3	0.24	0.0017
GO:0003983	UTP:glucose-1-phosphate uridylyltransferase	5	2	0.07	0.002
GO:0004020	adenylylsulfate kinase activity	6	2	0.09	0.0029
GO:0004654	polyribonucleotide nucleotidyltransferase	6	2	0.09	0.0029
GO:0004351	glutamate decarboxylase activity	9	2	0.13	0.0068
GO:0042803	protein homodimerization activity	10	2	0.14	0.0084
GO:0004519	endonuclease activity	183	6	2.6	0.0086

Supplemental Table S19. Most significant GO terms from topGO GSEA of molecular functions from within low level targeted gene families. Summary of topGO gene set enrichment from the "within accessions analysis" of less targeted gene families ($p < 0.01$, weight01 GO scoring for Fisher's exact test).

GO.ID	Term	Annotated	Significant	Expected	topgoFisher
GO:0051287	NAD binding	118	8	1	4.80E-05
GO:0030785	N-methyltransferase activity	2	2	0.02	7.10E-05
GO:0009039	urease activity	3	2	0.03	0.00021
GO:0008176	tRNA (guanine-N7-)-methyltransferase activity	3	2	0.03	0.00021
GO:0016157	sucrose synthase activity	17	3	0.14	0.00037
GO:0000166	nucleotide binding	7432	78	62.81	0.0004
GO:0009882	blue light photoreceptor activity	4	2	0.03	0.00042
GO:0003855	3-dehydroquinate dehydratase activity	4	2	0.03	0.00042
GO:0004764	shikimate 3-dehydrogenase (NADP+) activity	4	2	0.03	0.00042
GO:0003983	UTP:glucose-1-phosphate uridylyltransferase	5	2	0.04	0.0007
GO:0004654	polyribonucleotide nucleotidyltransferase	6	2	0.05	0.00104
GO:0009678	hydrogen-translocating pyrophosphatase activity	6	2	0.05	0.00104
GO:0046556	alpha-L-arabinofuranosidase activity	7	2	0.06	0.00145
GO:0004518	nuclease activity	801	8	6.77	0.00152
GO:0004351	glutamate decarboxylase activity	9	2	0.08	0.00247
GO:0042803	protein homodimerization activity	10	2	0.08	0.00306
GO:0003723	RNA binding	703	14	5.94	0.0034
GO:0042578	phosphoric ester hydrolase activity	411	14	3.47	0.00355
GO:0019904	protein domain specific binding	11	2	0.09	0.00372
GO:0051539	4 iron, 4 sulfur cluster binding	38	3	0.32	0.00406
GO:0016151	nickel cation binding	12	2	0.1	0.00444
GO:0016818	hydrolase activity	1027	20	8.68	0.00495
GO:0008017	microtubule binding	135	5	1.14	0.00594
GO:0004722	protein serine/threonine phosphatase activity	136	5	1.15	0.00613
GO:0004519	endonuclease activity	183	4	1.55	0.00738
GO:0004427	inorganic diphosphatase activity	16	2	0.14	0.0079
GO:0004583	dolichyl-phosphate-glucose-glycolipid alpha	1	1	0.01	0.00845
GO:0004077	biotin-[acetyl-CoA-carboxylase] ligase	1	1	0.01	0.00845
GO:0004124	cysteine synthase activity	17	2	0.14	0.00891

Supplemental Table S20. Most significant GO terms from topGO GSEA of molecular functions from between low level targeted gene families. Summary of topGO gene set enrichment from the "between accessions analysis" of less targeted gene families ($p < 0.01$, weight01 GO scoring for Fisher's exact test).

Group	GO.ID	Term	Annotated	Significant	Expected	Topgo Fisher
High	GO:0000166	nucleotide binding	7432	71	47.37	5.4e-05
	GO:0004396	hexokinase activity	20	3	0.13	0.00027
	GO:0005536	glucose binding	20	3	0.13	0.00027
	GO:0004019	adenylosuccinate synthase activity	6	2	0.04	0.00060
	GO:0019901	protein kinase binding	27	3	0.17	0.00067
	GO:0003723	RNA binding	703	16	4.48	0.00125
	GO:0008889	glycerophosphodiester phosphodiesterase activity	13	2	0.08	0.00301
	GO:0031369	translation initiation factor binding	13	2	0.08	0.00301
	GO:0043015	gamma-tubulin binding	13	2	0.08	0.00301
	GO:0003995	acyl-CoA dehydrogenase activity	13	2	0.08	0.00301
	GO:0005247	voltage-gated chloride channel activity	18	2	0.11	0.00579
	GO:0008422	beta-glucosidase activity	1	1	0.01	0.00637
	GO:0008022	protein C-terminus binding	1	1	0.01	0.00637
	GO:0008734	L-aspartate oxidase activity	1	1	0.01	0.00637
	GO:0008601	protein phosphatase type 2A regulator activity	23	2	0.15	0.00938
Medium	GO:0003697	single-stranded DNA binding	26	5	0.18	2.1e-05
	GO:0033743	peptide-methionine (R)-S-oxide reductase activity	13	3	0.09	8.9e-05
	GO:0000155	phosphorelay sensor kinase activity	30	3	0.21	0.0012
	GO:0004824	lysine-tRNA ligase activity	8	2	0.06	0.0013
	GO:0051539	4 iron, 4 sulfur cluster binding	38	3	0.26	0.0023
	GO:0004563	beta-N-acetylhexosaminidase activity	11	2	0.08	0.0025
	GO:0005524	ATP binding	4629	47	32.05	0.0048
	GO:0004124	cysteine synthase activity	17	2	0.12	0.0061
	GO:0009540	zeaxanthin epoxidase [overall] activity	1	1	0.01	0.0069
	GO:0050515	4-(cytidine 5'-diphospho)-2-C-methyl-D-e	1	1	0.01	0.0069
Low	GO:0003855	3-dehydroquinate dehydratase activity	4	2	0.02	9.2e-05
	GO:0004764	shikimate 3-dehydrogenase (NADP+) activity	4	2	0.02	9.2e-05
	GO:0004185	serine-type carboxypeptidase activity	144	5	0.57	0.0028
	GO:0005452	inorganic anion exchanger activity	7	2	0.03	0.00032
	GO:0004107	chorismate synthase activity	1	1	0	0.00394
	GO:0002161	aminoacyl-tRNA editing activity	24	2	0.09	0.00402
	GO:0009013	succinate-semialdehyde dehydrogenase	2	1	0.01	0.00786
	GO:0004644	phosphoribosylglycinamide	2	1	0.01	0.00786
	GO:0004479	formyltransferasemethionyl-tRNA formyltransferase	2	1	0.01	0.00786
	GO:0030785	ribulose-bisphosphate carboxylase	2	1	0.01	0.00786

Supplemental Table S21. GO enrichment analysis of genes methylated with varying frequency across the 104 accessions. Summary of topGO gene set enrichment in the 3 groups (low, medium and high) where, for the high group, the gene targeted by CpG methylation appears in ≥90 accessions, for medium it appears in 40 to 90 accessions and for low it appears in <40 accessions.

Phenotype	Max	Origin	Value	Rank	Min	Origin	Value	Rank
height	1190141	China	125.5cm	803/811	1190292	Cyprus	60.75	5/811
	1190352	Yugoslavia	137.5cm	809/811	1190398	Palestine	70	13/811
heading date	1190209	Egypt	79d 3-Jun	7/817	1190481	Poland	118d 12-Jul	802/807
	1190034	India	79d 3-Jun	6/817	1190181	Poland	117.75 11-Jul	801/807
TGW	1190103	Italy	49.6g	781/784	1190308	Iran	21.4g	5/784
	1190181	Poland	44.17g	731/784	1190777	Finland	20.8g	2/784
grain width	1190181	Poland	3.9mm	780/784	1190299	Turkey	2.9mm	2/784
	1190103	Italy	3.8mm	777/784	1190777	Finland	2.9mm	1/784

Supplemental Table S22. 12 accessions selected for RNA-seq analysis. Phenotypic information for the 12 Watkins accessions that represented phenotypic tails for height, heading date, thousand-grain weight and grain width.

Accession 1	Accession 2	CHG DMRs	CHH DMRs	CpG DMRs
1190034-India	1190103-Italy	11	5	44
1190034-India	1190141-China	12	6	57
1190034-India	1190181-Poland	15	89	62
1190034-India	1190209-Egypt	13	1	42
1190034-India	1190292-Cyprus	15	6	50
1190034-India	1190299-Turkey	21	6	59
1190034-India	1190308-Iran	16	6	76
1190034-India	1190352-Yugoslavia	13	1	65
1190034-India	1190398-Palestine	12	132	47
1190034-India	1190481-Poland	16	1	49
1190034-India	1190777-Finland	19	31	63
1190103-Italy	1190141-China	6	3	52
1190103-Italy	1190181-Poland	9	86	49
1190103-Italy	1190209-Egypt	15	1	54
1190103-Italy	1190292-Cyprus	9	2	57
1190103-Italy	1190299-Turkey	12	3	51
1190103-Italy	1190308-Iran	15	3	79
1190103-Italy	1190352-Yugoslavia	10	56	61
1190103-Italy	1190398-Palestine	6	118	53
1190103-Italy	1190481-Poland	13	1	43
1190103-Italy	1190777-Finland	11	30	45
1190141-China	1190181-Poland	5	87	51
1190141-China	1190209-Egypt	14	1	56
1190141-China	1190292-Cyprus	8	4	65
1190141-China	1190299-Turkey	7	3	47
1190141-China	1190308-Iran	10	4	109
1190141-China	1190352-Yugoslavia	6	56	61
1190141-China	1190398-Palestine	4	127	60
1190141-China	1190481-Poland	9	1	52
1190141-China	1190777-Finland	8	28	59
1190181-Poland	1190209-Egypt	13	1	60
1190181-Poland	1190292-Cyprus	8	2	52
1190181-Poland	1190299-Turkey	10	84	45
1190181-Poland	1190308-Iran	8	88	103
1190181-Poland	1190352-Yugoslavia	8	1	59
1190181-Poland	1190398-Palestine	7	1	50
1190181-Poland	1190481-Poland	4	1	36
1190181-Poland	1190777-Finland	9	1	47
1190209-Egypt	1190292-Cyprus	16	1	54
1190209-Egypt	1190299-Turkey	21	1	55
1190209-Egypt	1190308-Iran	12	1	78
1190209-Egypt	1190352-Yugoslavia	13	1	61

1190209-Egypt	1190398-Palestine	13	226	60
1190209-Egypt	1190481-Poland	14	1	52
1190209-Egypt	1190777-Finland	13	2	58
1190292-Cyprus	1190299-Turkey	14	5	49
1190292-Cyprus	1190308-Iran	13	3	74
1190292-Cyprus	1190352-Yugoslavia	13	58	54
1190292-Cyprus	1190398-Palestine	10	115	46
1190292-Cyprus	1190481-Poland	10	2	60
1190292-Cyprus	1190777-Finland	11	28	53
1190299-Turkey	1190308-Iran	13	4	87
1190299-Turkey	1190352-Yugoslavia	10	1	57
1190299-Turkey	1190398-Palestine	10	107	48
1190299-Turkey	1190481-Poland	13	1	51
1190299-Turkey	1190777-Finland	12	31	49
1190308-Iran	1190352-Yugoslavia	11	1	83
1190308-Iran	1190398-Palestine	9	1	79
1190308-Iran	1190481-Poland	12	1	94
1190308-Iran	1190777-Finland	11	32	99
1190352-Yugoslavia	1190398-Palestine	7	145	69
1190352-Yugoslavia	1190481-Poland	10	1	58
1190352-Yugoslavia	1190777-Finland	9	1	52
1190398-Palestine	1190481-Poland	11	1	52
1190398-Palestine	1190777-Finland	7	132	55
1190481-Poland	1190777-Finland	11	1	33

Supplemental Table S23. Number of DMRs identified using pairwise comparisons for 12 Watkins accessions. DMRs in the CpG, CHG and CHH context for each pairwise comparison between the 12 Watkins accessions that were also analyzed using RNA-seq.

C context	GO.ID	Term Annotated	Significant	Expected	classic Fisher	Pval
**CpG**	GO:0030515	snoRNA binding	1	1	0.01	0.012
	GO:0008168	methyltransferase activity	17	2	0.21	0.017
	GO:0005524	ATP binding	138	5	1.71	0.019
	GO:0016741	transferase activity,	18	2	0.22	0.019
	GO:0005451	monovalent cation:proton antiporter activity	2	1	0.02	0.025
	GO:0008889	glycerophosphodiester phosphodiesterase	2	1	0.02	0.025
	GO:0015081	sodium ion transmembrane transporter activity	2	1	0.02	0.025
	GO:0015385	sodium:proton antiporter activity	2	1	0.02	0.025
	GO:0022804	active transmembrane transporter activity	22	2	0.27	0.028
	GO:0035639	purine ribonucleoside triphosphate binding	157	5	1.95	0.032
	GO:0032559	adenyl ribonucleotide binding	158	5	1.96	0.033
	GO:0030554	adenyl nucleotide binding	160	5	1.99	0.034
	GO:0004222	metalloendopeptidase activity	3	1	0.04	0.037
	GO:0015297	antiporter activity	3	1	0.04	0.037
	GO:0015298	solute:cation antiporter activity	3	1	0.04	0.037
	GO:0015299	solute:proton antiporter activity	3	1	0.04	0.037
	GO:0015491	cation:cation antiporter activity	3	1	0.04	0.037
	GO:0099516	ion antiporter activity	3	1	0.04	0.037
	GO:0005215	transporter activity	67	3	0.83	0.045
	GO:0003872	6-phosphofructokinase activity	4	1	0.05	0.049
**CHG**	GO:0004842	ubiquitin-protein transferase activity	29	1	0.04	0.036
	GO:0019787	ubiquitin-like protein transferase activity	29	1	0.04	0.036
**CHH**	GO:0018580	nitronate monooxygenase activity	2	1	0	0.002
	GO:0016703	oxidoreductase activity	8	1	0.01	0.008
	GO:2001070	starch binding	14	1	0.01	0.014
	GO:0004298	threonine-type endopeptidase activity	17	1	0.02	0.017
	GO:0070003	threonine-type peptidase activity	17	1	0.02	0.017
	GO:0003872	6-phosphofructokinase activity	21	1	0.02	0.021
	GO:0008443	phosphofructokinase activity	22	1	0.02	0.022
	GO:0016701	oxidoreductase activity	26	1	0.03	0.026
	GO:0015238	drug transmembrane transporter activity	29	1	0.03	0.029
	GO:0090484	drug transporter activity	29	1	0.03	0.029
	GO:0019200	carbohydrate kinase activity	33	1	0.03	0.033
	GO:0051213	dioxygenase activity	37	1	0.04	0.037
	GO:0001871	pattern binding	46	1	0.05	0.045
	GO:0030247	polysaccharide binding	46	1	0.05	0.045

Supplemental Table S24. Most significant GO terms from topGO GSEA of molecular functions from differentially expressed genes. Summary of topGO gene set enrichment from the differentially expressed genes that also correlated with DMRs ($p < 0.05$).

CpG	GO	Gene	Annotation
GO:0030515	snoRNA binding	TRIAE_CS42_2AS_TGACv1_114964_AA0370230	Nucleolar protein Nop56
GO:0008168	methyltransferase activity	TRIAE_CS42_1DL_TGACv1_064367_AA0234130	O-methyltransferase ZRP4
		TRIAE_CS42_7AS_TGACv1_571056_AA1843990	S-adenosylmethionine-dependent methyltransferase
GO:0005524	ATP binding	TRIAE_CS42_2DS_TGACv1_177910_AA0586990	Protein kinase-3
		TRIAE_CS42_3DL_TGACv1_251571_AA0882610	ABC transporter C family member 3
		TRIAE_CS42_5DL_TGACv1_433677_AA1419250	ATP-dependent 6-phosphofructokinase
		TRIAE_CS42_6AL_TGACv1_472145_AA1518250	Kinesin-like protein
		TRIAE_CS42_7BL_TGACv1_579573_AA1908570	ATP-dependent zinc metalloprotease FTSH 2
GO:0016741	transferase activity,	TRIAE_CS42_1DL_TGACv1_064367_AA0234130	O-methyltransferase ZRP4
		TRIAE_CS42_7AS_TGACv1_571056_AA1843990	S-adenosylmethionine-dependent methyltransferase
GO:0005451	monovalent cation:proton antiporter activity	TRIAE_CS42_2BS_TGACv1_147212_AA0479930	Sodium/hydrogen exchanger
GO:0008889	glycerophosphodiester phosphodiesterase	TRIAE_CS42_7AS_TGACv1_570588_AA1838000	Glycerophosphoryl diester phosphodiesterase
GO:0015081	sodium ion transmembrane transporter activity	TRIAE_CS42_2BS_TGACv1_147212_AA0479930	Sodium/hydrogen exchanger
GO:0015385	sodium:proton antiporter activity	TRIAE_CS42_2BS_TGACv1_147212_AA0479930	Sodium/hydrogen exchanger
GO:0022804	active transmembrane transporter activity	TRIAE_CS42_2BS_TGACv1_147212_AA0479930	Sodium/hydrogen exchanger
		TRIAE_CS42_3DL_TGACv1_251571_AA0882610	ABC transporter C family member 3
GO:0035639	purine ribonucleoside triphosphate binding	TRIAE_CS42_2DS_TGACv1_177910_AA0586990	Protein kinase-3
		TRIAE_CS42_3DL_TGACv1_251571_AA0882610	ABC transporter C family member 3
		TRIAE_CS42_5DL_TGACv1_433677_AA1419250	ATP-dependent 6-phosphofructokinase
		TRIAE_CS42_6AL_TGACv1_472145_AA1518250	Kinesin-like protein
		TRIAE_CS42_7BL_TGACv1_579573_AA1908570	ATP-dependent zinc metalloprotease FTSH 2
GO:0032559	adenyl ribonucleotide binding	TRIAE_CS42_2DS_TGACv1_177910_AA0586990	Protein kinase-3
		TRIAE_CS42_3DL_TGACv1_251571_AA0882610	ABC transporter C family member 3
		TRIAE_CS42_5DL_TGACv1_433677_AA1419250	ATP-dependent 6-phosphofructokinase
		TRIAE_CS42_6AL_TGACv1_472145_AA1518250	Kinesin-like protein
		TRIAE_CS42_7BL_TGACv1_579573_AA1908570	ATP-dependent zinc metalloprotease FTSH 2
GO:0030554	adenyl nucleotide binding	TRIAE_CS42_2DS_TGACv1_177910_AA0586990	Protein kinase-3
		TRIAE_CS42_3DL_TGACv1_251571_AA0882610	ABC transporter C family member 3
		TRIAE_CS42_5DL_TGACv1_433677_AA1419250	ATP-dependent 6-phosphofructokinase
		TRIAE_CS42_6AL_TGACv1_472145_AA1518250	Kinesin-like protein
		TRIAE_CS42_7BL_TGACv1_579573_AA1908570	ATP-dependent zinc metalloprotease FTSH 2
GO:0004222	metalloendopeptidase activity	TRIAE_CS42_7BL_TGACv1_579573_AA1908570	ATP-dependent zinc metalloprotease FTSH 2
GO:0015297	antiporter activity	TRIAE_CS42_2BS_TGACv1_147212_AA0479930	Sodium/hydrogen exchanger
GO:0015298	solute:cation antiporter activity	TRIAE_CS42_2BS_TGACv1_147212_AA0479930	Sodium/hydrogen exchanger
GO:0015299	solute:proton antiporter activity	TRIAE_CS42_2BS_TGACv1_147212_AA0479930	Sodium/hydrogen exchanger
GO:0015491	cation:cation antiporter activity	TRIAE_CS42_2BS_TGACv1_147212_AA0479930	Sodium/hydrogen exchanger
GO:0099516	ion antiporter activity	TRIAE_CS42_2BS_TGACv1_147212_AA0479930	Sodium/hydrogen exchanger
GO:0003872	6-phosphofructokinase activity	TRIAE_CS42_5DL_TGACv1_433677_AA1419250	ATP-dependent 6-phosphofructokinase
GO:0005215	transporter activity	TRIAE_CS42_2BS_TGACv1_147212_AA0479930	Sodium/hydrogen exchanger
		TRIAE_CS42_3DL_TGACv1_251571_AA0882610	ABC transporter C family member 3
		TRIAE_CS42_4DS_TGACv1_362389_AA1179390	Aquaporin SIP1-2
CHG	GO	Gene	Annotation
GO:0004842	ubiquitin-protein transferase activity	TRIAE_CS42_4AS_TGACv1_306263_AA1005360	E3 ubiquitin-protein ligase SINA-like 10
GO:0019787	ubiquitin-protein transferase activity	TRIAE_CS42_4AS_TGACv1_306263_AA1005360	E3 ubiquitin-protein ligase SINA-like 10
CHH	GO	Gene	Annotation
GO:0018580	nitronate monooxygenase activity	TRIAE_CS42_1AL_TGACv1_000157_AA0005000	2-nitropropane dioxygenase-like protein
GO:0016703	oxidoreductase activity	TRIAE_CS42_1AL_TGACv1_000157_AA0005000	2-nitropropane dioxygenase-like protein
GO:2001070	starch binding	TRIAE_CS42_2DL_TGACv1_159587_AA0540290	Soluble starch synthase 3
GO:0004298	threonine-type endopeptidase activity	TRIAE_CS42_7AS_TGACv1_570764_AA1840680	Proteasome subunit alpha type-7-A
GO:0070003	threonine-type peptidase activity	TRIAE_CS42_7AS_TGACv1_570764_AA1840680	Proteasome subunit alpha type-7-A
GO:0003872	6-phosphofructokinase activity	TRIAE_CS42_5DL_TGACv1_433677_AA1419250	ATP-dependent 6-phosphofructokinase
GO:0008443	phosphofructokinase activity	TRIAE_CS42_5DL_TGACv1_433677_AA1419250	ATP-dependent 6-phosphofructokinase
GO:0016701	oxidoreductase activity	TRIAE_CS42_1AL_TGACv1_000157_AA0005000	2-nitropropane dioxygenase-like protein
GO:0015238	drug transmembrane transporter activity	TRIAE_CS42_7DS_TGACv1_623458_AA2053510	Protein DETOXIFICATION
GO:0090484	drug transporter activity	TRIAE_CS42_7DS_TGACv1_623458_AA2053510	Protein DETOXIFICATION
GO:0019200	carbohydrate kinase activity	TRIAE_CS42_5DL_TGACv1_433677_AA1419250	ATP-dependent 6-phosphofructokinase
GO:0051213	dioxygenase activity	TRIAE_CS42_1AL_TGACv1_000157_AA0005000	2-nitropropane dioxygenase-like protein
GO:0001871	pattern binding	TRIAE_CS42_2DL_TGACv1_159587_AA0540290	Soluble starch synthase 3
GO:0030247	polysaccharide binding	TRIAE_CS42_2DL_TGACv1_159587_AA0540290	Soluble starch synthase 3

Supplemental Table S25. Genes associated with the most significant GO terms from topGO GSEA of molecular functions from differentially expressed genes. Summary of topGO gene set enrichment from the differentially expressed genes that also correlated with DMRs ($p < 0.05$).

C context	GO.ID	Term Annotated	Significant	Expected	classicFisher	Pval
**CpG**	GO:0007017	microtubule-based process	11	2	0.18	0.012
	GO:0000154	rRNA modification	1	1	0.02	0.016
	GO:0007051	spindle organization	1	1	0.02	0.016
	GO:0051225	spindle assembly	1	1	0.02	0.016
	GO:0032259	methylation	18	2	0.29	0.031
	GO:0006814	sodium ion transport	2	1	0.03	0.032
	GO:0006885	regulation of pH	2	1	0.03	0.032
	GO:0035725	sodium ion transmembrane transport	2	1	0.03	0.032
	GO:0055067	monovalent inorganic cation homeostasis	2	1	0.03	0.032
	GO:0055075	potassium ion homeostasis	2	1	0.03	0.032
	GO:0000226	microtubule cytoskeleton organization	3	1	0.05	0.047
	GO:0006002	fructose 6-phosphate metabolic process	3	1	0.05	0.047
	GO:0050801	ion homeostasis	3	1	0.05	0.047
	GO:0055065	metal ion homeostasis	3	1	0.05	0.047
	GO:0055080	cation homeostasis	3	1	0.05	0.047
	GO:0098771	inorganic ion homeostasis	3	1	0.05	0.047
**CHG**	GO:0046686	response to cadmium ion	4	1	0.01	0.0057
	GO:0010038	response to metal ion	8	1	0.01	0.0114
	GO:0010035	response to inorganic substance	16	1	0.02	0.0228
	GO:0006979	response to oxidative stress	18	1	0.03	0.0256
	GO:0006511	ubiquitin-dependent protein catabolic pr...	20	1	0.03	0.0284
	GO:0019941	modification-dependent protein catabolic...	21	1	0.03	0.0298
	GO:0043632	modification-dependent macromolecule cat...	21	1	0.03	0.0298
	GO:0044257	cellular protein catabolic process	22	1	0.03	0.0313
	GO:0051603	proteolysis involved in cellular protein...	22	1	0.03	0.0313
	GO:0030163	protein catabolic process	25	1	0.04	0.0355
	GO:0044265	cellular macromolecule catabolic process	30	1	0.04	0.0425
**CHH**	GO:0009608	response to symbiont	4	1	0	0.0036
	GO:0009610	response to symbiotic fungus	4	1	0	0.0036
	GO:0010114	response to red light	5	1	0	0.0045
	GO:0010387	COP9 signalosome assembly	6	1	0.01	0.0054
	GO:0000338	protein deneddylation	9	1	0.01	0.0081
	GO:0010388	cullin deneddylation	9	1	0.01	0.0081
	GO:0046686	response to cadmium ion	14	1	0.01	0.0125
	GO:0009620	response to fungus	19	1	0.02	0.0169
	GO:0006002	fructose 6-phosphate metabolic process	20	1	0.02	0.0178
	GO:0061615	glycolytic process through fructose-6-ph...	21	1	0.02	0.0187
	GO:0070647	protein modification by small protein co...	269	2	0.24	0.0222
	GO:0010038	response to metal ion	25	1	0.02	0.0222
	GO:0006855	drug transmembrane transport	29	1	0.03	0.0257
	GO:0015893	drug transport	29	1	0.03	0.0257
	GO:0042493	response to drug	29	1	0.03	0.0257
	GO:0009639	response to red or far red light	35	1	0.03	0.031
	GO:0042221	response to chemical	367	2	0.33	0.0396

Supplemental Table S26. Most significant GO terms from topGO GSEA of biological processes from differentially expressed genes. Summary of topGO gene set enrichment from the differentially expressed genes that also correlated with DMRs ($p < 0.05$).

Gene_id	Scaffold	AHRD_description	AHRD_GO_term	Gene expression direction
TRIAE_CS42_1BL_TGACv1_033387_AA0139100	TGACv1_scaffold_033387_1BL_2706_3863	F-box family protein with a domain of unknown function (DUF295)	NULL	Up-regulated in early heading
TRIAE_CS42_1BS_TGACv1_049362_AA0150670	TGACv1_scaffold_049362_1BS_353441_359575	Vacuolar fusion protein MON1	NULL	Up-regulated in early heading
TRIAE_CS42_1BS_TGACv1_051330_AA0179250	TGACv1_scaffold_051330_1BS_19012_24505	E3 UFM1-protein ligase 1 homolog	GO:0016874	Down-regulated in early heading
TRIAE_CS42_2AS_TGACv1_112554_AA0340870	TGACv1_scaffold_112554_2AS_124486_129983	U-box domain-containing protein	NA	Up-regulated in early heading
TRIAE_CS42_2AS_TGACv1_112749_AA0344580	TGACv1_scaffold_112749_2AS_35376_39705	UMP-CMP kinase 2	GO:0006207|GO:0006139|GO:0004127|GO:0019205|GO:0009041|GO:0005737|GO:0016740|GO:0046939|GO:0016301|GO:0006221|GO:0005634|GO:0000166|GO:0016310|GO:0005524	Up-regulated in early heading
TRIAE_CS42_2AS_TGACv1_112987_AA0349270	TGACv1_scaffold_112987_2AS_33897_35955	3-oxo-5-alpha-steroid 4-dehydrogenase	GO:0016627|GO:0016020|GO:0005737|GO:0016021|GO:0006629|GO:0055114	Up-regulated in early heading
TRIAE_CS42_2AS_TGACv1_113139_AA0351760	TGACv1_scaffold_113139_2AS_8056_10775	Glyoxylate reductase	GO:0051287|GO:0055114|GO:0016616|GO:0008152	Up-regulated in early heading
TRIAE_CS42_2BL_TGACv1_129912_AA0399300	TGACv1_scaffold_129912_2BL_47436_48490	Chromatin assembly factor 1 subunit FSM	GO:0010449|GO:0006281|GO:0005634|GO:0016568|GO:0010389|GO:0044772|GO:0006351|GO:0006974|GO:0006310|GO:0006334|GO:0010448|GO:0033186|GO:0006355	Down-regulated in early heading
TRIAE_CS42_2BL_TGACv1_133146_AA0441590	TGACv1_scaffold_133146_2BL_10266_14115	Thiol-disulfide oxidoreductase DCC	NULL	Down-regulated in early heading
TRIAE_CS42_2BS_TGACv1_145883_AA0448030	TGACv1_scaffold_145883_2BS_305296_307292	Nodulin MtN21 /EamA-like transporter family protein	NULL	Up-regulated in early heading
TRIAE_CS42_5AL_TGACv1_375998_AA1230410	TGACv1_scaffold_375998_5AL_49587_53176	Proteasome subunit alpha type	GO:0006508|GO:0005634|GO:0000502|GO:0016787|GO:0019773|GO:0005839|GO:0004298|GO:0004175|GO:0005737|GO:0051603|GO:0006511|GO:0008233	Down-regulated in early heading
TRIAE_CS42_5AS_TGACv1_729465_AA2172150	TGACv1_scaffold_729465_5AS_1_4499	Plasma membrane ATPase	GO:0016021|GO:0000166|GO:1902600|GO:0016787|GO:0005524|GO:0006754|GO:0016887|GO:0008553|GO:0006810|GO:0015992|GO:0046872|GO:0016020|GO:0006811	Up-regulated in early heading
TRIAE_CS42_5BL_TGACv1_404337_AA1296100	TGACv1_scaffold_404337_5BL_82982_85020	Papain-like cysteine proteinase	GO:0016787|GO:0006508|GO:0008234|GO:0008233	Up-regulated in early heading
TRIAE_CS42_5DS_TGACv1_457688_AA1488920	TGACv1_scaffold_457688_5DS_10336_15028	NA	NA	Up-regulated in early heading
TRIAE_CS42_6BL_TGACv1_499908_AA1594400	TGACv1_scaffold_499908_6BL_62922_65065	Glycosyltransferase	GO:0016757|GO:0008152|GO:0016740|GO:0016758	Down-regulated in early heading
TRIAE_CS42_6BL_TGACv1_500631_AA1607640	TGACv1_scaffold_500631_6BL_27266_31330	26S protease regulatory subunit 7	NULL	Down-regulated in early heading
TRIAE_CS42_6BS_TGACv1_513422_AA1641120	TGACv1_scaffold_513422_6BS_111823_115543	Cytochrome P450 71D7	GO:0055114|GO:0004497|GO:0046872|GO:0020037|GO:0005506|GO:0016491|GO:0016705	Up-regulated in early heading
TRIAE_CS42_6BS_TGACv1_516837_AA1676310	TGACv1_scaffold_516837_6BS_10420_12931	NA	NA	Up-regulated in early heading
TRIAE_CS42_7AS_TGACv1_569656_AA1821230	TGACv1_scaffold_569656_7AS_33254_35623	NA	NA	Up-regulated in early heading
TRIAE_CS42_7AS_TGACv1_570087_AA1830070	TGACv1_scaffold_570087_7AS_13811_16262	Ozone-responsive stress-related protein	NULL	Down-regulated in early heading
TRIAE_CS42_7AS_TGACv1_570420_AA1835460	TGACv1_scaffold_570420_7AS_39332_42412	Geranylgeranyl transferase type-2 subunit beta	GO:0003824|GO:0004663|GO:0016740|GO:0018344	Down-regulated in early heading
TRIAE_CS42_7BL_TGACv1_576896_AA1859020	TGACv1_scaffold_576896_7BL_64233_65705	NA	NA	Up-regulated in early heading
TRIAE_CS42_7BL_TGACv1_576925_AA1860100	TGACv1_scaffold_576925_7BL_173411_177587	Receptor-kinase	GO:0016020|GO:0032440|GO:0004672|GO:0004674|GO:0016740|GO:0016491|GO:0016301|GO:0005524|GO:0016310|GO:0055114|GO:0006468|GO:0000166|GO:0016021	Up-regulated in early heading
TRIAE_CS42_7BL_TGACv1_578170_AA1890500	TGACv1_scaffold_578170_7BL_4906_9503	Polyubiquitin	GO:0005634|GO:0005737	Down-regulated in early heading
TRIAE_CS42_7BL_TGACv1_578342_AA1893260	TGACv1_scaffold_578342_7BL_58528_63370	60S acidic ribosomal protein P0	GO:0005840|GO:0005622|GO:0042254|GO:0030529	Up-regulated in early heading
TRIAE_CS42_7BL_TGACv1_578405_AA1894240	TGACv1_scaffold_578405_7BL_70976_72282	NA	NA	Up-regulated in early heading
TRIAE_CS42_7BL_TGACv1_579133_AA1904510	TGACv1_scaffold_579133_7BL_29069_34114	60S acidic ribosomal protein P0	GO:0030529|GO:0042254|GO:0005622|GO:0005840	Up-regulated in early heading
TRIAE_CS42_7BL_TGACv1_580078_AA1911910	TGACv1_scaffold_580078_7BL_12595_16590	NA	NA	Up-regulated in early heading
TRIAE_CS42_7BS_TGACv1_591790_AA1921260	TGACv1_scaffold_591790_7BS_291937_296405	Serine/threonine-protein phosphatase	GO:0006470|GO:0004721|GO:0016787	Down-regulated in early heading
TRIAE_CS42_7BS_TGACv1_591919_AA1925610	TGACv1_scaffold_591919_7BS_236086_239655	Cysteine-rich PDZ-binding	NULL	Down-regulated in early heading
TRIAE_CS42_7BS_TGACv1_592239_AA1934050	TGACv1_scaffold_592239_7BS_60147_63155	vesicle-associated membrane protein 713	NULL	Down-regulated in early heading
TRIAE_CS42_7BS_TGACv1_592865_AA1945470	TGACv1_scaffold_592865_7BS_20056_27270	Receptor-like protein kinase	GO:0000166|GO:0016021|GO:0016310|GO:0005524|GO:0006468|GO:0016301|GO:0004674|GO:0016740|GO:0004672|GO:0016020	Down-regulated in early heading
TRIAE_CS42_7BS_TGACv1_593481_AA1951940	TGACv1_scaffold_593481_7BS_14360_21160	ENTH/ANTH/VHS superfamily protein	NULL	Down-regulated in early heading
TRIAE_CS42_7BS_TGACv1_594998_AA1958660	TGACv1_scaffold_594998_7BS_5514_6932	NA	NA	Down-regulated in early heading
TRIAE_CS42_7DL_TGACv1_602658_AA1964290	TGACv1_scaffold_602658_7DL_30035_39367	MYB-related transcription factor-REVEILLE 8-like, LHY-CCA1-like 5	GO:0003677|GO:0006351|GO:0006355|GO:0005634	Down-regulated in early heading
TRIAE_CS42_7DS_TGACv1_622088_AA2032510	TGACv1_scaffold_622088_7DS_51109_53860	Cysteine synthase	GO:0006535|GO:0004124	Down-regulated in early heading
TRIAE_CS42_U_TGACv1_640739_AA2072060	TGACv1_scaffold_640739_U_78767_91304	Nuclear inhibitor of protein phosphatase 1	NULL	Down-regulated in early heading
TRIAE_CS42_U_TGACv1_673344_AA2156200	TGACv1_scaffold_673344_U_754_1635	NA	NA	Up-regulated in early heading
TRIAE_CS42_U_TGACv1_688173_AA2160140	TGACv1_scaffold_688173_U_274_1275	NA	NA	Up-regulated in early heading
N/A	TGACv1_scaffold_116893_2AS_2572_7016	beta-glucosidase 16-like	NA	Up-regulated in early heading
N/A	TGACv1_scaffold_030250_1BL_296703_302190	fatty acid desaturase DES2-like	NA	Up-regulated in early heading
N/A	TGACv1_scaffold_392779_5AS_31781_35263	FAR1-RELATED SEQUENCE 5-like	NA	Down-regulated in early heading
N/A	TGACv1_scaffold_500949_6BL_6998_11590	ethylene response factor 1 (ERF1)	NA	Up-regulated in early heading
N/A	TGACv1_scaffold_435529_5DL_2376_7151	Methyltransferase	NA	Up-regulated in early heading

N/A	TGACv1_scaffold_327865_4 BS_322240_326686	CASP-like protein	NA	Up-regulated in early heading
N/A	TGACv1_scaffold_592662_7 BS_12832_17860	HMW glutenin A gene locus	NA	Up-regulated in early heading

Supplemental Table S27. Differentially expressed genes between accessions with early and late heading dates. Differentially expressed genes identified between accessions 1190209/1190034 and 1190481/1190181 in a pairwise comparison matrix**.** Detailing only those genes with annotation.

GO.ID	Term Annotated	Significant	Expected	classicFisher	pval
GO:0010448	vegetative meristem growth	1	1	0	0.0017
GO:0080186	developmental vegetative growth	1	1	0	0.0017
GO:0018342	protein prenylation	2	1	0	0.0034
GO:0018344	protein geranylgeranylation	2	1	0	0.0034
GO:0097354	prenylation	2	1	0	0.0034
GO:0000086	G2/M transition of mitotic cell cycle	5	1	0.01	0.0085
GO:0010389	regulation of G2/M transition	5	1	0.01	0.0085
GO:0044839	cell cycle G2/M phase transition	5	1	0.01	0.0085
GO:1902749	regulation of cell cycle G2/M phase transition	5	1	0.01	0.0085
GO:0042254	ribosome biogenesis	83	2	0.14	0.0085
GO:0007346	regulation of mitotic cell cycle	7	1	0.01	0.0119
GO:0010449	root meristem growth	7	1	0.01	0.0119
GO:0044770	cell cycle phase transition	7	1	0.01	0.0119
GO:0044772	mitotic cell cycle phase transition	7	1	0.01	0.0119
GO:1901987	regulation of cell cycle phase transition	7	1	0.01	0.0119
GO:1901990	regulation of mitotic cell cycle phase transition	7	1	0.01	0.0119
GO:0006207	'de novo' pyrimidine nucleobase biosynthesis	11	1	0.02	0.0186
GO:0010564	regulation of cell cycle process	11	1	0.02	0.0186
GO:0022613	ribonucleoprotein complex biogenesis	128	2	0.22	0.0195
GO:0009165	nucleotide biosynthetic process	140	2	0.24	0.0231
GO:1901293	nucleoside phosphate biosynthetic process	140	2	0.24	0.0231
GO:0019856	pyrimidine nucleobase biosynthetic process	14	1	0.02	0.0236
GO:0044085	cellular component biogenesis	381	3	0.65	0.0251
GO:0035266	meristem growth	16	1	0.03	0.0269
GO:0006206	pyrimidine nucleobase metabolic process	20	1	0.03	0.0335
GO:0006535	cysteine biosynthetic process	24	1	0.04	0.0401
GO:0006310	DNA recombination	25	1	0.04	0.0417
GO:0046112	nucleobase biosynthetic process	25	1	0.04	0.0417
GO:0006334	nucleosome assembly	27	1	0.05	0.045
GO:0031497	chromatin assembly	27	1	0.05	0.045
GO:0048507	meristem development	27	1	0.05	0.045
GO:0006323	DNA packaging	28	1	0.05	0.0466
GO:0006333	chromatin assembly or disassembly	28	1	0.05	0.0466
GO:0034728	nucleosome organization	28	1	0.05	0.0466
GO:1903047	mitotic cell cycle process	28	1	0.05	0.0466
GO:0019344	cysteine biosynthetic process	29	1	0.05	0.0482
GO:0006534	cysteine metabolic process	30	1	0.05	0.0499
GO:0090407	organophosphate biosynthetic process	215	2	0.37	0.0507
GO:0000278	mitotic cell cycle	33	1	0.06	0.0547
GO:0009112	nucleobase metabolic process	33	1	0.06	0.0547

Supplemental Table S28. Most significant GO terms from topGO GSEA of biological processes from differentially expressed genes between accessions with early and late heading dates. Summary of topGO gene set enrichment from the differentially expressed genes between accessions 1190209/1190034 and 1190481/1190181 in a pairwise comparison matrix (TopGO, $p < 0.05$, weight01 scoring for Fisher's exact test). GOterm analysis using 28,554 gene universe as a background (genes detected across all 4 replicate sets, with variance >1)

Type	Mapping Coverage	Percentage of total (%)	Brenchley et al.[32] Percentage of total (%)
**DNA transposons**	**8644544**	**18.027**	**18.691**
Helitron	45572	0.095	0.303
TIR	8588300	17.910	18.311
HAT	1500	0.003	0.052
Harbinger	516673	1.077	0.427
Mariner	147201	0.307	0.128
CACTA	7239357	15.097	15.995
Mutator	683569	1.426	0.557
Unknown	10672	0.022	0.011
**Retrotransposons**	**39308976**	**81.973**	**79.779**
SINE	10225686	21.324	0.005
LTR	29083290	60.649	78.748
Gypsy	19481153	40.625	44.034
Copia	7712505	16.083	17.394
Unknown	1889632	3.941	1.490

Supplemental Table S29. Repeat composition of Chinese Spring. Enriched bisulfite treated sequencing reads from Chinese Spring were aligned to the TREP repeat database using our standard alignment workflow (see methods). The total aligned base-space is detailed here per transposable element (TE) group with the % of the total transposon database hit. The same analysis performed by Brenchley *et al.*[32] on non-enriched wheat is also shown.

Accession	% of methylated cytosines across TE CpG sites	% of methylated cytosines across TE CHG sites	% of methylated cytosines across TE CHH sites	Number of reads aligning to TEs
1190007	81.4	55.4	2.7	10227682
1190032	82.1	59.6	3	10019328
1190034	82.1	59.8	2.8	17957264
1190040	82.6	59.1	2.9	11571750
1190042	82.9	57.7	2.8	9438914
1190044	81.8	57.5	2.8	14195980
1190045	83.1	60.7	4.6	9822402
1190079	82.2	59.4	3.9	10816496
1190092	81.8	57.7	3.4	10343708
1190103	81.6	56.1	3	39431722
1190110	82.2	57.2	3.5	10139388
1190126	83.8	62.1	4.1	9021308
1190127	81.1	57.7	3	15564764
1190139	81.6	56.4	2.9	15081944
1190141	82.4	57.5	3.3	41863722
1190145	81.7	59.1	3	13097394
1190149	82.1	59.2	3.2	9689396
1190160	81.9	58.1	2.7	9116158
1190166	81.6	57.7	2.9	9891256
1190181	82.5	60.3	3.3	16495582
1190199	83.5	60.9	3.4	10332882
1190209	82.4	57.4	4	10565974
1190216	82.1	58.9	3.2	9561718
1190218	82.4	58.9	3.6	8840712
1190219	83.6	59.2	4.4	7467302
1190223	82.4	57.6	2.8	20321462
1190224	81.1	58.5	3.3	15648780
1190231	80.6	58.2	3.1	11924524
1190238	80.9	53.5	2.8	45000570
1190239	81.5	56.7	3.3	13187952
1190246	79.8	53.4	2.9	10940460
1190254	81.6	57.5	2.9	11592064
1190264	81.5	59.1	3.3	19534652
1190273	82	57.8	4	14560010
1190281	80.8	58	4	13845326
1190291	82.3	58.9	3.5	9164498
1190292	81.9	57.5	3.2	39497116
1190299	81.4	57.2	2.9	15262814
1190300	82.3	58.8	3.5	9644948
1190308	81.9	54.4	3.2	41105626
1190313	81.9	58.4	3.7	7685344
1190324	82.6	60.4	2.8	19930672
1190325	82.7	61.4	2.9	12802858
1190349	81.7	57.5	2.7	10802364
1190352	82.5	58.5	3.1	10051694
1190355	82.5	57.5	2.9	10737632
1190360	81.3	55.3	2.7	9916498
1190387	81.8	59.2	3.4	14722772
1190396	82.5	58.4	3	13149806
1190398	81.9	57.7	3.9	11172614
1190406	82.1	58.8	3.6	9874632
1190420	80.7	58.4	3.1	9926252
1190433	81.6	53	3.6	8383384
1190440	82.1	57	2.7	17360288
1190451	81.2	59	3	13511552

1190460	82.2	56.3	3	6151610
1190468	80.9	53.9	2.6	11347818
1190471	80.9	48.3	2.7	9657330
1190474	82.2	57.1	2.9	10639402
1190475	82.9	59.6	3	23022868
1190481	82	59.7	4	11488806
1190483	80.7	57.2	3.4	11693694
1190496	81.8	58.1	2.9	11075604
1190507	81.6	58.2	3.4	9954056
1190546	82.4	60	3.7	8757278
1190551	82	57.7	2.8	14660032
1190560	82.4	58.8	2.7	11302132
1190562	81.8	57.8	2.7	9928928
1190579	82.1	61.3	3	9686370
1190580	82.2	57.4	2.9	9822578
1190591	83.2	62.4	3.1	9901610
1190624	82.7	58.6	4.2	12500064
1190627	82.4	58.7	3.3	41431398
1190629	81.3	58.9	4.6	9985962
1190637	81.5	58.2	3.3	10128238
1190639	82.1	58.4	3.1	10260296
1190651	82.2	62.3	3.3	8498286
1190652	81.4	57.5	3.7	7766962
1190662	82.2	59.1	2.7	13931354
1190670	82	59.2	2.8	13215054
1190671	81.6	56.6	2.9	10132526
1190683	81.4	57.1	2.6	9972534
1190685	81.8	56.7	2.7	9544964
1190690	81.8	57.3	3	9493718
1190694	83.3	59.3	3.7	10360296
1190698	83.1	60	4.4	10236214
1190700	82.6	58.6	3.5	11394990
1190704	81.8	57.4	3.4	12089502
1190705	82.4	57.3	3.6	10452966
1190707	82	57.3	3.8	7639324
1190722	83	58.4	2.7	14308232
1190731	82.9	59.7	3.1	11104720
1190740	83.6	60.6	2.9	9784492
1190742	81.1	56.4	2.5	10281188
1190746	82.4	58.1	3	12158030
1190747	82.2	58.3	3.2	11849406
1190749	82.5	60.6	2.9	11697190
1190753	81.3	57.6	2.9	9966678
1190771	81.8	58.9	3.2	10898758
1190777	82.6	61	3.3	11432508
1190784	82.5	58.9	3.2	10301716
1190788	81.9	59.9	3.2	8321834
1190811	83.1	50.4	2.9	40874466
1190827	82.5	56.2	3.2	44293654
CS	83.1	52.9	3.2	39865486

Supplemental Table S30. Methylation levels across the TE TREP database for the 104 accessions from the core set of the Watkins bread wheat landrace collection plus Chinese Spring. Detailing the percentages of methylated cytosines found at CpG, CHG and CHH sites and also the number of aligned reads used to make these calls.

Supplemental References

Apse MP, Aharon GS, Snedden WA and Blumwald E. 1999. Salt tolerance conferred by overexpression of a vacuolar Na+/H+ antiport in Arabidopsis. *Science,* 285(5431): 1256-8

Bernardes JS, Vieira FRJ, Zaverucha G and Carbone A. 2016. A multi-objective optimization approach accurately resolves protein domain architectures, *Bioinformatics* 32 (3): 345-353

Emms DM and Kelly S. 2015. OrthoFinder: solving fundamental biases in whole genome comparisons dramatically improves orthogroup inference accuracy, Genome Biology, 16:157

Finn RD, Coggill P, Eberhardt RY, Eddy SR, Mistry J, Mitchell AL, Potter SC, Punta M, Qureshi M, Sangrador-Vegas A et al. 2016. The Pfam protein families database: towards a more sustainable future. *Nucleic Acids Res.* 44 (D1): D279-D285

Genereux DP, Johnson WC, Burden AF, Stöger R and Laird CD. 2008. Errors in the bisulfite conversion of DNA: modulating inappropriate and failed conversion frequencies. *nar.oxfordjournals.org*

Goodstein DM, Shu S, Howson R, Neupane R, Hayes RD, Fazo j, Mitros T, Dirks W, Hellsten U, Putnam N and Rokhsar DS. 2012. Phytozome: a comparative platform for green plant genomics, *Nucleic Acids Res.* 40:D1178-86

Leache AD, Banbury BL, Felsenstein J, Oca AN and Stamatakis A. 2015. Short tree, Long tree, Right tree, Wrong tree: New Acquisition Bias Corrections for Inferring SNP Phylogenies. Syst. Biol. 64(6): 1032-1047

Lischer HEL, Excoffier L and Heckel G. 2013. Ignoring Heterozygous Sites Biases Phylogenomic Estimates of Divergence Times: Implications for the Evolutionary History of *Microtus* Voles. Molecular Biology and Evolution 31(4): 817-831

Mascher M, Gundlach H, Himmelbach A, Beier S, Twardziok SO, Wicker T, Radchuk V, Dockter C, Hedley PE, Russell J et al. 2017. A Chromosome Conformation Capture Ordered Sequence of the Barley Genome, *Nature* 544 (7651), 427-433

Ottesen RT. et al. 2013. Mercury in European agricultural and grazing land soils, *Applied Geochemistry,* 33;1-12

Ono T. et al. 2006. Chromatin assembly factor 1 ensures the stable maintenance of silent chromatin states in Arabidopsis. *Genes to Cells,* 11(2); 152-162

Reimann C. et al. 2014. *European Atlas of Natural Radiation.* [online] Remon.jrc.ec.europa.eu. Available at: https://remon.jrc.ec.europa.eu/About/Atlas-of-Natural-Radiation/ [Accessed 14 Jul. 2017]

Steinig EJ, Neuditschko M, Khatkar MS, Raadsma HW and Zenger KR. 2016. NETVIEW P: a network visualization tool to unravel complex population structure using genome-wide SNPs. Molecular ecology resources 16, 216-227

Szabolcs I. 1974. Salt-affected soils in Europe. *The Hague, Martinus Nihoff.* 63p

Wald A and Wolfowitz J. 1940. On a test whether two samples are from the same population, Ann. Math Statist. 11, 147-162
